# Natural Products from Actinomycetes Associated with Marine Organisms

**DOI:** 10.3390/md19110629

**Published:** 2021-11-10

**Authors:** Jianing Chen, Lin Xu, Yanrong Zhou, Bingnan Han

**Affiliations:** Department of Development Technology of Marine Resources, College of Life Sciences and Medicine, Zhejiang Sci-Tech University, Hangzhou 310018, China; 202020801006@mails.zstu.edu.cn (J.C.); linxu@zstu.edu.cn (L.X.); zhouyanrong@zstu.edu.cn (Y.Z.)

**Keywords:** actinomycetes, marine animals, marine plants, macroalgae, secondary metabolites, structural diversity, biological activities

## Abstract

The actinomycetes have proven to be a rich source of bioactive secondary metabolites and play a critical role in the development of pharmaceutical researches. With interactions of host organisms and having special ecological status, the actinomycetes associated with marine animals, marine plants, macroalgae, cyanobacteria, and lichens have more potential to produce active metabolites acting as chemical defenses to protect the host from predators as well as microbial infection. This review focuses on 536 secondary metabolites (SMs) from actinomycetes associated with these marine organisms covering the literature to mid-2021, which will highlight the taxonomic diversity of actinomycetes and the structural classes, biological activities of SMs. Among all the actinomycetes listed, members of *Streptomyces* (68%), *Micromonospora* (6%), and *Nocardiopsis* (3%) are dominant producers of secondary metabolites. Additionally, alkaloids (37%), polyketides (33%), and peptides (15%) comprise the largest proportion of natural products with mostly antimicrobial activity and cytotoxicity. Furthermore, the data analysis and clinical information of SMs have been summarized in this article, suggesting that some of these actinomycetes with multiple host organisms deserve more attention to their special ecological status and genetic factors.

## 1. Introduction

Actinomycetes are Gram-positive bacteria with a GC-rich linear genome and have proven to be a rich source of secondary metabolites (SMs) of broad structural diversity and biological properties [1]. The ocean has been demonstrated as an ecosystem with many unique forms of actinomycetes [2]. The diversity of marine actinomycetes is determined by the uniqueness of the marine environment: some live freely in seawater, some in the seafloor sediments or sea mud; and some are symbiotic, parasitic, endophytic, or epiphytic with marine organisms [2,3]. Compared with actinomycetes isolated from seawater and sediment samples, recent reports of secondary metabolites from marine actinomycetes associated with a variety of aquatic organisms, including invertebrates such as sponges, corals, ascidians, echinoderms, and vertebrates such as pufferfish, as well as algae and seaweed, have increased significantly [4]. Studies have indicated that multiple active compounds previously isolated from marine invertebrates were possibly produced by their symbiotic microorganisms, especially actinomycetes [5,6,7]. With interactions of the host and having special ecological status, the actinomycetes associated with marine organisms have more potential to produce active metabolites acting as chemical defenses to protect the host from predators and microbial infection.

The objective of this article is to provide an overview of the natural products from actinomycetes associated with marine animals, marine plants, macroalgae, cyanobacteria, and lichens. The present review was not only summarizing the structural classes and biological activities of SMs but also highlighted the taxonomic diversity of actinomycetes, as well as the data analysis of integrated above information. Some of these metabolites with excellent activity are expected to become new drugs such as antibiotics, antineoplastic drugs, or anticancer drugs. Therefore, actinomycetes with multiple host organisms deserve more attention to their special ecological status and genetic factors.

## 2. Biology of Actinomycetes Associated with Marine Animals, Marine Plants, Macroalgae, Cyanobacteria and Lichens

Marine actinomycetes are abundant in species and widely inhabit sediments, seawater, and aquatic organisms. At present, these actinomycetes are mainly separated from marine invertebrates especially sponges, ascidians, corals as well as brown algae. It is presently estimated that only 1% of microbes can be separated using traditional culturing techniques, making the potential for this field more compelling [2,6]. However, emerging technologies provide us with the tools to determine overall microbial diversity. The Ribosomal Database Project (RDP) classification of 16S rRNA sequences of marine organism-associated actinomycetes in 2014, revealed 136 genera within the subclass of Acidimicrobidae, Actinobacteridae, and Rubrobacteridea, of which *Streptomyces*, *Micromonospora*, *Microbacterium,* and *Nocardiopsis* were abundant [8]. In addition, rare actinomycetes including *Actinoalloteichus*, *Agromyces*, *Agrococcus*, *Amycolatopsis*, *Nonomuraea*, *Phycicoccus*, *Saccharothrix,* and *Serinicoccus* were discovered from these marine organisms [8]. And uncultured actinomycetes such as *Iamia*, *Aciditerrimonas,* and *Illumatobacter* were also detected [8]. As the diversity of metabolites is closely related to biodiversity, the potential for obtaining abundant and novel SMs from actinomyces associated with various marine hosts is relatively high.

**Figure 1:**
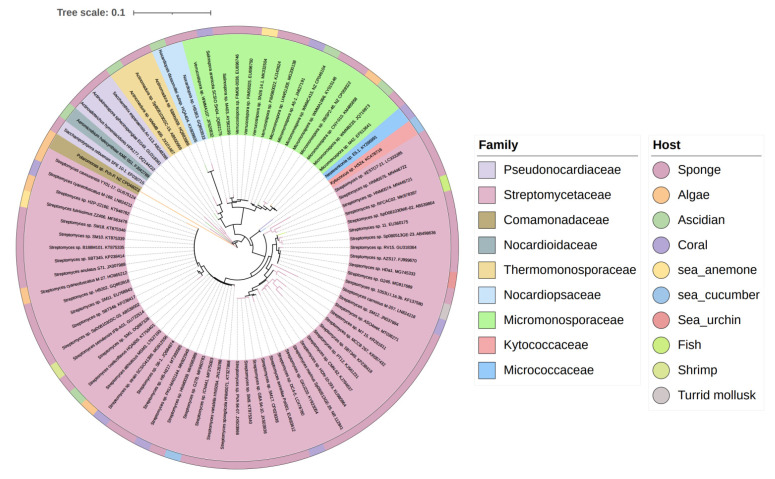
Neighbor-Joining phylogenetic tree based on 16S rRNA gene sequences of natural product-producing actinomycetes. The aligned sequences were analyzed by the bootstrap method with a bootstrap number of 10,000. The colored labels indicate the classification of actinomycetes; the outer color strip shows their host sources.

The biosynthesis ability of actinomycetes in the production of complex natural products has been known for a long time. Not all actinomycetes, however, are prolific secondary metabolite producers. To assess the diversity and distribution of natural product-producing actinomycetes associated with various marine hosts, we constructed a neighbor-joining phylogenetic tree of 16S rRNA gene sequences. We obtained these associated actinomycetes producing natural products from literature and the antiSMASH database and selected strains with a length of 16S rRNA sequences > 900 bp that were available in the NCBI database to construct the phylogenetic tree. The phylogenetic tree involved 84 strains within nine families, and the family Streptomycetaceae (*Streptomyces*) represents 67% of the actinomycetes, followed by *Micromonosporaceae* (*Salinispora* and *Micromonospora*), Pseudonocardiaceae (*Saccharopolyspora*) with 18%, 5% of actinomycetes, respectively, indicating the better potential of those three families to produce SMs (Figure 1). Currently, actinomycetes that produce natural products are mainly isolated from sponges. Therefore, taking sponge-derived actinomycetes as an example, almost half of the genera belong to Micrococcineae, while their potential for secondary metabolism appears to be limited to few reports. On the contrary, Streptomycetaceae, *Micromonosporaceae*, and Pseudonocardiaceae with low abundance in sponges have more potential for secondary metabolism, which reveals the clinical and pharmaceutical importance of these three families for providing novel lead compounds [9].

## 3. Chemical Structures and Biological Properties of the Actinomycetes Associated with Marine Animals, Marine Plants, Macroalgae, Cyanobacteria, and Lichens

### 3.1. Natural Products of the Actinomycetes Derived from Marine Animals

Among the 34 animal phyla known on the earth, marine animals account for 33 species, 15 of which are unique to the ocean. Studies have shown that some symbiotic microorganisms are species-specific [8], indicating that marine animals may be rich in microbial resources. And the active substances in marine animals are mostly produced by their associated microorganisms, of which actinomycetes are an important group [5,6,7]. Therefore, actinomycetes associated with marine animals are a flourishing source for novel natural products. (The structure of compounds with ‘S’ added before the number is shown in supporting information.)

#### 3.1.1. Alkaloids

Alkaloids are a kind of nitrogen-containing organic compounds existing in nature, which have alkali-like properties. They are secondary metabolites with significant biological activities, most of which have complex ring structures and the nitrogen is mostly contained in the ring.

##### Alkaloids Derived from the Sponge-Associated Actinomycetes

**Figure 2:**
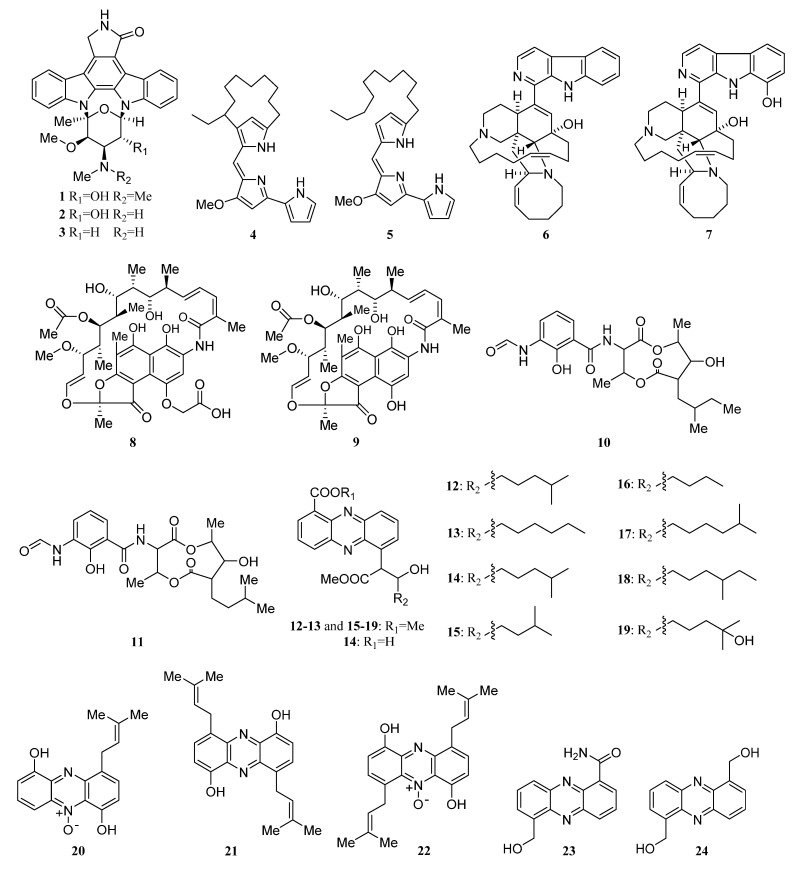

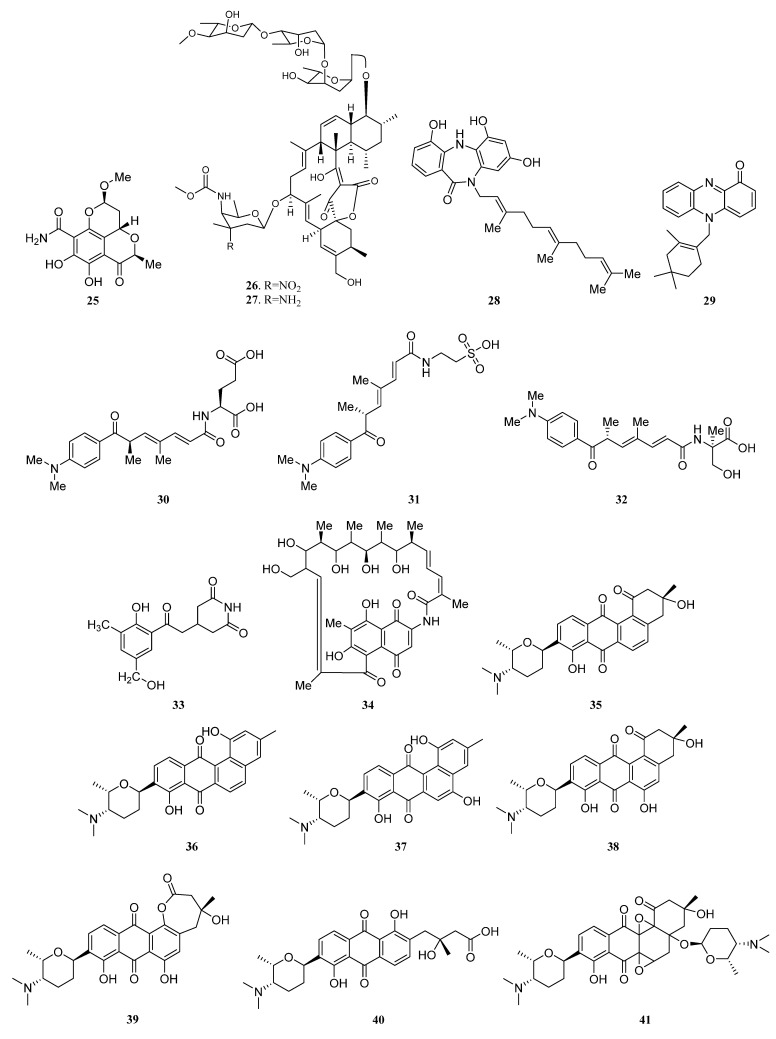

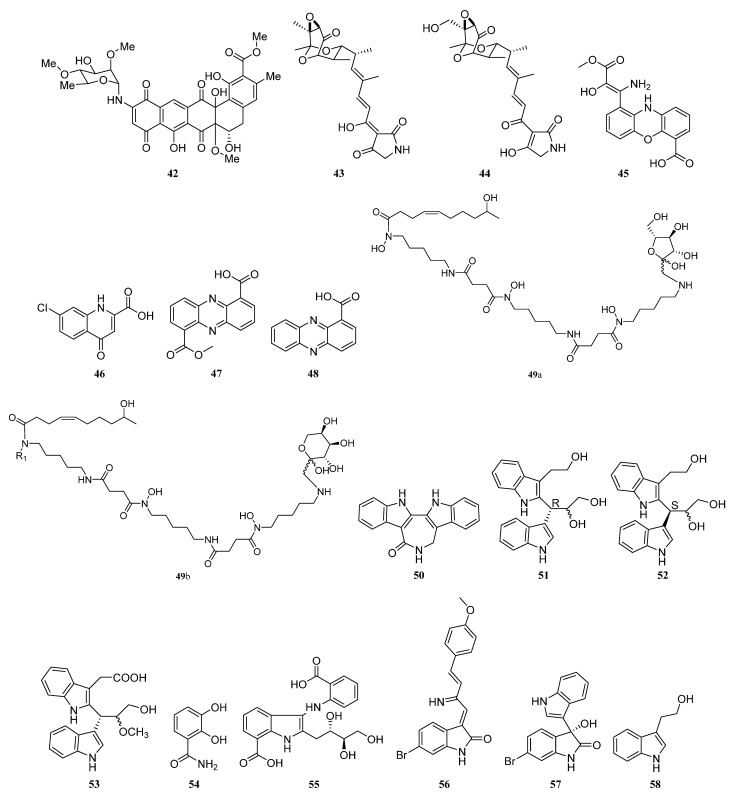

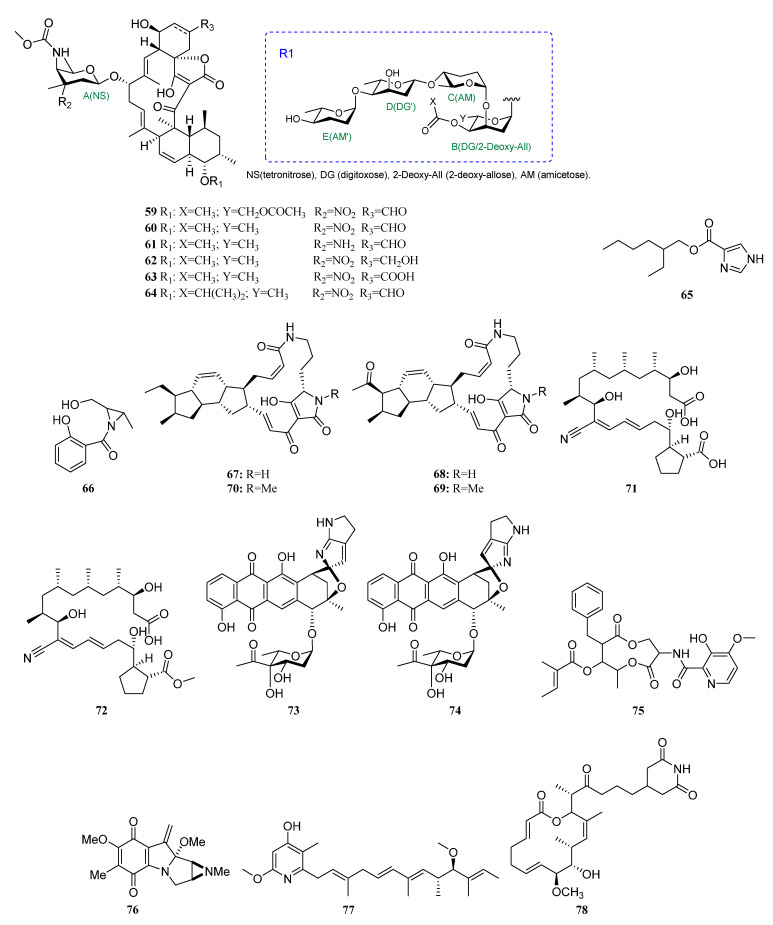
Structures of compounds **1**–**78**.

Two novel indolocarbazole alkaloids, 4′-N-methyl-5′-hydroxystaurosporine (**1**) and 5′-hydroxystaurosporine (**2**), as well as the known staurosporine (**3**) (Figure 2) were purified from the culture broth of *Micromonospora* sp. L-31-CLCO-002, which was associated with marine sponge *Clathrina coriacea* collected offshore Fuerteventura (Canary Islands). These compounds displayed cytotoxicity against various tumor cell lines [9,10]. The analysis of structure-activity relationships of staurosporine and its derivatives demonstrated that hydroxylation at C-3 of the indolocarbazole moiety led to the increase in anti-proliferative activity, while hydroxylation at C-11 caused a decrease in activity. The results suggested that not only the presence/absence of hydrophilic substitutions but also the position of the alteration within the molecule is significant in the anti-proliferative activities of the various staurosporine analogs [11,12].

In 2005, an ongoing screening program for in vitro anticancer microbial extracts led to the discovery of two prodigiosin analogs metacycloprodigiosin (**4**) and undecylprodigiosin (**5**) (Figure 2) from a CHCl_3_ extract of a strain of *Saccharopolyspora* sp. nov., which was associated with the marine sponge *Mycale plumose* collected on the coast of Qingdao, China. Both compounds **4** and **5** showed potent in vitro cytotoxicity against five cancer cell lines (P388, HL60, A-549, BEL-7402, and SPCA4), which was the first report on metacycloprodigiosin with anti-cancer activity [13]. The *Micromonospora* sp. strain M42 obtained from Indonesian sponge *Acanthostrongylophora* sp. produced manzamine A (**6**) and 8-hydroxy manzamine (**7**) (Figure 2), which had demonstrated activities against malaria, tuberculosis, and HIV [9].

Isolation of two rifamycins B and SV (**8**, **9**) (Figure 2) was reported in 2006 from the *Salinispora* sp. strain M403 associated with sponge *Pseudoceratina clavata*. This is the first recorded source of rifamycins from marine bacteria and *Salinispora* sp. represents a potential new source of rifamycins outside the genus *Amycolatopsis*. Rifamycins are a group of antibiotics that belong to the ansamycin family with pronounced activities against Gram-positive bacteria [9,14]. The structure and activity relationship of rifamycins with many different targets have been extensively studied [15,16]. The rifamycin SV has been successfully widely used in the clinical treatment of tuberculosis, leprosy, and mycobacterial infections.

Urauchimycins A and B (**10**, **11**) (Figure 2) were the first antimycin type antibiotics that possess a branched side chain and the side chain contains an odd number of carbons. The two novel antimycins were obtained from the *Streptomyces* sp. strain Ni-80 cultivated from an unidentified sponge collected at Urauchicove, Iriomote, Japan. They showed antifungal activity against *Candida albicans* by inhibiting the morphological differentiation of *Candida albicans* [9,17].

New phenazines streptophenazines A-H (**12**–**19**) (Figure 2) were isolated from *Streptomyces* sp. strain HB202 cultivated from the marine sponge *Halichondria panicea* collected from the Baltic Sea (Germany). Streptophenazines **12**–**14**, **17**, and **18** showed a wide range of antibacterial activity against both Gram-positive and Gram-negative bacteria, while compounds **15**, **16**, and **19** were only against *B. subtilis* [18]. In addition, streptophenazine B exhibited weak cytotoxicity against both cancer cells and normal cells [19].

In 2008, two novel macrolactams cebulactams A1 and A2 (**S1**, **S2**) featured with a six-membered cyclic ether as part of the macrocycle were obtained from the *Saccharopolyspora cebuensis* SPE 10-1 associated with the sponge *Haliclona* sp. which was collected from Cebu, Philippines [9,20]. The indolocarbazole alkaloid staurosporine (**3**) was purified from *Streptomyces* sp. strain 11, which had been cultivated from the sponge *Tedania* sp. It displayed significant anti-parasitic activity against *Leishmania major* and *Trypanosoma brucei brucei* with IC_50_ values of 5.30 and 0.022 μM, respectively. In addition, staurosporine also exhibited general cytotoxicity against 293T kidney epithelial cells (IC_50_ 1.30 µM) and J774.1 macrophages (IC_50_ < 0.13 µM) [9,21].

A new anthracycline 5-iminoaranciamycin (**S3**) was separated from *Streptomyces* sp. strain Sp080513GE-26 associated with the sponge *Haliclona* sp. collected from Tateyama, Japan [9,22]. Three novel isoprenoids JBIR 46–48 (**20**–**22**) (Figure 2), phenazine derivatives harboring dimethylallyl moieties, were produced by *Streptomyces* sp. SpC080624SC-11 cultivated from the sponge *Cinachyra* sp. collected from the seashore at Nagura Bay, Ishigaki, Japan [9,23]. JBIR 46–48 displayed cytotoxic activity against HL-60 cells (IC_50_ = 189, 226 and 96 μM, respectively) [24].

Two new antibacterial phenazines, 6-hydroxymethyl-1-phenazine- carboxamide (**23**) and 1,6-phenazinedimethanol (**24**) (Figure 2) were discovered from the *Brevibacterium* sp. KMD 003 is derived from *Callyspongia* sp. (Kyeongpo, Gangwondo, Korea). The two compounds demonstrated antibacterial activities against *Enterococcus hirae* and *Micrococcus luteus* with an IC_50_ value of 5 μM [9]. JBIR-58 (**25**) (Figure 2), a new salicylamide derivative, was purified from a sponge-associated actinomycetes *Streptomyces* sp. SpD081030ME-02 was collected offshore from Ishigaki Island, Japan. JBIR-58 exhibited cytotoxic activity against human cervical carcinoma HeLa cells with an IC_50_ value of 28 μM [9,25].

A novel indole alkaloid streptomycindole (**S4**) and a known related compound N-phenyl acetyl-_L_-tryptophan (**S5**) were isolated from *Streptomyces* sp. DA22 is associated with *Craniella australiensis* collected at the South China Sea [26]. In 2011, Rong-Bian Wei et al. reported isolation and identification of two new kijanimicin derivatives lobophorins C (**26**) and D (**27**) (Figure 2) from *Streptomyces carnosus* strain AZS17 associated with the marine sponge *Hymeniacidon* sp. collected from coastal waters of the East China Sea. Lobophorin C demonstrated potent cytotoxic activity against the human liver cancer cell line 7402 with IC_50_ values of 0.6 μg/mL and lobophorin D showed a significant inhibitory effect on the growth of human breast cancer cells MDA-MB 435 with an IC_50_ value of 7.5 μM [27].

In 2012, Abdelmohsen et al. reported unprecedented antioxidant and anti-protease activities of a dibenzodiazepine alkaloid diazepinomicin (**28**) (Figure 2) separated from the *Micromonospora* sp. RV115 recovered from the Mediterranean sponge *Aplysina aerophoba*. Additionally, diazepinomicin displayed broad-spectrum antitumor activity and antiparasitic activity against trypomastigote forms of *Trypanosoma brucei* (IC_50_ = 13.5 μM) [9,28].

Compound WS-9659 A (**29**) (Figure 2) separated from *Streptomyces* sp. CMS JV M18_3 associated with marine sponge *Agelas sceptrum* collected in Mona Island exhibited inhibitory activity on testosterone 5α-reductase [3,29]. Three new trichostatin analogs JBIR-109 (**30**), JBIR-110 (**31**), and JBIR-111 (**32**) (Figure 2) were purified together with trichostatin A (**S6**) and trichostatic acid (**S7**) from *Streptomyces* sp. RM72 is associated with an unidentified marine sponge collected near Takara Island, Japan. The compounds **30**–**32** have relatively weak inhibitory effects on histone deacetylase with IC_50_ of 48 μg/mL, 74 μg/mL, and 57 μg/mL, respectively [9,30]. In addition, a new metabolite JBIR-107 (**S8**) was produced by sponge-derived *Streptomyces tateyamensis* NBRC 105047 in 2013 [31]. Four glutarimide-derived compounds (**33** and **S9**–**S11**) were purified from *Streptomyces anulatus* S71 cultivated from a marine sponge *Aplysina aerophoba* collected at the Yongxing Island in the South China Sea. Compound **33** (Figure 2) was identified as a new member of the glutarimide antibiotics family [32].

*Salinispora* sp FS-0034 collected from a sponge *Theonella* sp. produced rifamycin W (**34**) (Figure 2) with antibacterial activity. Rifamycin W showed potent antibacterial activity against multi-drug resistant human pathogens such as methicillin-resistant *Staphylococcus aureus* (MRSA), wild type *Staphylococcus aureus* (WTSA), and vancomycin-resistant *Enterococcus faecium* (VREF) with MICs of 15.62, 7.80 and 250.00 µg/mL, respectively [33].

*Streptomyces* sp. M7_15 associated with the Puerto Rican Sponge *Scopalina ruetzleri* produced an angucyclinone derivative frigocyclinone (**35**) and six new angucyclinone derivatives monacyclinones A–F (**36**–**41**) (Figure 2). Frigocyclinone (**35**) with an unusual C-glycosidic linked aminodeoxy sugar moiety and monacyclinones A–F exhibited biological activity against rhabdomyosarcoma cancer cells (SJCRH30). Monacyclinones A–F also inhibits Gram-positive bacteria. Among them, monacyclinone F (**41**) showed the strongest bioactivity against SJCRH30 (EC_50_ = 0.73 µM) and Gram-positive bacteria. The analysis of their structures and bioactivities suggested that the aminodeoxysugar subunit, the epoxide groups, and the ketone moiety could all be important for biological activity. [34].

Naphthacene glycoside SF2446 A2 (**42**) (Figure 2) was isolated from *Streptomyces* sp. strain RV15 obtained from the sponge *Dysidea tupha* collected offshore Rovinj, Croatia. The SF2446 A2, previously reported against Gram-positive bacteria and several mycoplasma strains, has proven to possess two novel activities. One is the potential of inhibiting *Chlamydia trachomatis* and inhibiting the primary infection and progeny formation in a dose-dependent manner. In addition, it can destroy the surface area of *Schistosoma mansoni* and affect the gonad by impairing the formation of oogenesis and spermatogenesis [35].

In 2015, Xin Zhen et al. explicitly disclosed for the first time that tirandamycin B (**44**) is a 1-keto-4′-enol form, which is different from the 1-enol-4′-keto form of tirandamycin A (**43**) (Figure 2). The two antibiotic tirandamycins A (**43**) and B (**44**), together with staurosporine (**3**), were discovered from *Streptomyces* sp. LS298 is associated with marine sponge *Gelliodes carnosa* [36]. Tirandamycin A has inhibitory activity on bacterial RNA polymerase, and tirandamycin B inhibited the asparaginyl-tRNA synthetase (AsnRS) of *Brugia malayi* with an IC_50_ value of 30 μM [37,38]. In addition, staurosporine showed antifungal activity, protein kinase C inhibitory activity (IC_50_ = 2.7 nM) and great cytotoxic activity against HeLa S3 cells (IC_50_ = 4 × 10^−12^ M) [39].

Two novel metabolites, strepoxazine A (**45**) and ageloline A (**46**), together with two known antibiotic phenazines phencomycin (**47**) and tubermycin B (**48**) (Figure 2), were isolated from *Streptomyces* sp. SBT345 cultivated from Mediterranean sponge *Agelas oroides*. Strepoxazine A (**45**) exhibited a significant cytotoxic effect against human promyelocytic leukemia cells HL-60 with IC_50_ at 8 μg/mL. Ageloline A showed antioxidant potential and further reduce oxidative stress and genomic damage induced by 4-nitroquinoline-1-oxide (NQO). In addition, ageloline A also showed antichlamydial activity by inhibiting the formation and growth of *Chlamydia trachomatis* inclusion in a dose-dependent manner with an IC_50_ value of 9.54 ± 0.36 μM [40,41]. 

In 2016, Sekurova et al. reported isolation and identification of four new deferoxamine analogs (**49**, **S12**–**S14**) with additional acyl and sugar moieties from upon overexpression of Pathway-Specific Regulatory Gene in the *Streptomyces albus* PVA94-07 associated with sponge collected in the Trondheim fjord (Norway). Compound **49** (Figure 2) showed 52–56% inhibition of *E. coli* at 16 µg/mL [42]. Comparing the structure and activity of deferoxamine analogs **49** and **S14**, it was found that the position of the hydroxyl group in the enamine group affected the antibacterial activity.

A new azepino-diindole alkaloid rhodozepinone (**50**) (Figure 2), along with two known compounds 2-amino-3-[2(1H)-quinolinon-4-yl]propionic acid (**S15**) and indole-3-acetic acid (**S16**), was isolated from *Rhodococcus* sp. UA13 obtained from the Red Sea sponge *Callyspongia aff*. implexa. Rhodozepinone (**50**) exhibited significant antibacterial and antitrypanosomal activities against *Staphylococcus aureus* NCTC 8325 (IC_50_ = 8.9 µg/mL) and *Trypanosoma brucei brucei* TC221 [IC_50_ = 16.3 (48 h) and 11.8 (72 h) µg/mL], respectively [43]. 

Jian Lin Li et al. separated three dimeric indole derivatives (**51**–**53**) (Figure 2) from an actinomycete strain *Rubrobacter radiotolerans* cultivated from sponge *Petrosia* sp. obtained off the coast of Xisha Islands, China. All three metabolites suppressed chlamydial growth in a concentration-dependent manner. Among them, the novel one (**51**) exhibited the most effective antichlamydial activity with IC_50_ values of 46.6–96.4 µM in the production of infectious progeny [44].

2,3-dihydroxybenzamide (**54**) (Figure 2) was isolated from *Streptomyces* sp. SBT348 associated with *Petrosia ficiformis* from Milos, Greece. It exhibited significant cytotoxicity against human promyelocytic HL-60 and human colon adenocarcinoma HT-29 cell lines [45].

Isolation of three new anthranilic acid derivatives anthranosides A−C (**S17**, **S18**, **55**) were reported in 2018 from the culture of sponge-derived *Streptomyces* sp. CMN-62 was collected at NaoZhou Island of the Guangdong Province, China. Anthranoside C (**55**, Figure 2) with a unique indole-containing scaffold showed anti-influenza H1N1 activity with an IC_50_ =171 μM (ribavirin as positive control, IC_50_ 133 μM) [46].

Saccharomonosporine A (**56**) (Figure 2) and a novel brominated oxo-indole alkaloidconvolutamydine F (**S19**), along with other two known induced metabolites (**57**, **S20**), were identified from the co-culture of *Saccharomonospora* sp. UR22 and *Dietzia* sp. UR66, cultivated from the Red Sea sponge *Callyspongia siphonella*. Additionally, the axenic culture of *Saccharomonospora* sp. UR22 led to the isolation of four known microbial metabolites **S21**–**S23** and **58** (Figure 2). Compounds **56** and **57** (Figure 2) were potent Pim-1 kinase inhibitors and displayed significant antiproliferative activities against HT-29 and HL-60 cell lines [47].

Tetrocarcin Q (**59**) is a novel spirotetronate glycoside with a unique glycosyl group 2-deoxy-allose at the C-9 position, which was separated together with six known analogs tetrocarcin A (**60**), AC6H (**61**), tetrocarcin N (**62**), tetrocarcin H (**63**), arisostatin A (**64**) (Figure 2), and tetrocarcin F1 (**S24**) from *Micromonospora carbonacea* LS276 (FJ937935) associated with *Gelliodes carnosa* from Ling Shui Bay. Compound **59** displayed moderate antibacterial activity against *Bacillus subitlis* ATCC 63501 with a MIC value of 12.5 µM. Compounds **60**–**64** also showed potent antibacterial activity against *Bacillus subitlis* ATCC 63,501 with MICs of <0.048, 0.5, 1.562, 50, 0.048 µM, respectively. In addition, compounds **60** and **64** showed moderate activity against four cell lines (A549, BGC823, HCT116, HepG2) with the IC_50_ values ranging from 5.33 µM to 19.7 µM, and also exhibited the most potent antitumor activity against U87MG cell line with IC_50_ values of 0.50 µM and 2.42 µM, respectively. On comparing the structure and activity of analogs (**60**–**64**), it was found that the modification of the tetronolide skeleton affected the in vitro antitumor activity to some extent. Tetrocarcin F1 (**S24**) was inactive, indicating that the sugar moiety at C-9 position could play an important role in the antitumor activity. In addition, compound **59** showed no or weak in vitro antitumor activity, suggesting that the deoxy sugar analog may also influence the antitumor activity [48].

Tirandamycins A and B (**43**, **44**) were isolated from strain HNM0039^T^, a novel *Streptomyces* sp. named *Streptomyces tirandamycinicus* sp. nov., which was obtained from a marine sponge collected from the coast of Wenchang, Hainan Province of China. Tirandamycins A and B displayed potent inhibitory activity against *Streptococcus agalactiae* HNe0 and showed antibacterial activity against *Bacillus subtilis* GIM1.222 [49].

2-ethylhexyl-1H-imidazole-4-carboxylate (**65**) (Figure 2) and a known alkaloid butyl 1H-imidazole-4-carboxylate (**S25**), were isolated from *Verrucosispora* sp. FIM06-0036, associated with marine sponge sample from the East China Sea. Compound **65** was active against *H. pylori, K. pneumonia, S. aureus*, and *E. faecium* with MIC values of 8 µg/mL, 64µg/mL, 16 µg/mL, and 256 µg/mL, respectively [50]. The *Verrucosispora* sp. FIM06025 obtained from a sponge sample collected from the East China Sea, led to the isolation of two new alkaloids (**66** and **S26**). Among them, compound **66** (Figure 2) exhibited a broad spectrum of antimicrobial activity with MIC values ranging from 3.4 to 200 μg/mL [51]. Fridamycin I (**S27**) was first isolated from sponge-derived *Actinokineospora spheciospongiae* sp. nov., obtained from the Red Sea sponge *Spheciospongia vagabunda* collected from offshore Ras Mohamed, Egypt [52].

In 2018, a sponge-derived strain MCCB267 was obtained from *Mycale* sp., collected in the Indian Ocean off the southeast coast of India. The strain MCCB267 designated as *S. zhaozhouensis* subsp. *mycale*. subsp. nov. led to the discovery of four the polycyclic tetramate macrolactam (PTM) family: ikarugamycin (IK) (**67**), clifednamide A (CF) (**68**), 30-oxo-28-N-methylikarugamycin (OI) (**69**), and 28-N-methylikarugamycin (MI) (**70**) (Figure 2). The four compounds **67**–**70** were active against NCI-H460 lung carcinoma cells in vitro, by inducing apoptosis. Compounds **67**, **69**, and **70** induced cell cycle arrest during the G1 phase in the NCI-H460 cell line, whereas **68** induced cell arrest in the S phase [53].

Borrelindine J and K (**71** and **72**) (Figure 2) with rare nitrile group were obtained only in the co-culture of sponge-associated *Streptomyces rochei* MB037 with fungus *Rhinocladiella similis* 35 derived from gorgonian, along with two known 18-membered macrolides, borrelidin (**S28**), and borrelidin F (**S29**). Compounds **71** and **72** showed potent antibacterial activity against methicillin-resistant *Staphylococcus aureus* with MIC values of 0.195 and 1.563 µg/mL, respectively [54].

Isolation of 9H-pyrido[3,4-b]indole (**S30**) and 9H-purin-6-amine (**S31**) was reported in 2019 from sponge-derived *Streptomyces* sp. G248 collected in the East Vietnam Sea [55]. In addition, the 9H-pyrido[3,4-b]indole (**S30**) was identified from sponge-associated *Streptomyces* sp. G246 collected at Son Tra island (Da Nang)-Vietnam. Along with 9H-pyrido[3,4-b]indole, another known compound indole-3-acetic acid (**S16**) was discovered from this strain [56].

Two quinocycline antibiotics strains, quinocycline B (kosinostatin) (**73**) and isoquinocycline B (**74**) (Figure 2), were separated from the strain 28ISP2-46^T^ assigned to a novel species *Micromonospora ferruginea* sp. which was recovered from a mid-Atlantic deep-sea sponge. Compounds **73** and **74** showed antibiotic activity and also inhibited DNA topoisomerase IIα. Isoquinocycline B (**74**) showed good antibiotic activity not only against the Gram-positive strain *S. aureus* SH1000, but also the Gram-negative strains efflux knockout (KO) mutant strains of *K. pneumoniae* ATCC10031 and *A. baumannii* ATCC 17978. It was also active against the human liver cancer cell line HepG2 with an IC_50_ of 13.3 µM [57].

*Streptomyces* sp. RM66 derived from the Red Sea sponge *Amphimedon* sp. in Hurghada (Egypt), led to the discovery of eight phenazine analogs (**S32**–**S37**, **47**, **48**) through the addition of GlcNAc. Tubermycin B (**48**) showed environmentally compatible antimicrobial activity against various phytopathogens. Phencomycin (**47**) exhibited antibacterial activities against E*scherichia coli* and *Bacillus subtilis* [58].

Marine sponge *Callyspongia* sp. collected from Hurghada (Red Sea, Egypt) led to the isolation of two actinobacteria *Rhodococcus* sp. UR59 and *Actinokineospora spheciospongiae* strain EG49. In addition, the strain EG49 was previously discovered the Red Sea sponge *Spheciospongia vagabunda*. UK-2B (**75**) with antifungal activity was isolated from the axenic cultures of strain EG49. And the separate cultivation of strain UR59 leads to the identification of Mitomycin K (antitumor activity) (**76**), Piericidin F (anticancer activity) (**77**), Migrastatin (anticancer activity) (**78**) (Figure 2) [59].

##### Alkaloids Derived from the Coral-Associated Actinomycetes

**Figure 3:**
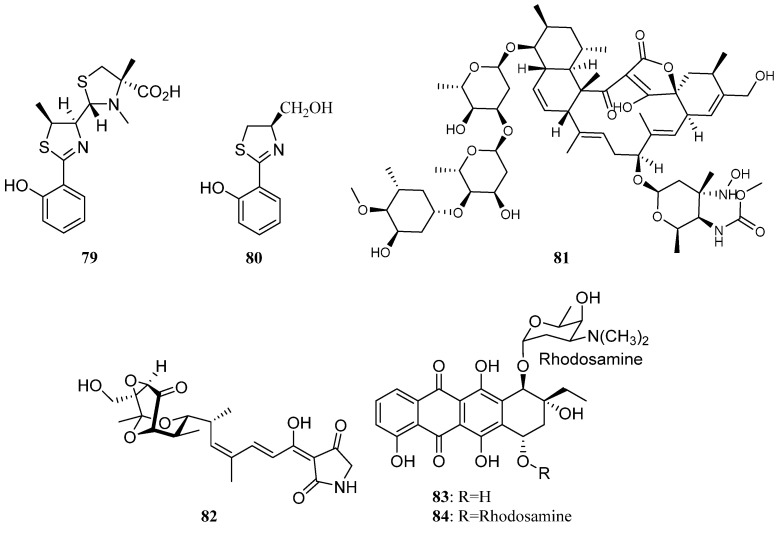
Structures of compounds **79**–**84**.

Three thiazole derivatives known as watasemycin A (**79**) (Figure 3), pulicatin G (**S38**), and aerugine (**80**) (Figure 3) were identified together with pyrrole-2-carboxamide (**S39**) and furan-2-carboxamide (**S40)** from *Streptomyces* sp. OUCMDZ-1703 associated with a soft coral sample collected from the South China Sea. Thiazole derivatives **79** and **80** displayed moderate antibacterial activity against *S. aureus* and three methicillin-resistant strains MRSA082, MRSA111, and MRSA234 [60].

Lobophorin K (**81**) (Figure 3), a novel metabolites separated from *Streptomyces* sp. M-207 associated with *Lophelia pertusa* collected at submarine Avilés Canyon exhibited cytotoxicity against two human tumor cell lines pancreatic carcinoma (MiaPaca-2) and breast adenocarcinoma (MCF-7). It also showed moderate and selective antibiotic activity against pathogenic Gram-positive bacteria such as *Staphylococcus aureus* [61].

Ziwen Cong et al. reported the isolation of isotirandamycin B (**82**) (Figure 3) from *Streptomyces* sp. SCSIO 41399 cultivated from the *Porites* sp. coral collected in Wenchang, Hainan province of China. Along with isotirandamycin B, four other known compounds anthracycline derivatives (**83**, **84**) (Figure 3) and tirandamycin derivatives (**43** and **44**) were also discovered from this strain**.** Compounds **82**, **43**, and **44** were active against *Streptococcus agalactiae* with MIC values of 11.5, 5.9, and 5.7 μM, respectively. Besides, compounds **83** and **84** displayed moderate in vitro cytotoxicity against the K562 cell lines with IC_50_ values of 1.80 ± 0.01 and 12.1 ± 0.07 μM, respectively [62].

Two novel metabolites uridine derivative 11457A (**S42**) and indole derivative 11457B (**S43**), together with 1H-indole-2-carbal-dehyde (**S44**) were isolated from *Pseudonocardia* sp. SCSIO 11457, which had been cultivated from the scleractinian coral *Galaxea fascicularis* [63].

##### Alkaloids Derived from the Ascidian-Associated Actinomycetes

**Figure 4:**
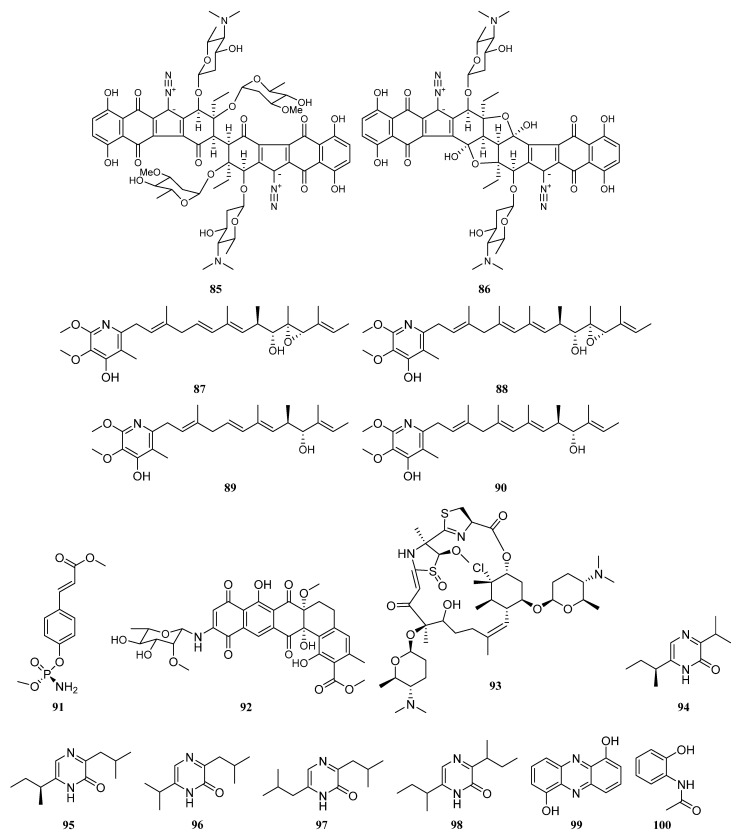
Structures of compounds **85**–**100**.

Two novel antitumor antibiotics, lomaiviticins A (**85**) and B (**86**) (Figure 4) with unique dimeric diazobenzofluorene glycosides were discovered from *Salinispora pacifica* LL-37I366 derived from marine ascidian *Polysyncraton lithostrotum*. Lomaiviticins A and B were demonstrated to be significant DNA-damaging agents with a minimum induction concentration ≤0.1 ng/spot. They also exhibited potent antimicrobial activities against Gram-positive bacteria *Staphylococcus aureus* and *Enterococcus faecium* (MICs, 6–25 ng/spot). In addition, lomaiviticin A also displayed cytotoxicity against a number of cancer cell lines with IC_50_ values ranging from 0.01 to 98 ng/mL [64,65,66].

A novel dibenzodiazepine alkaloid diazepinomicin (**28**) was obtained from *Micromonospora* sp. DPJ12 separated from marine ascidian *Didemnum proliferum* Kott which was collected at Shishijima Island, Japan. Diazepinomicin showed modest antimicrobial activity against selected Gram-positive bacteria with MIC of approximately 32 µg/mL [67].

Two new piericidin compounds C7 (**87**) and C8 (**88**), together with previously identified piericidins A1 (**89**) and A2 (**90**) (Figure 4) were isolated from the *Streptomyces* sp. YM14-060, which is associated with an unidentified greenish ascidian from Iwayama Bay, Palau. The compounds **87**–**90** exhibited cytotoxicity against RG-E1A-7 rat glial cells and inhibited the growth of Neuro-2a mouse neuroblastoma cells [64,68]. *Streptomyces* sp. JP90 separated from marine ascidian *Aplidium lenticulum* (Great Barrier Reef, Australia) produced a new organophosphate (*S*)-cinnamoyl-phosphoramide (**91**) (Figure 4), which displayed inhibitory activity towards Butyrylcholinesterase (BChE) [64].

Isolation of a new antibiotic arenimycin (**92**) (Figure 4) was reported in 2009 from *Salinispora arenicola* strain CNR-647 associated with sea squirt *Ecteinascidia turbinate* collected at Sweetings Cay, Grand Bahama Island. Arenimycin exhibited potent activities against drug-resistant *Staphylococci*, some other Gram-positive microanimals and plants, and one *Mycobacterium* strain. In addition, arenimycin was also active against eukaryotic cell division, which may lead to non-selective cytotoxicity [64,69].

The marine bacterium *Aeromicrobium halocynthiae* KME 001^T^, which was separated from sea squirt *Halocynthia roretzi* (Gangneung, Korea), led to the discovery of taurocholic acid (**S45**) [64,70]. Bohemamine (**S46**) was isolated from *Streptomyces* sp., a marine actinomycete associated with an unidentified ascidian collected from Lyttelton Harbor, New Zealand [64]. Forazolines A (**93**) (Figure 4) and B (**S47**) were isolated from *Actinomadura* sp. WMMB-499 that was obtained from the marine ascidian *Ecteinascidia turbinate*. Forazoline A (**93**) displayed inhibitory activity against the fungal pathogen *Candida albicans* with a MIC value of 16 μg/mL [71].

Three new 2(1*H*)-pyrazinone derivatives including (*S*)-6-(sec-butyl)-3- isopropylpyrazin-2(1*H*)-one (**94**) (Figure 4), (*S*)-3-(sec-butyl)-6-isopropylpyrazin-2(1*H*)-one (**S48**) and (*S*)-6-(sec-butyl)-3-isobutylpyrazin-2(1*H*)-one (**95**), together with the known (1*H*)-pyrazinones analogues deoxymutaaspergillic acid (**96**), 3,6-diisobutyl-2(1*H*)- pyrazinone (**97**) and 3,6-disec-butyl-2(1*H*)-pyrazinone (**98**) (Figure 4), were isolated from *Streptomyces* sp. Did-27, which is associated with the marine ascidian *Didemnum* sp. Expect for compound **S48**, all other compounds presented cytotoxic activities against cancer cell lines HCT-116, HepG2 and MCF-7 [64,72].

Compound 1, 6-dihydroxyphenazine (**99**) (Figure 4) was produced by *Nocardiopsis dassonvillei* HQA404 derived from star ascidian *Botryllus schlosseri*. This phenazine (**99**) has antimicrobial activity against *Vibrio anguillarum* and *Vibrio parahaemolyticus*, lethal activity against *Artemia salina*, and enzyme inhibiting activity against Alpha-glucosidase. 2-(acetylamino)-phenol (**100**) (Figure 4) isolated from *Nocardiopsis dassonvillei* HQA404 showed lethality against brine shrimp *Artemia salina* [64].

##### Alkaloids Derived from the Actinomycetes Associated with Other Marine Animals

**Figure 5:**
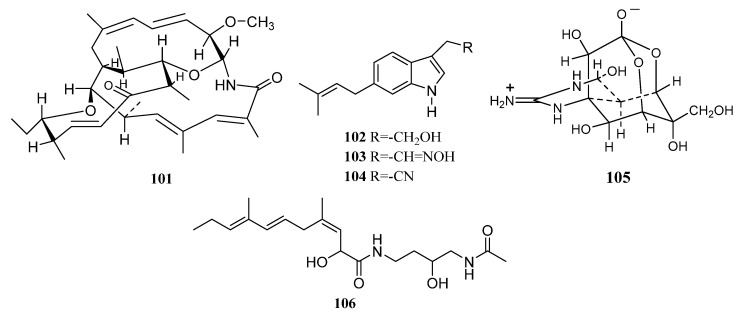

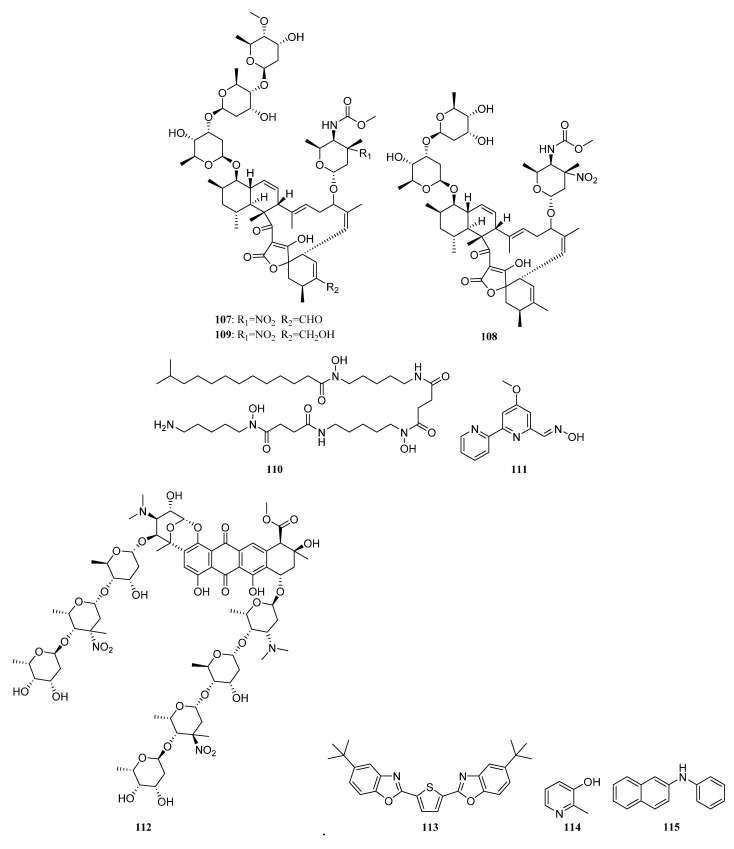

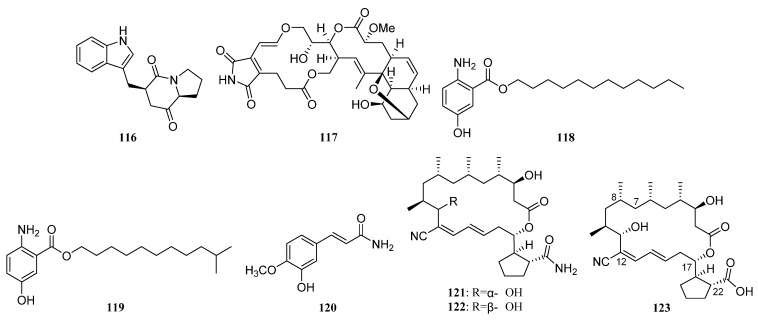
Structures of compounds **101**–**123**.

Halichomycin (**101**) (Figure 5) was a novel structurally unique macrolide with potent cytotoxicity, separated from *Streptomyces hygroscopicus* OUPS-N92 obtained from the gastrointestinal tract of marine fish *Halichoeres bleekeri*. Halichomycin exhibited significant cytotoxicity in the P-388 lymphocytic leukemia test system with ED_50_ of 0.13 μg/mL [73].

Isolation of three novel cytotoxic indolic metabolites 3,6-disubstituted indoles (**102**–**104**) (Figure 5) were reported in 2003 from *Streptomyces* sp. BL-49-58-005, which was isolated from an unidentified Mexican marine invertebrate. Indole A (6-prenyltryptophol) (**102**) exhibited the best activity against K-562 (leukemia) with a GI_50_ value of 8.46 µM. Aldoxime indole B (**103**) showed activity with GI_50_ values within the micromolar range against different cancer cell lines [74].

*Nocardiopsis dassonvillei* RG-33B isolated from the ovary of pufferfish *Fugu rubripes* from the Bohai Sea of China produced tetrodotoxin (**105**) (Figure 5). Tetrodotoxin (TTX) is one of the most potent nonprotein neurotoxins [75]. Two known inactive metabolites bohemamine (**S46**) and bohemamine B (**S49**) were identified from the small-scale extract of *Streptomyces* sp. (LA3L2) derived from New Zealand and Malaysian marine invertebrates. Additionally, thiazostatin B (**S50**) was produced by *Streptomyces* sp. (LA5L4) [76].

A new metabolite JBIR-66 (**106**) (Figure 5) isolated from *Saccharopolyspora* sp. strain SS081219 JE-28 associated with an unidentified tunicate collected at the seashore of Tateyama City, Chiba Prefecture, Japan, displayed relatively weak activity against human lymphoblastoid Namalwa cells [64,77].

*Streptomyces* sp. 1053U.I.1a.3b, cultivated from *Lienardia totopotens*, a new species of conoidean mollusk collected in the Philippines, led to the identification of two novel lobophorins H-I (**S51**, **107**) and three known lobophorins F (**108**), B (**109**), C (**26**). Compounds **107-109** (Figure 5) and **26** showed strong inhibitory activity to *M. tuberculosis* and *B. subtilis* with MIC values ranging from 1.3 to 24 μM. In addition, all these active compounds also showed strong cytotoxicity against the human CEM-TART cancer cell line [78].

A novel hydroxamate metabolite MBJ-0003 (**110**) (Figure 5) was produced by *Micromonospora* sp. 29867 derived from a shellfish collected in Suruga Bay, Shizuoka Prefecture, Japan. MBJ-0003 displayed moderate cytotoxicity against human ovarian adenocarcinoma SKOV-3 cells with the IC_50_ values of 11 μM [79].

Caerulomycin A (**111**) (Figure 5), which was extracted from the *Actinoalloteichus* sp. strain PM0525875 associated with a marine invertebrate collected from the deep-sea (Anjuna Beach, Goa, India), showed potent in vitro antifungal activity against drug-resistant fungal strains and its minimum inhibitory concentration (MIC) was found in the range of 0.39–1.56 µg/mL against pathogenic fungal test strains [80].

Keyicin (**112**) (Figure 5), a novel and otherwise unattainable bisnitroglycosylated anthracycline antibiotic, was discovered from the producer *Micromonospora* sp. co-culturing with *Rhodococcus* sp. associated with marine invertebrates. The Keyicin was selectively active against Gram-positive bacteria including *Rhodococcus* sp. and *Mycobacterium* sp. *E. coli*-based chemical genomics studies revealed that keyicin’s MOA, in contrast to many other anthracyclines, does not invoke nucleic acid damage [81].

Compounds **58**, **113**–**116** (Figure 5), **S21,** and **S52** were isolated from *Streptomyces* sp. G278 that was obtained from echinoderm *Holothuria edulis* collected in Cu Lao Cham–Quang Nam. Compounds **113** and **114** were isolated from a natural source for the first time. Compounds **113** selectively inhibited *Enterococcus faecalis*. Compound **113** was proven to have antibacterial and antifungal activity and the known metabolites (**115**, **116**, **58**) exhibited antimicrobial activity. In addition, compound **115** possessed antifouling activities [82].

The isolation of a new pyrazolidine derivative, 1-acetyl-2-isobutyrylpyrazolidine-4- carboxylic acid (**S53**) was reported in 2018 from a sea anemone-associated actinomycete *Streptomyces* sp. ZZ406 separated from *Haliplanella lineate* [83]. An unusual macrodilactone streptoseomycin (**117**) (Figure 5) with potent bioactivity against *Helicobacter pylori* with a MIC value of 2 μg/mL was discovered from *Streptomyces seoulensis* A01 associated with a marine prawn collected in the Yellow Sea in China. It also exhibited antibacterial activities against a panel of microaerophilic bacteria with MICs in the range of 4–8 μg/mL [84].

Five novel 5-hydroxyanthranilic acid derivatives anthocidins A-D (**S54**–**S57**) and crassilin (**S58**) were purified from a sea urchin-derived actinomycete *Streptomyces* sp. HDa1, which was obtained from the gut of *Anthocidaris crassispina* collected from Hainan Island, China. Additionally, two known analogs n-lauryl 5-hydroxyanthranilate (**118**) and isolauryl 5-hydroxyanthranilate (**119**) (Figure 5), along with benzamide (**S59**), 3-hydroxy-4-methoxycinnamamide (**120**) (Figure 5), and oxachelin (**S60**) were also discovered from this strain. Compounds **118** and **119** were demonstrated to possess potent in vitro 5-lipoxygenase inhibitory activity, and herein, compound **118** was isolated and reported as a natural product for the first time. Compound **120** showed weak activity against the Gram-positive bacterium *Bacillus subtilis* with an inhibition zone of 3 mm [85,86].

In 2020, Zhenbin Zhou et al. reported isolation and identification of three novel borrelidins M-O (**121**, **S61**, **S62**), together with four previously known borrelidins CR1, A, E, and K (**122**, **123**, **S63**, **S64**) from *Streptomyces olivaceus* SCSIO LO13 associated with pulmonated mollusks *Onchidium* sp. collected at Daya Bay, South China Sea. Borrelidin A (**123**) (Figure 5) has a variety of biological activities such as antibacterial, anti-parasite, and cytotoxic activities. It showed significant activity against *Micrococcus luteus* with an MIC value of <0.5 μM. Borrelidins M (**121**) and CR1 (**122**) (Figure 5) displayed weak cytotoxicity against normal human hepatic cell line L02, but no inhibitory effect on cancer cell lines was detected. In addition, borrelidins M, CR1, and K exhibited moderate activity against *Micrococcus luteus* with MIC values of 33 μM [87]. The analysis of structure-activity relationships revealed that the carboxyl moiety at C-22 and the position of hydroxylation are significant for both cytotoxic and antibacterial activities. In addition, the cytotoxicities of borrelidins were correlated to the cyano moiety, stereo configurations of the hydroxyl moiety at C-11, and the C-C double bonds. And the steric structural arrangement within the C-17 side chain is important for differentiating cytotoxic and antiangiogenic activities [87,88,89].

#### 3.1.2. Polyketides

Polyketides are a large class of secondary metabolites produced by bacteria, fungi, actinomycetes, or plants. They are synthesized by simple fatty acids under the catalysis of polyketide biosynthase (PKS) through a synthesis pathway similar to long-chain fatty acids. Its structural types include macrolides, aromatic polyketides, polyether, pyranones, and other polyketides.

##### Polyketides Derived from the Sponge-Associated Actinomycetes

**Figure 6:**
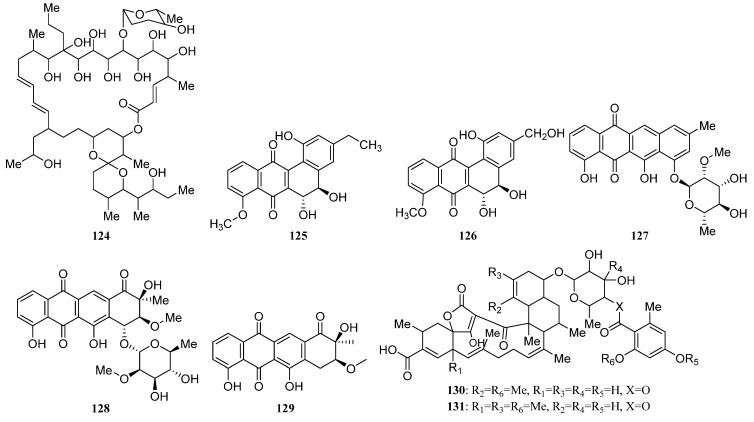

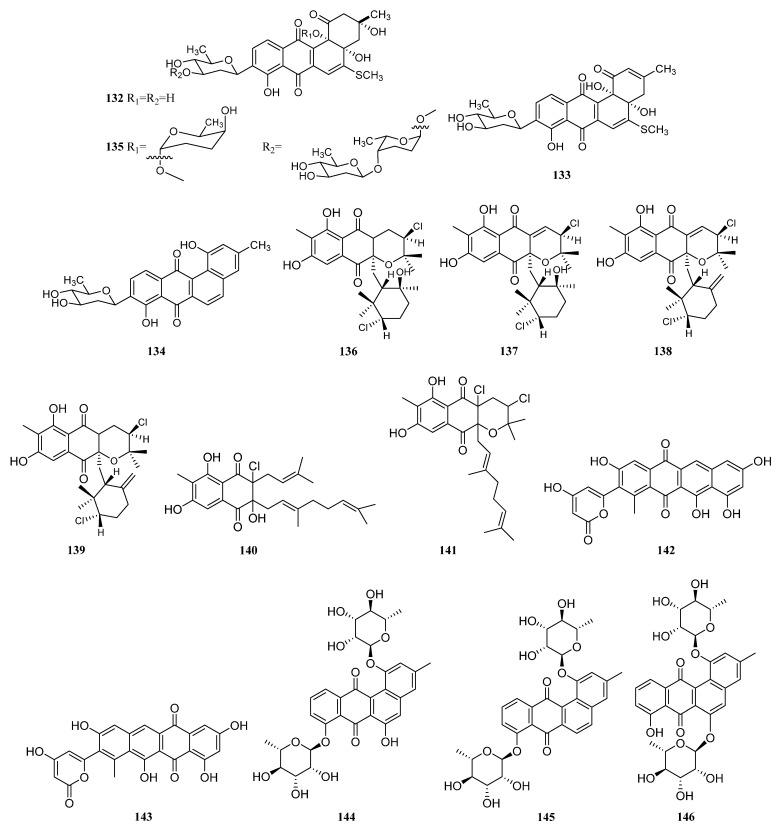

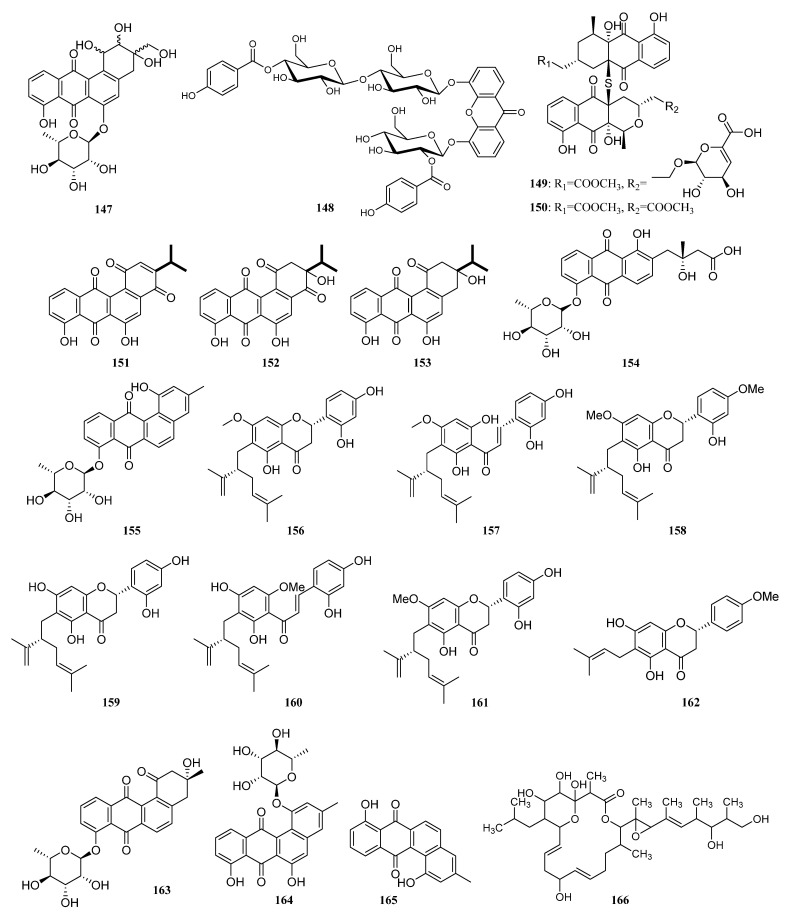
Structures of compounds **124**–**166**.

IB-96212 (**124**) (Figure 6) is a 26-membered spiroketal macrolide produced by *Micromonospora* sp. L-25-ES25-008 was obtained from an unidentified sponge from the Indian Ocean, Mozambique. It exhibited cytotoxic activity against mouse leukemia P-388, human lung non-small carcinoma A-549, colon adenocarcinoma HT-29, and melanoma MEL-28 cell lines [9,90].

The identification of a new angucyclinone PM070747 (**125**) (Figure 6) was reported from sponge-derived *Saccharopolyspora taberi* PEM-06-F23-019B obtained from near the coast of Tanzania, together with the known angucyclinone PD116740 (**126**) (Figure 6). The two angucyclinones showed antitumor activity and compound **126** was active against leukemia and adenocarcinoma cell lines [91].

Tetracenoquinocin (**127**) (Figure 6) was separated from sponge-associated *Streptomyces* sp. Sp080513GE-26 and exhibited cytotoxicity against human cervical carcinoma HeLa cells and human acute myelogenous leukemia LH-60 cells with IC_50_ values of 2.7 μM. Additionally, aranciamycin (**128**) and antibiotic SM 173B (**129**) (Figure 6) were also discovered from strain Sp080513GE-26. Aranciamycin was active against human cervical carcinoma HeLa cells and human acute myelogenous leukemia LH-60 cells with IC_50_ values of 2.7 and 4.1 μM, respectively. On comparing the cytotoxic activity of tetracenoquinocin (**127**), 5-iminoaranciamycin (**S3**), and aranciamycin (**128**), it was found that the ketone functional group at C-5 is essential for the cytotoxicity against the cancer cells [9,22].

Four new γ-pyrones nocapyrones A–D (**S65**–**S68**) were purified from the *Nocardiopsis* strain HB383 associated with *Halichondria panacea* collected from the Baltic Sea (Germany) [9,92]. The new tetronic acid derivatives tetromycins 1-4 (**S69**, **S70**, **130**, **131**) and a known one tetromycin B (**S71**) were produced by *Streptomyces axinellae* Pol001^T^, which was isolated from the Mediterranean sponge *Axinella polypoides*. Tetromycins 3 and 4 (**130**, **131**) (Figure 6) displayed protease inhibition activities against several cysteine proteases and exhibited pronounced activity against Gram-positive bacteria methicillin-resistant *Staphylococcus aureus* [9,93].

Three new C-glycosylated benz[a]anthraquinone derivatives, urdamycinone E (**132**), urdamycinone G (**133**), and dehydroxyaquayamycin (**134**) (Figure 6) were identified from *Streptomycetes* sp. BCC45596 separated from marine sponge *Xestospongia* sp. collected at Sichang Island, Chonburi, Thailand. Urdamycin E (**135**) (Figure 6), the possible biosynthetic precursor of **132**–**134**, has also been identified from this strain. These compounds (**132**–**135**) exhibited potent anti-*Plasmodium palcifarum* K1 strain with IC_50_ values of 0.0534–2.93 μg/mL and anti-*Mycobacterium tuberculosis* with minimum inhibition concentrations (MICs) in a range of 3.13–12.50 μg/mL [94,95].

Six dihydroquinone derivatives (**136**–**141**) (Figure 6) were isolated from sponge-associated *Streptomyces* sp. CMS JV M18_3 [3]. Chloro-Dihydroquinone 1–4 (**136**–**139**) has great antibacterial activities against MRSA and vancomycin-resistant *Enterococcus faecium* (VREF). In addition, compounds **136**–**139** were active against HCT-116 human colon carcinoma [96]. SF2415B3 (**141**) displayed anti-biofilm activity inhibiting *Staphylococcus aureus* biofilm formation, and compound **140** exhibited antimicrobial activities against some Gram-positive bacteria [97,98].

In 2014, Min Cheol Kim et al. reported isolation and identification of two novel tetracenedione derivatives nocatriones A (**142**) and B (**143**) (Figure 6) from *Nocardiopsis* sp. KMF-002 was cultivated from an unidentified dark purple marine sponge. Nocatrione A (**142**) showed a significant protective effect against UVB irradiation in both NHDF cell lines, whereas nocatrione B (**143**) was active against UVB only in a specific NHDF cell line [99].

*Actinokineospora* sp. EG49 cultivated from the marine sponge *Spheciospongia vagabunda* afforded two new actinosporin analogs actinosporins C (**144**) and D (**145**) (Figure 6). At 1.25 μM, actinosporins C and D showed a significant antioxidant and protective capacity from the genomic damage induced by hydrogen peroxide in the human promyelocytic (HL-60) cell line. Additionally, two other new antitrypanosomal angucycline-type metabolites actinosporins A (**146**) and B (**147**) (Figure 6) were also discovered from strain EG49 [100,101].

Microluside A (**148**) (Figure 6) is a unique O-glycosylated disubstituted xanthone separated from *Micrococcus* sp. EG45 obtained from the Red Sea sponge *Spheciospongia vagabunda*. It exhibited antibacterial potential against *Enterococcus faecalis* JH212 and *Staphylococcus aureus* NCTC 8325 with MIC values of 10 and 13 μM, respectively [102]. In 2015, a sponge-derived strain *Streptomyces* sp. M7_15 led to the isolation of dimethyldehydrorabelomycin (**S72**) [34].

Three S-bridged pyranonaphthoquinone dimers naquihexcins A (**149**) (Figure 6) and B (**S73**), and a related analog (−)-BE-52440A (**150**) (Figure 6) were produced by sponge-associated *Streptomyces* sp. HDN-10-293. Among them, (−)-BE-52440A (**150**) showed cytotoxicity against NB4 and HL-60 cells with IC_50_ values of 1.7 and 1.8 μM, respectively. Naquihexcin A (**149**) bears a rare unsaturated hexuronic acid moiety and could inhibit the proliferation of an adriamycin-resistant human breast cancer cell line MCF-7 ADM with IC_50_ = 16.1 μM, indicating that the unsaturated hexuronic acid moiety could enhance the activity against the cancer cells [103].

Compound **S74**, identified as 3-hydroxy-2-methyl-4H-pyran-4-one (maltol), was isolated both from the *Streptomyces* sp. SBT348 and *Rhodococcus* sp. UA13 [43,45]. Compound **S75** was discovered from the co-culture of sponge-derived *Saccharomonospora* sp. UR22 and *Dietzia* sp. UR66 [47]. In 2018, Dongbo Xu et al. reported the discovery of three novel angucyclines nocardiopsistins A-C (**151**–**153**) (Figure 6) separated from *Nocardiopsis* sp. HB-J378 associated with *Theonella* sp. Nocardiopsistin B showed the best anti-MRSA activity with the same MIC (3.12 μg/mL) as that of chloramphenicol, whereas nocardiopsistins A and C have a moderate anti-MRSA activity (MIC = 12.5 μg/mL) [104]. On comparing the anti-MRSA activity of nocardiopsistins A–C (**151**–**153**), it was found that the ketone functional group at C-4 could enhance the anti-MRSA activity, while the hydroxyl group at C-3 weakened activity.

7-methoxy-2,3-dimethylchromone-4-one (**S76**) was first isolated from *Streptomyces rochei* MB037 associated with marine sponge *Dysidea arenaria* collected at Yongxin Island in the South China Sea. And the co-culture with fungus *Rhinocladiella similis* 35 derived from gorgonian could enhance its production [54]. The new fridamycins H (**154**), together with three known actinosporins C, D, and G (**144**, **145**, **155**) (Figure 6) were obtained from sponge-associated *Actinokineospora spheciospongiae* sp. nov. Among them, fridamycin H (**154**) exhibited potent antitrypanosomal activity and growth inhibitory activity towards *Trypanosoma brucei* strain TC221 [52].

Two new lavandulylated flavonoids (**156** and **157**) (Figure 6) were produced by the marine sponge-derived *Streptomyces* sp. G246 had a broad spectrum of antimicrobial activity [56]. Three novel lavandulylated flavonoids **158**–**160** (Figure 6) were isolated along with two known compounds **161** and **162**, from the culture broth of sponge-associated *Streptomyces* sp. G248. Compounds **158**–**160** exhibited remarkable antimicrobial activity. Additionally, two known compounds **161** and **162** (Figure 6) showed inhibitory activity against *Mycobacterium tuberculosis* H37Rv with MIC values of 6.0 µg/mL and 11.1 µg/mL, respectively [55].

Actinosporins A and C (**146**, **144**) were isolated from the axenic cultures of strain EG49. Actinosporin A showed anti-trypanosomal activity and actinosporin C exhibited antioxidant activity. New actinosporins E–H (**163**, **S77**, **155**, **164**) (Figure 6) were produced by EG49 through the activation of cryptic gene cluster by N-acetyl-D-glucosamine (GluNAc). In addition, tetrangulol (**165**) (Figure 6) and the same actinosporins E, G, and H were discovered with antimalarial activity from the co-culture of strains *Actinokineospora spheciospongiae* EG49 and *Rhodococcus* sp. UR59. Tetrangulol was also reported as an antibiotic in previous research. What’s more, the separate cultivation of strain UR59 leads to the isolation of Kaimonolide B (**166**) (Figure 6) (plant growth inhibitor) and 8,15-Dideoxylankanolide (**S78**) [59].

##### Polyketides Derived from the Coral-Associated Actinomycetes

**Figure 7:**
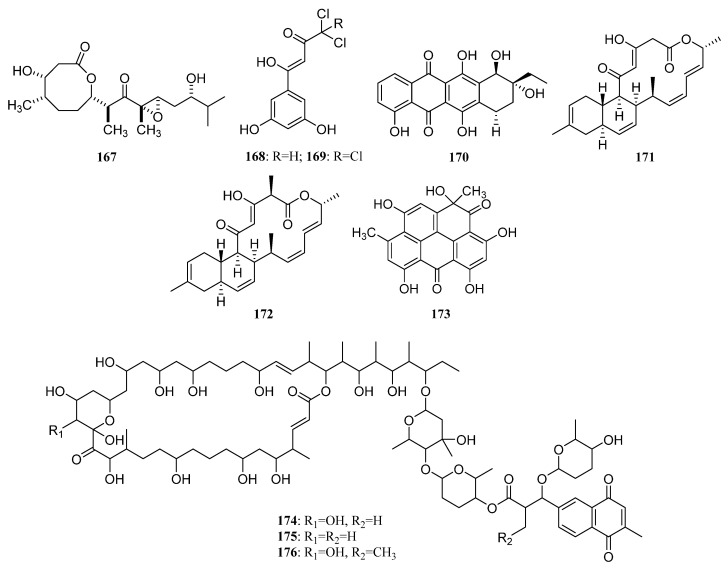
Structures of compounds **167**–**176**.

Isolation of two novel compounds Octalactins A (**167**) (Figure 7) and B (**S79**) with fully saturated eight-membered lactone ring were reported in 1991 from *Streptomyces* sp. PG-19 collected on the surface of Cortez gorgonian octocoral *Pacifigorgia* sp. Octalactin A demonstrated significant in vitro cytotoxicity toward B-16-F10 murine melanoma (IC_50_ = 7.2 × 10^−3^ µg/mL) and HCT-116 human colon tumor (IC_50_ = 0.5 µg/mL) [105].

A novel analog of jadomycin B, 7b,13-dihydro-7-O-methyl jadomycin B (**S80**) was produced by a marine actinomycete *Micromonospora* sp. strain A5-1 obtained from soft coral *Scleronephthya* sp. in the East China Sea [106]. *Streptomyces* sp. OUCMDZ-1703^†^ associated with a soft coral collected from the South China Sea led to the discovery of two new chlorinated polyketides strepchloritides A and B (**168** and **169**) (Figure 7), together with 1-(3,5-dihydroxyphenyl)ethanone (**S41**). Polyketides **168** and **169** showed moderate cytotoxicity against MCF-7 tumor cells [60].

Aranciamycin K (**S81**) and two known anthracycline derivatives (**170**, **S82**) were produced by coral-associated *Streptomyces* sp. SCSIO 41399. Compound **170** (Figure 7) exhibited moderate in vitro cytotoxicity against the K562 cell lines [62].

A novel gram-positive antibiotic anthracimycin B (**171**) and anthracimycin (**172**) (Figure 7) were isolated from *Streptomyces cyaneofuscatus* M-169 associated with a gorgonian coral collected in the Avilés Canyon. Anthramycin has significant activity against four Gram-positive bacteria (MSSA, MRSA, vancomycin sensitive *Enterococcus faecium*, and vancomycin sensitive *Enterococcus faecalis*) with MIC values less than 0.03 µg/mL. And anthracimycin B was also active against these four Gram-positive bacteria. In addition, anthracimycin displayed anti-tubercular activity against *Mycobacterium tuberculosis* [107].

1-hydroxy-1-norresistomycin (HNM) (**173**) (Figure 7) was produced by *Streptomyces variabilis* obtained from Scleractinia coral *Acropora formosa*. HNM, and inhibited biofilm formation of *E. coli, V. cholerae*, and *S. aureus* with an efficiency of 96%, 92%, and 93%, respectively. Additionally, it also exhibited potent cytotoxic activity against cell lines viz. HMO2 (gastric adenocarcinoma) and HePG2 (hepatic carcinoma) in vitro [108].

Isolation of three novel glycosylated macrolides iseolides A–C (**174**–**176**) (Figure 7) were reported in 2020 from *Streptomyces* sp. DC4-5 is associated with a stony coral *Dendrophyllia*. Iseolides showed potent antifungal activity against a plant pathogen *Glomerella cingulata*, as well as human pathogens *Candida albicans* and *Trichophyton rubrum* with MICs in the range of 0.19–6.25 μg/mL [109].

##### Polyketides Derived from the Ascidian-Associated Actinomycetes

**Figure 8:**
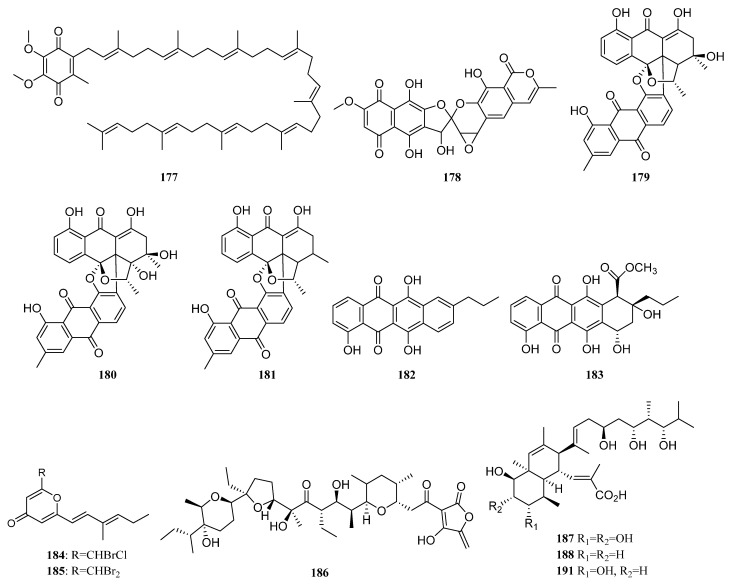

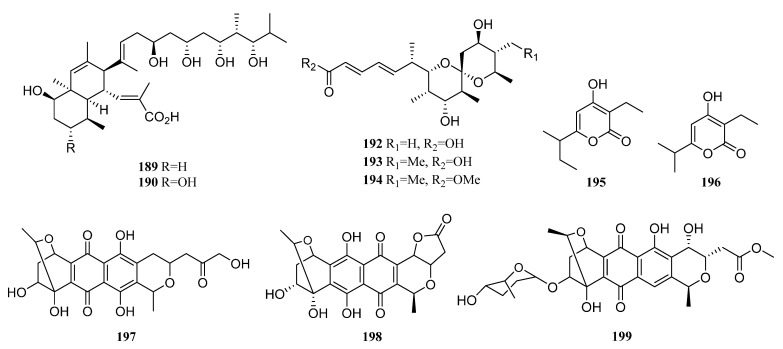
Structures of compounds **177**–**199**.

Ubiquinone Q9 (**177**) (Figure 8) was isolated from *Nocardia* sp. strain KMM 3749, a marine actinomycete associated with an unidentified ascidian. This compound inhibited the development of fertilized eggs of *Strongylocentrotus intermedius* and caused hemolysis of mouse erythrocytes [64,110].

The polyketide griseorhodin A (**178**) (Figure 8) was found to be biosynthesized by *Streptomyces* sp. JP95, which is associated with marine ascidian *Aplidium lenticulum* collected at Heron Island, Queensland, Australia. Griseorhodin A, a member of the rubromycin family, is an inhibitor of human telomerase [64,111].

The isolation of *Streptomyces* sp. #N1-78-1 from sea squirt *Ecteinascidia turbinata* in Puerto Rico led to the purification of bisanthraquinones 1 and 2 (**179**, **180**), as well as derivative 3 (**181**) (Figure 8), the dehydration product of bisanthraquinone 1. Bisanthraquinones 1 and 2 showed potent antimicrobial activities against MRSA (methicillin-resistant *Staphylococcus aureus*) and VRE (vancomycin-resistant *Enterococcus faecalis*), and these three compounds displayed cytotoxic activity against HCT-116 cells [64,112].

Four novel anthracyclinones (**182**, **183**, **S83**, and **S84**) were produced by a strain of *Micromonospora* sp. derived from a Brazilian endemic ascidian *Eudistoma vannamei*. Compounds **182** and **183** (Figure 8) displayed moderate cytotoxic effects on the human colon adenocarcinoma cell line HCT-8 with IC_50_ values of 12.7 and 6.2 μM, respectively [113].

In 2013, *Actinomadura* sp. WMMB499 associated with ascidian *Ecteinascidia turbinata* collected in the Florida Keys led to the discovery of four new halogenated electrophilic polyketides halomadurones A–D (**S85**, **S86**, **184**, **185**). Halomadurones C (**184**) and D (**185**) (Figure 8) showed potent nuclear factor E2-related factor antioxidant response element (Nrf2-ARE) activation [64,114]. In addition, a polyether antibiotic ecteinamycin (**186**) (Figure 8) was also isolated from this strain, acting as an ionophore antibiotic and shows potent antibacterial activity, especially against a wide array of *Clostridium difficile* strains [115].

Five polyol polyketides containing a decalin ring, including four novel nahuoic acids B−E (**187**–**190**) and nahuoic acid A (**191**) (Figure 8) were separated from *Streptomyces* sp. SCSGAA 0027 and associated with gorgonian coral *Melitodes squamata* collected at the South China Sea. Nahuoic acid A (**191**) with an unprecedented carbon skeleton was the first natural product inhibiting the SETD8 lysine methyltransferase and the first selective SETD8 inhibitor. Compounds **187**–**191** showed weak antibiofilm activity against *Shewanella onedensis* MR-1 biofilm [116]. In 2017, strain SCSGAA 0027 was further investigated which led to the isolation of five new spirocyclic polyketides pteridic acids C–G (**S87**, **S88**, **192**–**194**). Compounds E-G (**192**–**194**) (Figure 8) had weak antibacterial activity against *B. subtilis* [117].

The *Streptomyces coelicoflavus* strain HQA809, which is isolated from sea squirt *Styela clava*, produced germicidin (**195**) and 6-isopropyl group-3-ethyl-4-hydroxy-2-pyrone (**196**) (Figure 8). Both two compounds were lethal to *Artemia salina* [64].

The *Streptomyces* sp. PTY087I2 associated with *styela canopus* collected from Bastimentos Park, Bocas del Toro, Panama produced three naphthoquinone derivatives granaticin (**197**), granatomycin D (**198**), and dihydrogranaticin B (**199**) (Figure 8). Co-culture of *Streptomyces* sp. PTY087I2 with human pathogens such as *Bacillus subtilis*, MSSA, and MRSA, respectively, resulted in increased production of these three antibiotics. In addition, co-culture resulted in greatly enhanced biological activity against the above three Gram-positive human pathogens [64,118].

##### Polyketides Derived from the Sea Cucumber-Associated Actinomycetes

**Figure 9:**
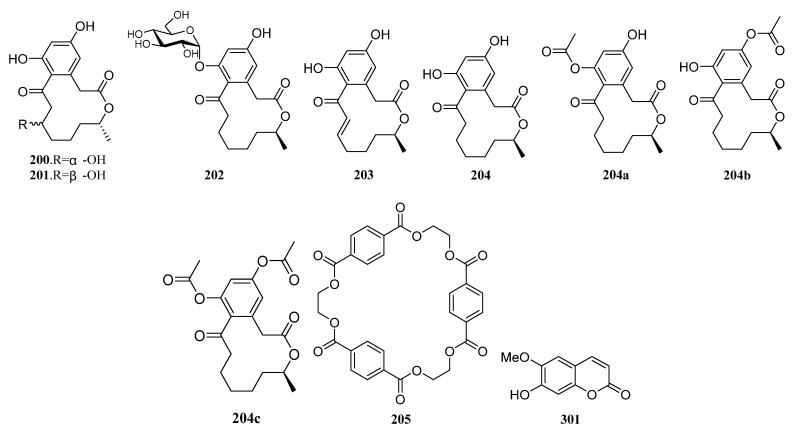
Structures of compounds **200**–**205**, **301**.

Five curvularin macrolides (**200**–**204**) (Figure 9) were isolated from *Pseudonocardia* sp. HS7, cultivated from obtained from the cloacal aperture of the sea cucumber *Holothuria moebii.* Compound **202** is a new macrolide with a rare a-D-glucopyranose substituent. Compounds **200**–**203**, **204a**, and **204c** (the acyl products of **204**) suppressed the proliferation of cancer cell lines, and metabolites **203** is the most active compound with IC_50_ values ranging from 0.59 to 3.39 mM. The 11-hydroxycurvularins **200** and **201** also showed antibacterial activity inhibiting the growth of *Escherichia coli* [119]. Compound **205** (Figure 9), which was isolated from cucumber-associated *Streptomyces* sp. G278 selectively inhibited *Enterococcus faecalis* (MIC: 256 μg/mL). And the known metabolite **301** (Figure 9) with antibacterial and antifungal properties was also discovered from strain G278 [82].

##### Polyketides Derived from the Actinomycetes Associated with Other Marine Invertebrates

**Figure 10:**
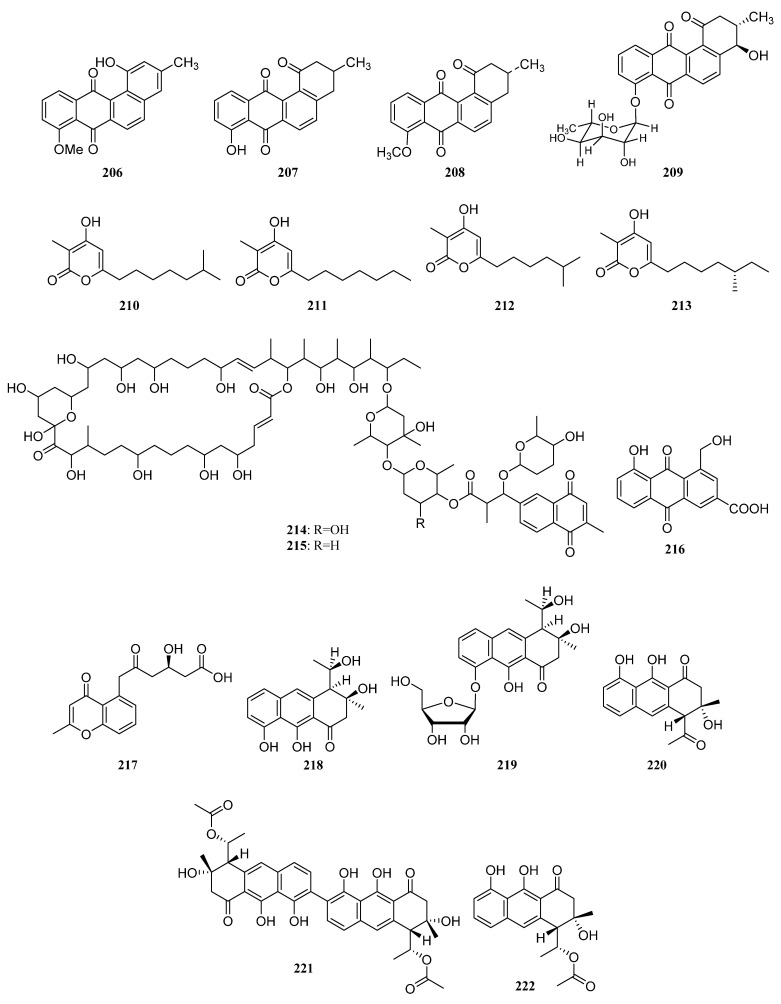
Structures of compounds **206**–**222**.

Three known analogs X- 14881 E (**206**), ochromycinone (**207**), and X-14881 C (**208**), together with a new angucyclines saccharothrixmicine **A** (**209**) (Figure 10), were produced by *Saccharothrix espanaensis* An 113 obtained from a marine mollusk specimen (*Anadara broughtoni*), which was collected from Peter the Great Bay, Sea of Japan, Russia. Compounds **206**–**208** showed significant activity against *C. albicans, B. subtilis, E. faecium, S. aureus*, and *Xanthomonas* sp. pv. *badrii*. And Compound **209** exhibited activity towards *Candida albicans* and *Xanthomonas* sp. pv. *Badrii* [120,121]. Macrolactins E (**S89**) and F (**S90**), together with gilvocarcins M (**S91**) and V (**S92**), have been isolated from an unidentified tunicate-associated *Saccharopolyspora* sp. SS081219 JE-28 [64,77].

Isolation of two novel 3,4,6-trisubstituted α-pyrone derivatives violapyrones H (**210**) and I (**211**) were reported together with known violapyrones B (**212**) and C (**213**) (Figure 10), from *Streptomyces* sp. 112CH148 associated with starfish *Acanthaster planci* collected at Chuuk, Federated States of Micronesia. Violapyrones (**210**–**213**) showed growth inhibitory activity against cancer cell lines at concentrations less than 26.12 μg/mL. Wherein compound **210** showed the highest cytotoxic activity against the HCT-15 cell line with a GI_50_ value of 1.10 μg/mL. Additionally, violapyrones B and C were demonstrated to have antibacterial activities. Therefore, it may be noteworthy that each compound has structural similarities, but showed different activities. The results suggested that the length of the aliphatic side chain and the position of the methyl group affected the activity. Furthermore, violapyrones having an isomethyl group in the alkyl side chain showed better activity than others [122].

In 2015, Marta Pérez et al. reported the discovery of two novel polyhydroxyl macrolides PM100117 (**214**) and PM100118 (**215**) (Figure 10) from *Streptomyces caniferus* GUA-06-05-006A associated with marine polychaete *Filograna* sp. collected at Guadalupe Island in the Pacific Ocean. Both macrolides **214** and **215** displayed strong cytotoxicity against tumor cells and weak antifungal activity against *Candida albicans* ATCC10231 [123].

Two new compounds (**216**, **217**) (Figure 10) and five known ones (**S93**–**S97**) were produced by the sea anemone-derived *Streptomyces* sp. ZZ406. New compounds **216** and **217** showed potent activity in inhibiting the proliferation of different glioma cells and downregulating the expressions of glioma metabolic regulators. Compound **216** was active against the proliferation of different glioma cells with IC_50_ values of 4.7 to 8.1 μM, high selectivity index (>12.3 to 21.3), and good stability in human liver microsomes [83]. A novel flavonoid derivative flavoside A (**S98**) and the known angucyclinone PD116740 (**126**) were isolated from the sea urchin-derived *Streptomyces* sp. HDa1 [86].

Two julichrome monomers julichromes Q_11_ (**218**) and Q_12_ (**219**) (Figure 10), along with five known julichromes Q_10_, Q_3·5_, Q_3·3_, Q_6·6_, Q_6_ (**220**, **S99**, **S100**, **221**, **222**) were separated from the marine gastropod mollusk *Batillaria zonalis*-associated *Streptomyces sampsonii* SCSIO 054. In addition, four known anthraquinones chrysophanol (**S101**), 4-acetylchrysophanol (**S102**), islandicin (**S103**), and huanglongmycin A (**S104**) were also discovered. Julichrome Q_12_ (**219**) was found to display antibacterial activity against *Micrococcus luteus* and *Bacillus subtilis* with MICs of 2.0 and 8.0 μg/mL. What’s more, compounds **218**, **220**–**222** (Figure 10) also showed inhibitory activities against methicillin-resistant *Staphylococcus aureus*, *S. aureus,* and *S. simulans* AKA1 with MIC values ranging from 8 to 64 μg/mL [124].

##### Polyketides Derived from the Actinomycetes Associated with Marine Vertebrates

**Figure 11:**
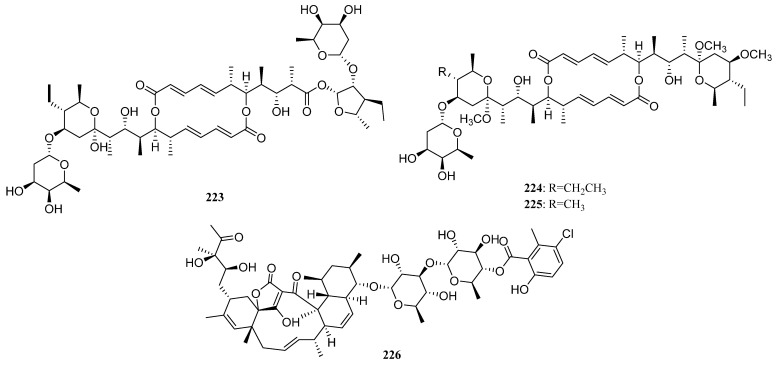
Structures of compounds **223**–**226**.

Further investigation for metabolites of this strain has led to the isolation of three additional novel cytotoxic metabolites designated as halichoblelide A–C (**223**–**225**) (Figure 11). Halichoblelide A was active against the murine P-388 cell line (ED_50_ 0.63 μg/mL), and all three novel metabolites exhibited significant cytotoxicity against the 39 human cancer cell lines [125,126].

Ochoa et al. reported the isolation of a new glycosylated polyketide phocoenamicin (**226**) (Figure 11) in 2017, from a *Micromonospora* strain obtained from five marine mammals. It showed potent activity against a broad panel of Gram-positive animals and plants such as the intestinal pathogen *Clostridium difficile* (MIC: 2.6 μM) [127].

#### 3.1.3. Peptides

Most peptides from actinomycetes are circular and contain further rare structural elements, such as chromophore or unusual amino acids.

##### Peptides Derived from the Sponge-Associated Actinomycetes

**Figure 12:**
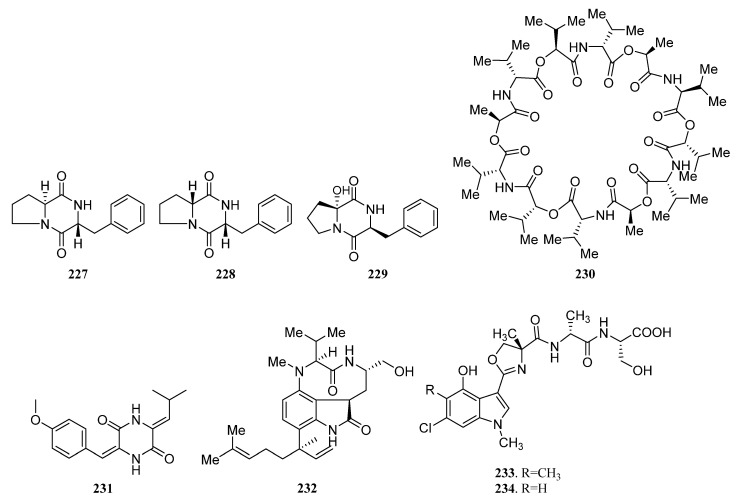

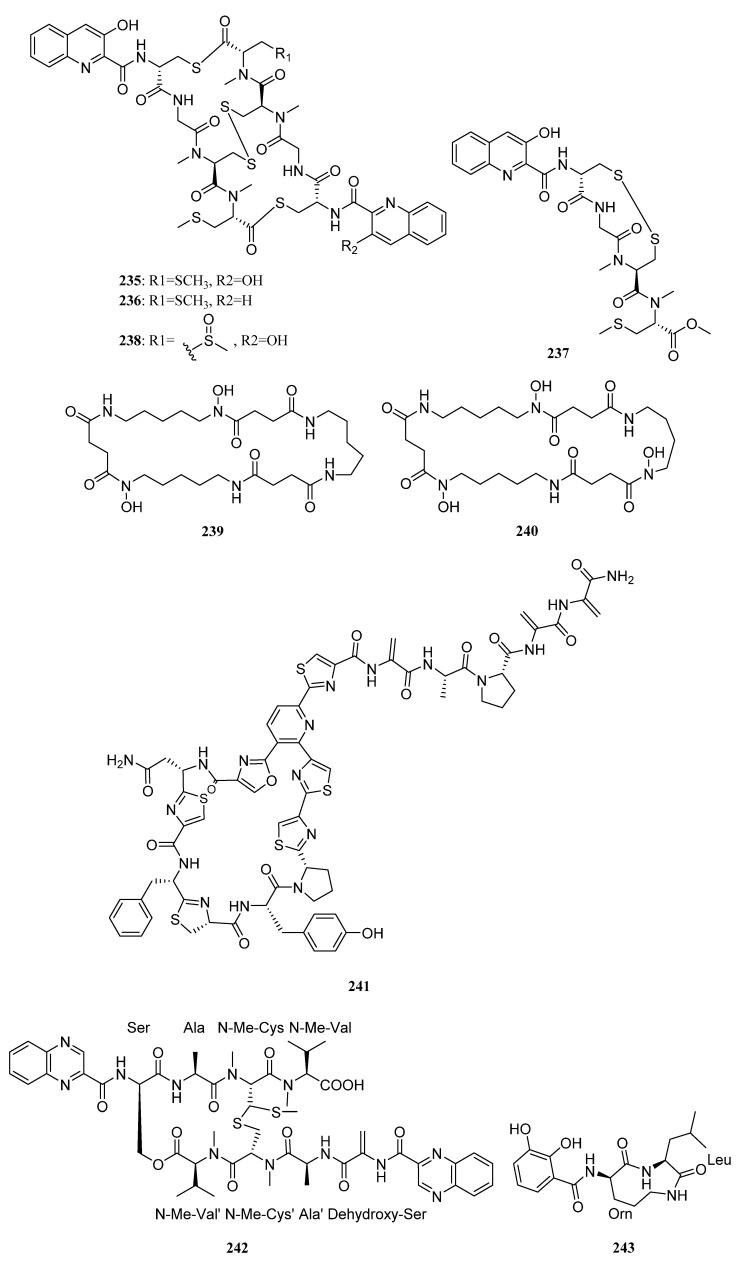

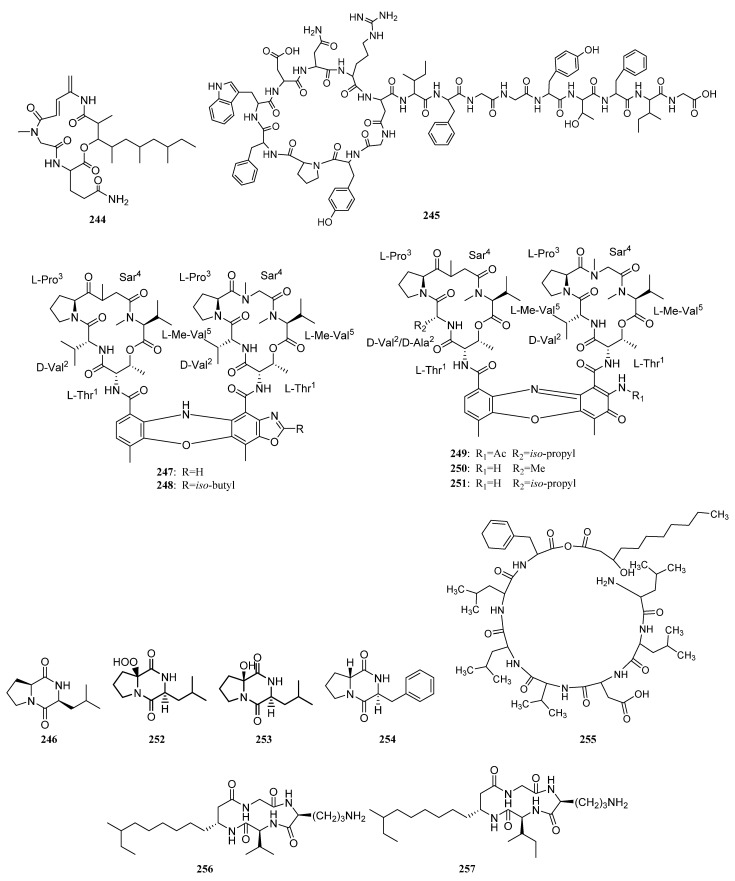

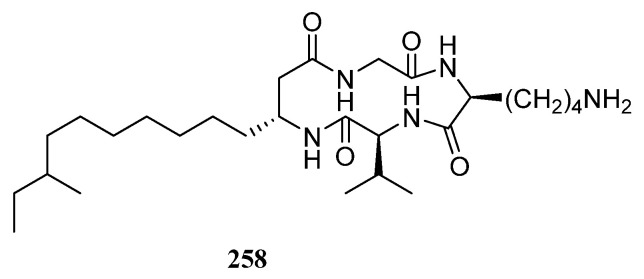
Structures of compounds **227**–**258**.

*Streptomyces* sp. DA18 was separated from marine sponge *Craniella australiensis* collected at Sanya Island in the South China Sea, from which diketopiperazines (DKPs) (**227**, **228**, **229**, **S105**) (Figure 12) were discovered. Compounds **227** and **228** showed antimicrobial activity, of which **228** also had antifouling activity [128].

The cyclic depsipeptide valinomycin (**230**) (Figure 12) was isolated from *Streptomyces* sp. strains 22 and 34 associated with the sponges *Aplysina aerophoba* and *Axinella polypoides*, respectively. It exhibited significant inhibitory activities against the parasites *Leishmania major* (IC_50_ < 0.11 μM) and *Trypanosoma brucei brucei* (IC_50_ = 0.0032 μM). Additionally, it was active against 293T kidney epithelial cells (IC_50_ 11.24 µM) and J774.1 macrophages (IC_50_ < 0.10 µM) [9,21].

A diketopiperazine (**231**) (Figure 12) with weak cytotoxic activity, known as a synthetic compound before, was first reported to be discovered from *Nocardiopsis* strain HB383 [92]. The new teleocidin analog JBIR-31 (**232**) (Figure 12) was purified from the obligate marine *Streptomyces* sp. NBRC 105896 separated from *Haliclona* sp. collected offshore Tateyama City (Chiba Prefecture, Japan). The compound displayed weak cytotoxic activity against human cervical carcinoma HeLa cells with an IC_50_ value of 49 μM [9,129].

In 2010, Motohashi et al. reported the discovery of two new modified indole-containing peptides JBIR-34 (**233**) and JBIR-35 (**234**) (Figure 12) from *Streptomyces* sp. Sp080513GE-23 associated with *Haliclona* sp. Compounds **233** and **234** exhibited DPPH radical scavenging activity with IC_50_ values of 1.0 and 2.5 mM [130].

Isolation of two new peptides JBIR-56 (**S106**) and JBIR-57 (**S107**) were reported in 2011 from the new isolate *Streptomyces* sp. SpD081030SC-03, which was obtained from an unidentified sponge collected in Ishigaki, Okinawa, Japan [9,131].

The potent cytotoxic thiodepsipeptide thiocoraline (**235**) (Figure 12), which was first isolated in 1997 from the mycelia of *Micromonospora marina*, and five new analogs of thiocoraline (**236**, **S108**, **S109**, **237**, **238**) were discovered in 2011 from *Verrucosispora* sp. strain WMMA107, which was separated from *Chondrilla caribensis* f. *caribensis* (Florida Keys, USA). 22′-Deoxythiocoraline (**236**), thiochondrilline C (**237**), and 12′ -sulfoxythiocoraline (**238**) (Figure 12) showed significant cytotoxicity against the A549 human cancer cell line with EC_50_ values of 0.13, 2.86 and 1.26 μM, respectively [9,132].

A sponge-derived *Streptomyces* sp. strain RV15 was reported to produce four new cyclic lipopeptides cyclodysidins A–D (**S110**–**S113**) in 2012 [9,133]. Two novel cyclic peptides (**239** and **240**) (Figure 12), along with the previously reported nocardamine (**S114**), were isolated from *Streptomyces* M1087 associated with an unidentified sponge. The new compounds **239**–**240** exhibited weak inhibition against the recombinant enzyme sortase B with EC_50_ values of 88.3 and 126.4 µg/mL [134].

*Streptomyces* sp. NIO 10068 derived from a sponge collected from the western coast of India produced linear dipeptides proline–glycine (**S115**) and N-amido-α- proline (**S116**) [135]. Kocurin (**241**) (Figure 12), a new member of the thiazolyl peptide family of antibiotics, was produced by *Kocuria palustris.* F-276,345 is associated with a marine sponge that was collected in Florida Keys. Kocurin showed potent activity against Gram-positive bacteria (MRSA) with MIC values of 0.25–0.5 μg/mL [136,137].

Xin Zhen et al. reported isolation and identification of two new metabolites quinomycin G (**242**) (Figure 12) and cyclo-(L-Pro-4-OH-L-Leu) (**S117**) in 2015, from sponge-associated *Streptomyces* sp. LS298. Quinomycin G (**242**) with a terminal double bond in one of the Ser groups exhibited moderate antibacterial activities against *Staphylococcuse pidermidis*, *S. aureus*, *Enterococcus faecium,* and *E. faecalis* with MIC values ranging from 16 to 64 μg/mL. In addition, it showed potent anti-tumor activities and the highest activity was observed against the Jurkat cell line (human T-cell leukemia) with an IC_50_ value of 0.414 μM [36].

A new cyclic dipeptide petrocidin A (**243**) (Figure 12) was discovered from sponge-derived *Streptomyces* sp. SBT348 exhibited significant cytotoxicity towards the human promyelocytic HL-60 and the human colon adenocarcinoma HT-29 cell lines [45]. New cyclic depsipeptide rakicidin F (**244**) (Figure 12) and known rakicidin C (**S118**) were isolated from *Streptomyces* sp. GKU 220 is associated with a marine sponge sample collected in Andaman sea, Ranong, Thailand. Rakicidin F (**244**) showed growth inhibitory activity against *B. subtilis* and *E. coli* at the dosage of 25 μg per disk [138].

Isolation of a novel antibacterial peptide actinokineosin (**245**) (Figure 12) was reported in 2016, from sponge-associated *Actinokineospora spheciospongiae* DSM45935^T^. Actinokineosin was active against *Micrococcus luteus* with an inhibition zone diameter of 8.0 mm at 50 μg/disk [139]. Four diketopiperazine compounds **S119**–**S122** were purified from the sponge-derived *Streptomyces* sp. G246 [56]. And three known compounds **246** (Figure 12) and **S120**, **S123** were separated from marine sponge-associated *Streptomyces* sp. G248 [55].

Four new D-type actinomycin analogs actinomycins D1-D4 (**247**–**250**) and actinomycin D (**251**) (Figure 12) were discovered from *Streptomyces* sp. LHW52447 associated with *Phyllospongia foliascens* obtained from the Xisha Islands in the South China Sea. Actinomycins D1 (**247**) and D2 (**248**) introduced an oxazole unit into the central phenoxazinone chromophore and exhibited more potent activities against three strains of MRSA with MIC values of 0.125–0.25 μg/mL than that of actinomycins D3–D4 (MIC = 0.5–1.0 μg/mL), which indicated that the incorporation of the oxazole unit would enhance the antibacterial activity. In addition, the cytotoxicity evaluation against human lung WI38 embryonal fibroblasts suggested that the incorporation of oxazole unit could decrease the cytotoxicity of actinomycins on human normal cells [140]. The SAR studies had indicated that amino acid substitutions in different positions of the peptides influenced biological potencies. And the oxidation level of the β-ring proline residue influenced both cytotoxic and antibacterial activity. The single most important structure-activity factor in the peptide moieties is, however, the integrity of the cyclic structure. In addition, the configuration of 4-hydroxyl group points has a strong influence on activity [141,142].

*Streptomyces* sp. Call-36 isolated from sponge *Callyspongia* sp. collected in the Red Sea was reported to produce a new diketopiperazine actinozine A (**252**), cyclo(2-OH-_D_-Pro-_L_-Leu) (**253**), cyclo(_D_-Pro-_L_-Phe) (**254**) (Figure 12), and cyclo(_L_-Pro-_L_-Phe) (**S123**). Compounds **252** and **253** displayed potent activity against *S. aureus* and were moderately active against *C. albicans*. Compound **254** exhibited moderate and selective activity against HCT-116 with an IC_50_ of 32.7 µM, while cyclo (_L_-Pro-_L_-Phe) (**S123**) was inactive, indicating that the D/L configuration of Pro had an important effect on the activity [143].

A novel anti-infective molecule nesfactin (**255**) (Figure 12) was isolated from sponge-associated *Nesterenkonia* sp. MSA31 and active against multidrug-resistant *Pseudomonas aeruginosa* by inhibiting the phenotypic expression of virulence factors [144]. Three antifungal cycle peptides Rhodopeptins C1, C2, and B5 (**256**–**258**) (Figure 12) were obtained from *Rhodococcus* sp. UR59 is associated with marine sponge *Callyspongia* sp. [59].

##### Peptides Derived from the Coral-Associated Actinomycetes

Thiocoraline (**235**) is a thiodepsipeptide antitumor antibiotic isolated from *Micromonospora* sp. L-13-ACM2-092 is associated with a soft coral collected in the Indian Ocean off the coast of Mozambique. Thiocoraline had an inhibitory effect on DNA polymerase α. In addition, it displayed potent cytotoxicity and strong activity against Gram-positive bacteria [145,146].

#### 3.1.4. Peptides Derived from the Ascidian-Associated Actinomycetes

**Figure 13:**
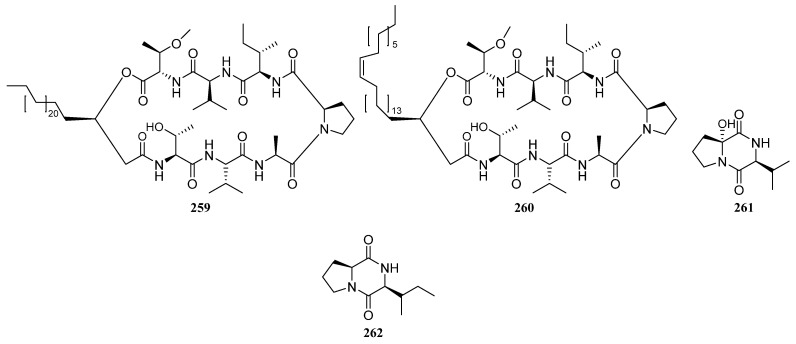
Structures of compounds **259**–**262**.

In 2012, Wyche et al. reported the discovery of five new lipopeptide peptidolipins B–F (**259**, **260**, **S124**–**S126**) (Figure 13) from a strain of *Nocardia* sp., which was cultivated from *Trididemnum orbiculatum*. Peptidolipins B and E were active against MRSA and MSSA [64,147]. Four known diketopiperazines cyclo (6-OH-_D_-Pro-_L_-Phe) (**229**), bacillusamide B (**261**) (Figure 13), cyclo (_L_-Pro-_L_-Leu) (**246**), and cyclo (_L_-Pro-_L_-Ile) (**262**) (Figure 13), were separated from a colonial ascidian-associated *Streptomyces* sp. Did-27 and displayed cytotoxicity against cancer cell lines HCT-116, HepG2, and MCF-7 [64,72].

##### Peptides Derived from the Fish-Associated Actinomycetes

**Figure 14:**
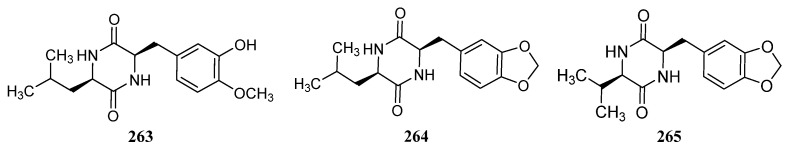
Structures of compounds **263**–**265**.

Isolation of three new 2,5-diketopiperazines (2, 5-DKPs) (**263**–**265**) (Figure 14) was reported in 2016, from *Streptomyces* sp. MNU FJ-36 obtained from the intestinal fabric of *Katsuwonus* sp. All novel compounds displayed weak cytotoxicity against the A-549 cell line, and compounds **264** and **265** also exhibited weak inhibitory activity against HCT-116 cell line [148].

##### Peptides Derived from the Actinomycetes Associated with Other Marine Animals

**Figure 15:**
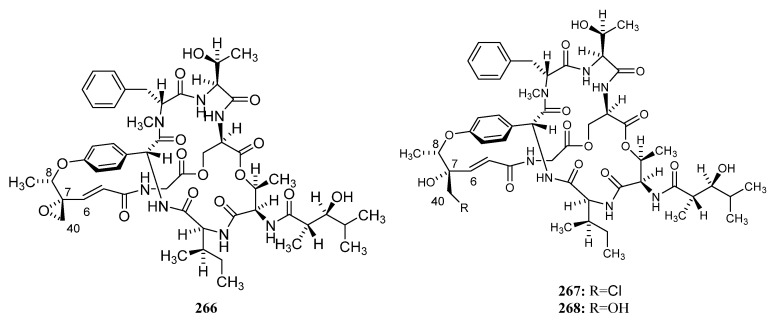

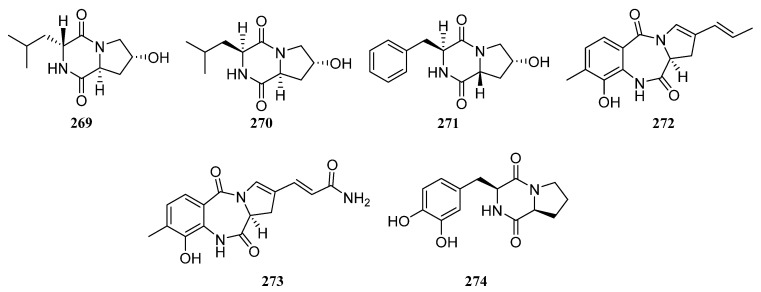
Structures of compounds **266**–**274**.

Five cyclic depsipeptides with unusual structures salinamides A, B (**266**, **267**) (Figure 15) and salinamides C–E (**S127**–**S129**) were discovered from *Streptomyces* sp. CNB-091 obtained from the surface of jellyfish *Cassiopeia xamachana* collected in the Florida Keys. Salinamides A and B exhibited moderate antibiotic activity against Gram-positive bacteria. Additionally, the results of phorbol ester-induced mouse ear edema assay showed that salinamides A and B displayed significant topical anti-inflammatory activity using [149,150]. In 2014, salinamide F (**268**) (Figure 15), a new bicyclic depsipeptide, was also reported to be separated from this strain, possessing significant RNAP-inhibitory and antibacterial activity like salinamide A [151].

Three known diketopiperazines **269**–**271** (Figure 15) produced by *Saccharothrix espanaensis* An 113 were active against *Vibrio alginolyticus* and *Vibrio parahaemolyticus* [121]. Thiocoraline (**235**) was separated from *Micromonospora* sp. ML1 isolated from a mollusk collected from the Indian Ocean coast of Mozambique [152]. Limazepines G (**272**) and H (**273**) (Figure 15) were discovered from *Streptomyces seoulensis* IFB-A01 cultivated from the gut of shrimp *Penasus orientalis.* Compounds **272** and **273** showed potent neuraminidase (NA) inhibitory activity with IC_50_ values of 7.50 and 7.37 μmol/L, respectively [153].

Mengxuan Chen et al. reported isolation and identification of a new compound **S130** and the known valinomycin (**230**) in 2018, from sea anemone-associated *Streptomyces* sp. ZZ406. Valinomycin (**230**) was active against the proliferation of different glioma cells and downregulating the expressions of glioma metabolic regulators [83]. In addition, the valinomycin was also isolated from sponge-derived *Streptomyces* sp. strains 22 and 34 that have been mentioned above. Compound **274** (Figure 15) was discovered from the sea urchin-associated *Streptomyces* sp. HDa1 displayed weak activity against the Gram-negative bacteria *Vibrio harveyi* with an inhibition zone of 1.5 mm [85].

#### 3.1.5. Terpenoids

**Figure 16:**
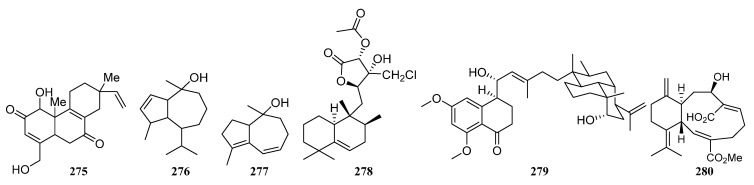
Structures of compounds **275**–**280**.

The new diterpene JBIR-65 (**275**) (Figure 16) was separated from the *Actinomadura* sp. SpB081030SC-15 associated with an unidentified sponge collected offshore Ishigaki Island, Japan. JBIR-65 showed weak protection of neuronal hybridoma N18-RE-105 cells from L-glutamate toxicity with an EC_50_ value of 31 μM [9,154].

Five sesquiterpenes (**276**, **277**, **S131**–**S133**) were isolated from *Streptomyces* sp. (ZJG1) cultivated from stony corals collected in the South China Sea. Compound **277** (Figure 16) showed great scavenging activity, hemolytic activity, and acetylcholinesterase inhibitory activity. Compound **276** (Figure 16) exhibited well free radical scavenging and acetylcholinesterase inhibitory activity [155].

Isolation of two novel halimane-type diterpenoids micromonohalimanes A (**S134**) and B (**278**) (Figure 16) was reported in 2016 from *Micromonospora* sp. WMMC-218 separated from the ascidian *Symplegma brakenhielmi*. Micromonohalimane B showed moderate activity against methicillin-resistant *Staphylococcus aureus* [156].

Guanahanolide A, a meroterpenoid featured with an unprecedented sesterterpene skeleton was obtained from *Streptomyces* sp. RKBH-B7 is associated with octocoral *Eunicea*. Guanahanolide A (**279**) (Figure 16) showed moderate cytotoxicity against human cancer cell lines MCF-7, HTB-26, and HCT-116 [157].

Microeunicellols A (**280**) (Figure 16) and B (**S135**), two novel eunicellin diterpenoids, were reported in 2020 from the culture of *Streptomyces albogriseolus* SY67903 associated with the gorgonian *Muricella sibogae* collected at the South China Sea. Microeunicellol A exhibited cytotoxicities against several human cancer cell lines [158].

#### 3.1.6. Steroids

**Figure 17:**
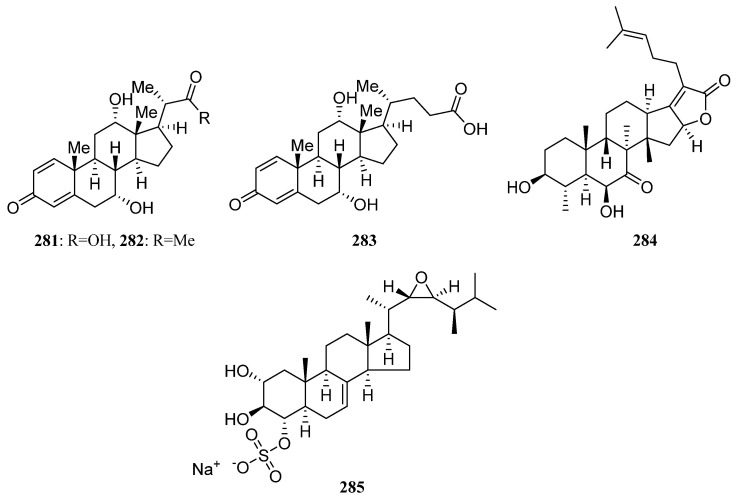
Structures of compounds **281**–**285**.

3-keto sterols bendigoles D–F (**281**–**283**) (Figure 17) were discovered from *Actinomadura* sp. SBMs009 is derived from the sponge *Suberites japonicus*. They showed anti-inflammatory activity based on NF-kB inhibition and glucocorticoid receptor–protein binding properties. Among them, bendigole F exhibited the highest activity against translocation of GFP-labeled NF-kB into the nucleus of hamster ovary CHO cells in vivo with an IC_50_ of 71 μM. The three sterols displayed activity against the glucocorticoid receptor translocation and bendigole D was the most potent. Bendigole D showed mild cytotoxicity against the L929 murine aneuploid fibrosarcoma with an IC_50_ of 30 μM [9,159].

Streptoseolactone (**284**) (Figure 17), a novel metabolite isolated from *Streptomyces seoulensis* IFB-A01, showed a significant inhibitory effect on NA in a dose-dependent manner, and the IC_50_ value was 3.92 μmol/L [153].

Manadoperoxide H (**285**) (Figure 17) and acanthosterol sulfate F (**S136**) were isolated from sponge-associated *Streptomyces* sp. RM66. Manadoperoxide H exhibited antitrypanosomal activity against *Trypanosoma brucei rhodesiense* with an IC_50_ value of 0.375 μg/mL [58].

#### 3.1.7. Other Structure Classes

**Figure 18:**
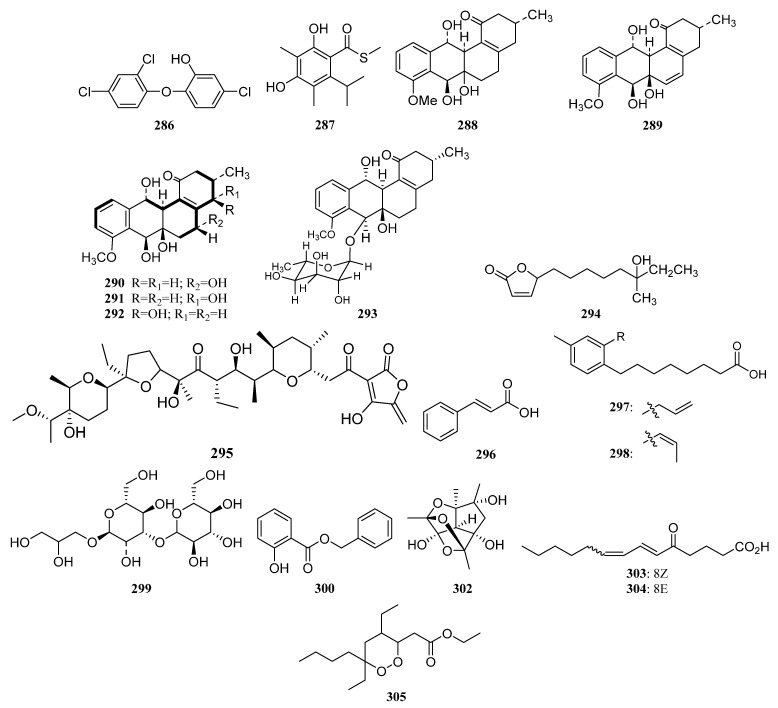
Structures of compounds **286**–**300**, **302**–**305**.

Lutoside (**S137**) was purified from *Micrococcus luteus* R-1588-10 associated with sponge *Xestospongia* sp. collected at Noumea, New Caledonia. Additionally, the previously known synthetic 2,4,4′-trichloro-2′-hydroxydiphenylether (**286**) (Figure 18) was also discovered from the strain R-1588-10, and it was active against *Staphylococcus aureus*, *Vibrio anguillarum*, and *Candida albicans* [9,160].

Isolation of a new cytotoxic metabolite (**287**) (Figure 18) characterized as S-methyl-2,4-dihydroxy-6-isopropyl-3,5-dimethylbenzothioate was reported in 2008 from *Streptomyces* sp. (LA3L2). Two known compounds montagnetol (**S138**) and erythrin (**S139**) were also isolated from *Streptomyces* sp. (LA3L2) that is the first reported actinomycete to produce these lichen-related compounds. In addition, chromomycin A2 (**S140**), chromomycin A3 (**S141**), and chromomycin 02-3D (**S142**) were reported to be separated from *Streptomyces* sp. (LA3L1) [76].

Three new angucyclinones saccharothrixins A (**290**), B (**291**), and C (**292**), together with two known analogs X-14881 A (**288**) and X-14881 B (**289**) (Figure 18), were produced by sponge-associated *Saccharothrix espanaensis* An 113. In addition, a new angucyclines saccharothrixmicine B (**293**) (Figure 18) was also reported from this strain in 2010, which displayed activity against *Candida albicans* and *Xanthomonas* sp. pv. *badrii.* Compound **288** exhibited moderate inhibitory activity against *C. albicans* and weak activity against *B. subtilis* and *E. faecium.* Compound **289** possessed significant activity against *C. albicans, B. subtilis, E. faecium, S. aureus*, and *Xanthomonas* sp. pv. *badrii*, whereas activities of compounds **290**–**292** against *B. subtilis, E. faecium,* and *Xanthomonas* sp. pv. *badrii* were only modest [120,121].

*Streptomyces* sp. strain T03 derived from the sponge *Tethya* sp. led to the identification of butenolide (**294**) (Figure 18), which exhibited anti-*trypanosoma* activity against *Trypanosoma brucei brucei* (IC_50_ = 31.77 μM) [9,21]. Ecteinamycin (**295**) (Figure 18) was obtained from *Actinomadura* sp associated with a sea squirt *Ecteinascidia turbinate.* It showed potent activity against *Clostridium difficile* NAP1/B1/027 [64,115].

New nucleoside derivative (**S143**) and two known compounds **S144** and **S145** were isolated from *Streptomyces microflavus* strain No. HVG29 is associated with the marine sponge *Hymeniacidon perlevis* collected from the coast of Dalian (China). Compounds **S143**–**S145** were the first time to isolate deoxyuridine structures from *S. microflavus* associated with sponges [161].

*Streptomyces* sp. NIO 10068 derived from a marine sponge produced cinnamic acid (**296**) (Figure 18), which was active against QS (quorum sensing)-mediated virulence factors in *Pseudomonas aeruginosa*. Cinnamic acid was proven to possess previously undescribed QS antagonist properties. In addition, it is demonstrated to display bactericidal activity in the present study [135].

In 2014, Ellis et al. reported isolation and identification of two novel trialkyl-substituted aromatic acids, solwaric acids A and B (**297**, **298**) (Figure 18), together with the known 2,4,6-triphenyl-1-hexene (**S146**) from *Solwaraspora* sp. WMMB329 is associated with ascidian *Trididemnum orbiculatum*. The two novel compounds demonstrated antibacterial activity against MRSA and MSSA [64,162].

Four unusual glycoglycerolipids (**299**, **S147**–**S149)** and diphosphatidylglycerol (**S150**) were produced by *Microbacterium* sp. HP2 associated with sponge *Halichondria panacea* collected at the Adriatic coast, Rovinj, Croatia. The major compound **299** (Figure 18) displayed antitumor activity by inhibiting the growth of tumor cell lines HM02 and Hep G2 with GI_50_ values of 0.38 and 2.7 μg/mL, respectively [9,163].

Isolation of a new oxaphenalene derivative (**S151**) was reported in 2017 from *Streptomyces griseorubens* sp. ASMR4 is associated with an unidentified soft coral collected in the Red Sea at the Hurghada coast, Egypt. Additionally, along with metabolite S144, seven other known metabolites **S152**–**S158** were also discovered from the strain ASMR4 [164].

Phenylacetic acid methyl ester (**S159**) and tryptophan (**S160**) were produced by *Rhodococcus* sp. UA13 [43]. Two known compounds 1-hydroxy-2-naphthoic acid (**S161)** and 1,4-dihydroxy-2-naphthoic acid (**S162**) were isolated from the axenic culture of *Saccharomonospora* sp. UR22 [47]. In addition, L-tryptophan (**S160**) and L-phenylalanine (**S163**) were produced by the sponge-associated *Streptomyces* sp. G246 [56]. The known metabolite **300** (Figure 18) isolated from sea cucumber-derived *Streptomyces* sp. G278 exhibited antimicrobial activity [82]. GTRI-02 (**S164**) was produced by the sea anemone-derived *Streptomyces* sp. ZZ406 [83].

In 2019, Shaala et al. reported the discovery of two new nucleosides thymidine-3-mercaptocarbamic acid (**S165**) and thymidine-3-thioamine (**S166**) from sponge-associated *Streptomyces* sp. Call-36 [143]. An intriguing cage-like polyhemiketal nesteretal A (**302**) (Figure 18) was produced by *Nesterenkonia halobia* E5.1 obtained from a scleractinian coral *Platygyra.* Nesteretal A is a highly oxygenated compound featuring an unprecedented 5/5/5/5 tetracyclic scaffold. It showed a weak retinoid X receptor-α (RXRα) transcriptional activation effect [165].

A pair of geometrically isomeric unsaturated keto fatty acids (6*E*,8*Z*)/(6*E*, 8*E*)-5-oxo-6,8-tetradecadienoic acids (**303**, **304**) (Figure 18) were identified from *Micrococcus* sp. associated with a stony coral *Catalaphyllia* sp. Compounds **303** and **304** with the unprecedented 2,4-dienone system both showed antibacterial activity against the plant pathogen *Rhizobium radiobacter* (preferred **303**) and the fish pathogen *Tenacibaculum maritimum* (preferred **304**). In addition, compounds **303** and **304** displayed agonistic activity against peroxisome proliferator-activated receptors (PPARs) with an isoform specificity towards PPARα and PPARγ [166].

Isolation of four metabolites ethyl plakortide Z (**305**) (Figure 18), seco-plakortide Z (**S167**), actinopolysporin B (**S168**), and acanthosterol G (**S169**) was reported in 2021 from sponge-associated *Streptomyces* sp. RM66. Peroxidessethyl plakortide Z was active against solid tumor and L-1210 leukemia cell lines in vitro [58].

### 3.2. Natural Products of the Actinomycetes Derived from Marine Plants, Macroalgae, Cyanobacteria, and Lichens

#### 3.2.1. Alkaloids

##### Alkaloids Derived from the Brown Algae Associated Actinomycetes

**Figure 19:**
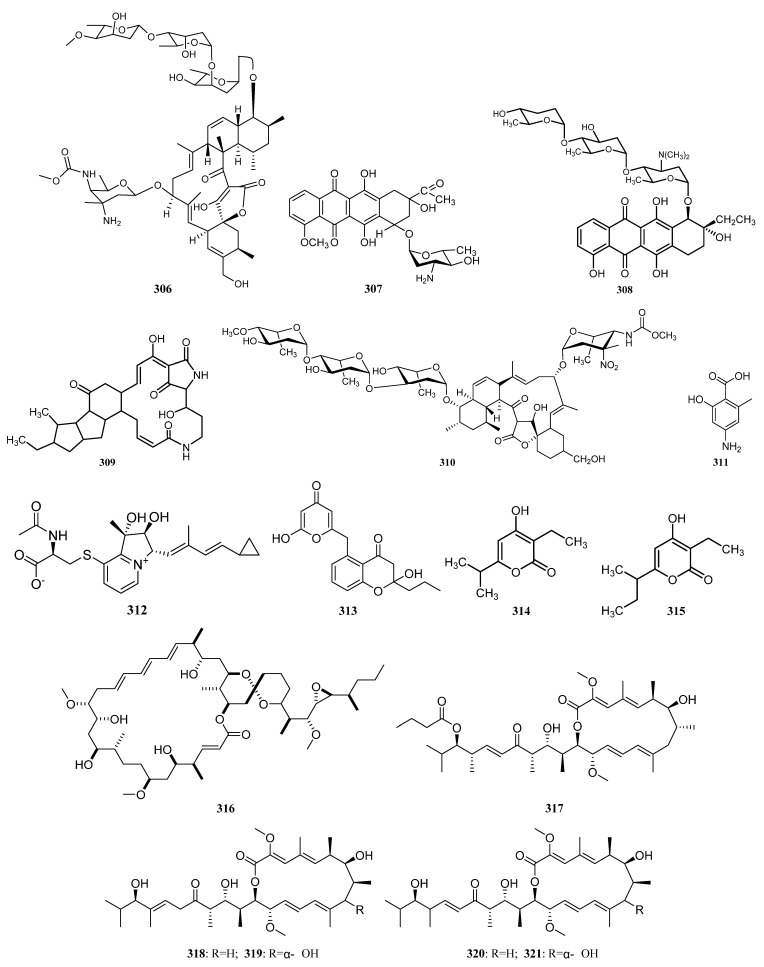

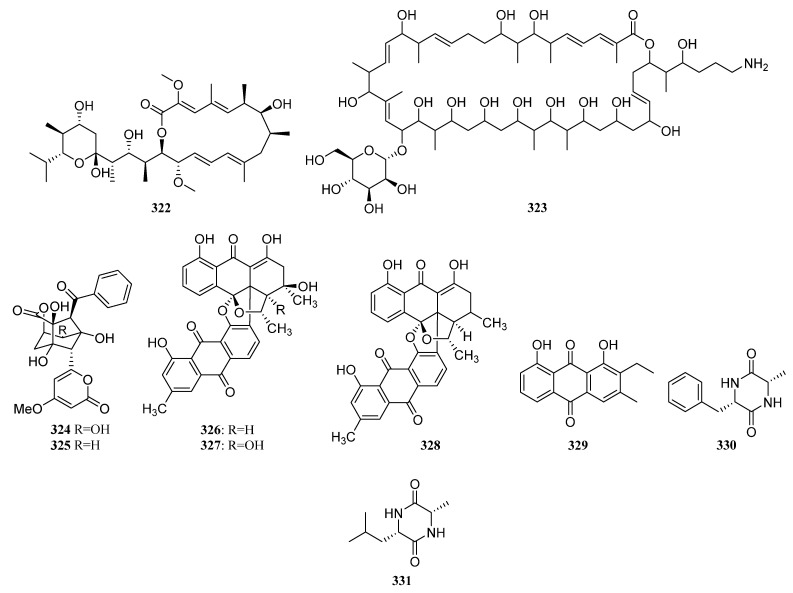
Structures of compounds **306**–**331**.

Two new anti-inflammatory compounds lobophorins A (Figure 19) and B (**306**, **109**) were produced by an unidentified actinomycete strain CNC-837 obtained from the surface of the Caribbean brown alga *Lobophora variegate*. The new compounds, distantly related to antibiotics of the kijanimicin class, are potent inhibitors of topical PMA-induced edema in the mouse ear assay when administered either topically or IP [167].

*Streptomyces cyaneofuscatus* M-27 and *Streptomyces carnosus* M-40 were associated with diverse intertidal marine brown macroalgae (*P*hyllum heterokontophyta**, *Fucus spiralis*, and *Cystoseira baccata*) from the central Cantabrian Sea. *Streptomyces cyaneofuscatus* M-27 produced several antitumor antibiotics of the anthracycline family, of which two antibiotics were identified as daunomycin (**307**) and cosmomycin B (**308**) (Figure 19). And it also led to the isolation of an antifungal macrolactam maltophilin (**309**) (Figure 19). In addition, lobophorine B (**310**) (Figure 19) was separated from *Streptomyces carnosus* M-40 derived from macroalgae *Cystoseira baccata* with anti-inflammatory and antituberculosis properties [168].

Isolation of a new phenolic acid derivative 4-amino-6-methylsalicylic acid (**311**) (Figure 19) was reported in 2019 from *Nocardiopsis* sp. AS23C was obtained from *Sargassum arnaudianum* collected in the Red Sea at Hurghada coast, Egypt. The extract exhibited antibacterial activity against the Gram-positive *Staphylococcus aureus*, *Bacillus subtilis* ATCC6051, and *Streptomyces viridochromogenes* Tü 57 [169].

##### Alkaloids Derived from the Green Algae Associated Actinomycetes

A unique indolizinium alkaloid streptopertusacin A (**312**) (Figure 19) and a novel N-arylpyrazinone derivative streptoarylpyrazinone A (**S170**) were separated from the seaweed-associated *Streptomyces* sp. HZP-2216E obtained from sea lettuce *Ulva pertusa*, a traditional Chinese medicine. They are both existing as zwitterion and streptopertusacin A showed moderate activity against the growth of MRSA [170,171].

Three new compounds (**S171**–**S173**), together with the known benzamides 2-acetamido-3-hydroxybenzamide (**S174**), 2-amino-3-hydroxybenzamide (**S175**), and 2-aminobenzamide (**S176**), were isolated from the *Streptomyces* sp. ZZ502 is associated with the seaweed *Ulva conglobatea* collected in the East China Sea [172].

##### Alkaloids Derived from the Actinomycetes Associated with Lichens

Isolation of a novel metabolite **S177** was reported in 2013 from *Streptomyces cavourensis* YY01-17 separated from the lichens grown in the Antarctic area [173].

#### 3.2.2. Polyketides

##### Polyketides Derived from the Brown Algae Associated Actinomycetes

Polyketide **313** (Figure 19) [2-hydroxy-5-((6-hydroxy-4-oxo-4H-pyran-2-yl) methyl)-2-propylchroman -4-one] was isolated as the major metabolite together with three related known phaechromycins B, C, and E (**S178**–**S180**) from *Streptomyces sundarbansensis* strain WR1L1S8 associated with brown algae *Fucus* sp. collected in Bejaia coastline, Algeria. Compound **313** stood out as the most active of the series, showing a selective antibacterial activity against MRSA with a MIC of 6 μΜ [174].

Germicidins A (**314**) and B (**315**) (Figure 19), discovered from *Streptomyces carnosus* M-40, displayed a physiological role in spore germination and hypha elongation. Galtamycin B (**S181**) produced by *Streptomyces cyaneofuscatus* M-27 associated with *Fucus spiralis* was first isolated from *Streptomyces* or marine environments [168].

In 2018, Kim et al. reported isolation and identification of neomycin B (**316**) (Figure 19), a 28-membered macrolide containing 19 chiral centers, from *Micromonospora* sp. CNY-_010_ associated with *Stypopodium zonale* collected at the Bahamas Islands. Neaumycin B displayed potent cytotoxicity and showed significant efficacy and selectivity toward U87 human glioblastoma with an LD_50_ value of 5.6 × 10^−5^µg/mL [175].

##### Polyketides Derived from the Green Algae ASSOCIATED Actinomycetes

A new compound of 23-O-butyrylbafilomycin D(**317**), together with bafilomycin D(**320**), 9-hydroxybafilomycin D (**321**), and bafilomycin A1(**322**) (Figure 19), was isolated from seaweed-derived *Streptomyces* sp. HZP-2216E separate from traditional Chinese medicine sea lettuce *Ulva pertusa*. Later, two novel bafilomycins 21,22-en-bafilomycin D (**318**) and 21,22-en-9-hydroxybafilomycin D (**319**) (Figure 19), together with bafilomycin A2 (**S182**) were separated. Compounds **318** and **319** had potent activity against the proliferation of glioma U251 and C6 cells with IC_50_ values of 0.12–1.08 μM. In addition, they were active against MRSA with MIC values of 12.5 mg/mL. The four bafilomycins of compounds **320**–**322** and **317** showed potent activity in suppressing the proliferation of the four tested glioma cell lines with IC_50_ values of 0.35 to 2.95 µM. In addition, compounds **320**–**322** were reported to have antibacterial, antifungal, insecticidal, and herbicidal activities. Bafilomycins D and A1 (**320**, **322**) also exhibited potent activity in inhibiting vacuolar-type ATPase [170,171].

Desertomycin G (**323**) (Figure 19) was separated from *Streptomyces althioticus* MSM3 associated with intertidal macroalgae *Ulva* sp. collected at Cantabrian Sea. Desertomycin G displayed potent antibiotic activities against several clinically relevant pathogens and moderate activity against relevant Gram-negative clinical pathogens. Additionally, it affects the viability of tumor cell lines, such as human breast adenocarcinoma (MCF-7) and colon carcinoma (DLD-1), but not normal mammary fibroblasts [176].

##### Polyketides Derived from the Red Algae-Associated Actinomycetes

Isolation of new α-pyrone polyketides zoumbericins A, B (**S183**, **S184**) and Germicidins K, L (**S185**, **S186**), together with six previously reported metabolites wailupemycin D (**S187**), wailupemycin E (**S188**), enterocin/vulgamycin (**324**), 5-deoxy-enterocin (**325**), germicidin A (**314**) and germicidin B (**315**) (Figure 19), was reported in 2017 from *Streptomyces ambofaciens* BI0048 separated from the red alga *Laurencia glandulifera*. Among them, enterocin showed herbicidal activity and weak antibacterial activity. 5-Deoxy-enterocin was reported to be active against *S. lutea*, *S. aureus*, *Klebsiella pneumoniae,* and *Vibrio percolans*. Germicidin A displayed weak activity against *Streptomyces viridochromogenes* and *Streptomyces griseus* [177].

##### Polyketides Derived from the Cyanobacteria-Associated Actinomycetes

Isolation of antibiotic Bisanthraquinones (**326**–**328**) (Figure 19) was reported from *Streptomyces* sp. N1-78-1 associated with unicellular cyanobacteria parasitized on the tunic surface of *Ecteinascidia turbinate* collected in La Parguera, Puerto Rico. The metabolites **326** and **327** potently inhibited the growth of MRSA. All compounds were moderately active against HCT-116 human colon tumor cells [112].

##### Polyketides Derived from the Actinomycetes Associated with Marine Plants

In 2015, Yong-Fu Huang et al. reported the discovery of a new anthraquinone (**329**) (Figure 19) from *Streptomyces* sp. FX-58 is associated with *Salicornia herbacea* collected in Qingdao, Shandong province, China. Compound **329** showed an inhibitory effect on cancer cell HL-60, BCTC-823 and MDA-MB-435 with IC_50_ values of 6.83, 82.2, 56.59 mg/ mL, respectively [178].

#### 3.2.3. Peptides

Two antifouling diketopiperazines bmDKP (**330**) and imDKP (**331**) (Figure 19) were isolated from *Streptomyces praecox* strain 291-11 separated from the rhizosphere of *Undaria pinnatifida*. The two compounds inhibited zoospores with a therapeutic ratio (LC_50_/EC_50_) of 17.7 and 21 and inhibited diatoms with a therapeutic ratio of 17.7 and 21, respectively [179].

#### 3.2.4. Other Structure Classes

**Figure 20:**
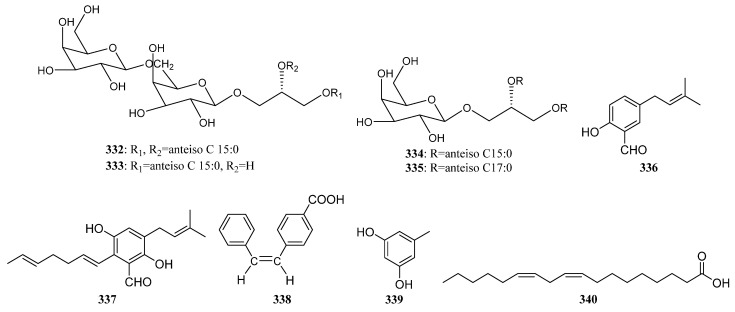
Structures of compounds **332**–**340**.

Two known compounds octadecanoic acid (**S189**) and cholest-4-en-3-one (**S190**) were discovered from *Streptomyces* sp. FX-58 is associated with marine plant *Salicornia herbacea* [178]. Glycoglycerolipids (**332**–**335**) (Figure 20) produced by *Streptomyces coelescens* PK206-15 derived from brown algae *Laminaria japonica* rhizosphere were active against the following fouling animals and plants: zoospores of *Ulva pertusa*, the diatom *Navicula annexa*, the mussel *Mytilus edulis* and fouling bacteria with an EC_50_ range of 0.005–0.2 μg/mL [180].

Isolation of two antibacterial benzaldehydes 2-hydroxy-5-(3-methylbut-2-enyl) benzaldehyde (**336**) and 2-hepta-1,5-dienyl-3,6-dihydroxy-5-(3-methylbut-2-enyl) benzaldehyde (**337**) (Figure 20) were reported from *Streptomyces atrovirens* PK288-21 separated from the rhizosphere of the brown algae *Undaria pinnatifida*. The compound **336** is a new benzaldehyde derivative, and metabolite **337** was the first time reported in the genus *Streptomyces*. The two compounds were active against *Edwardsiella tarda* and *Streptococcus iniae* [181].

Shan-Shan Su et al. reported the discovery of a novel compound (**S191**) in 2013 together with a known compound (**S192**) from lichen-derived *Streptomyces cavourensis* YY01-17 [173]. And a novel bioactive antimicrobial kocumarin (**338**) (Figure 20) was produced by *Kocuria marina* CMG S^2^ associated with brown macroalga *Pelvetia canaliculata* attached to the rocks of Sonmiani Beach and demonstrated prominent and rapid growth inhibition against all tested fungi and pathogenic bacteria [182]. Three known compounds benzoic acid (**S158**), hydrocinnamic acid (**S193**), and (*E*)-cinnamic acid(**S194**) were separated from *Streptomyces ambofaciens* BI0048 associated with red alga *Laurencia glandulifera* [177].

Geosmin (**S195**), the compound responsible for the “earth smell” and beta-patchoulene (**S196**) used as a fragrance agent in the perfume industry, were two major metabolites of *Streptomyces carnosus* M-40 [168]. 5-methylresorcinol (**339**) and Linoleic acid (**340**) (Figure 20), which were isolated from *Nocardiopsis* sp. AS23C associated with brown alga *Sargassum arnaudianum* exhibited antibacterial activity against the Gram-positive *Staphylococcus aureus*, *Bacillus subtilis* ATCC6051, and *Streptomyces viridochromogenes* Tü 57 [169].

### 3.3. Data Analysis of the Secondary Metabolites from Actinomycetes Associated to Various Hosts

**Figure 21:**
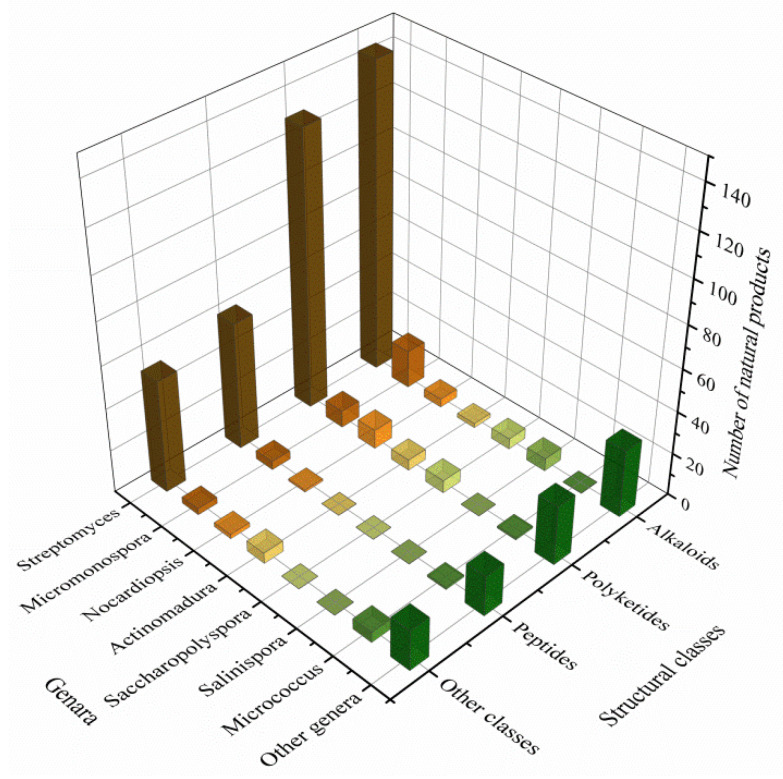
The structural distribution of metabolites from the actinomycetes is divided by genera.

**Figure 22:**
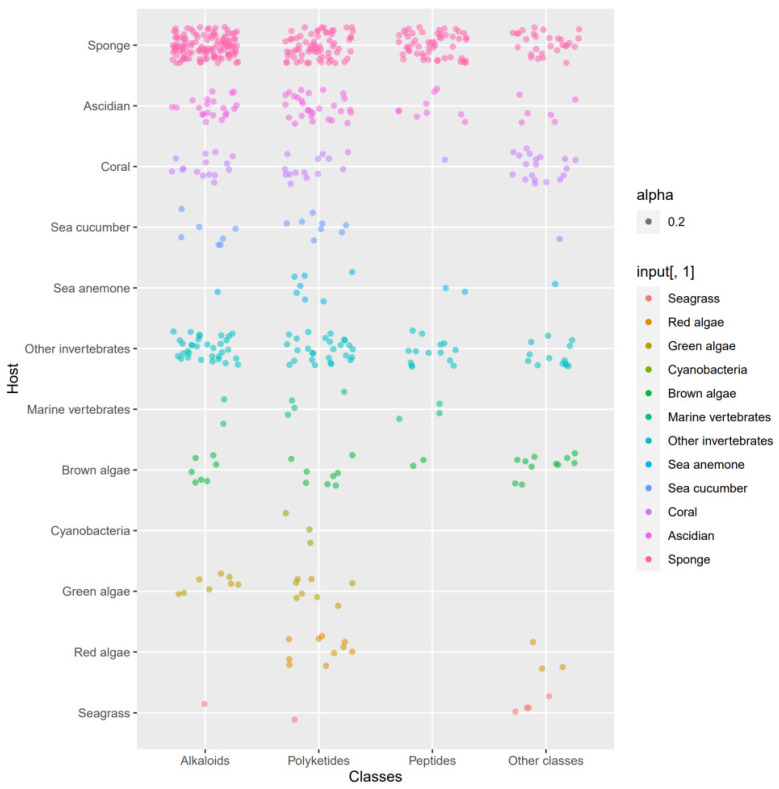
The structural distribution of metabolites from -actinomycetes associated with various hosts. The dots in that figure represent the number of compounds and the color darkens when the dots overlap.

**Figure 23:**
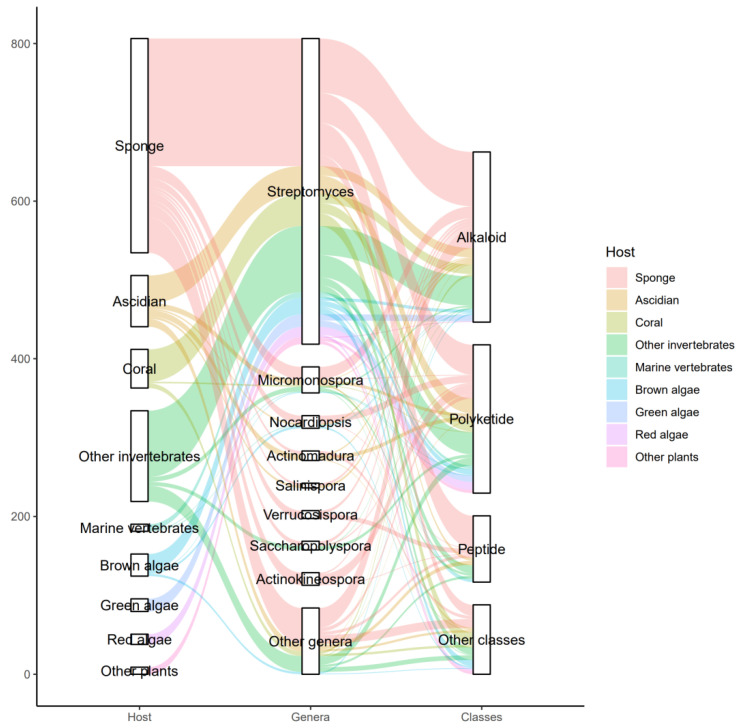
The distribution of secondary metabolites produced by actinomycetes with various genera derived from different hosts. The width of the extended branches in the figure corresponds to the number of secondary metabolites.

**Figure 24:**
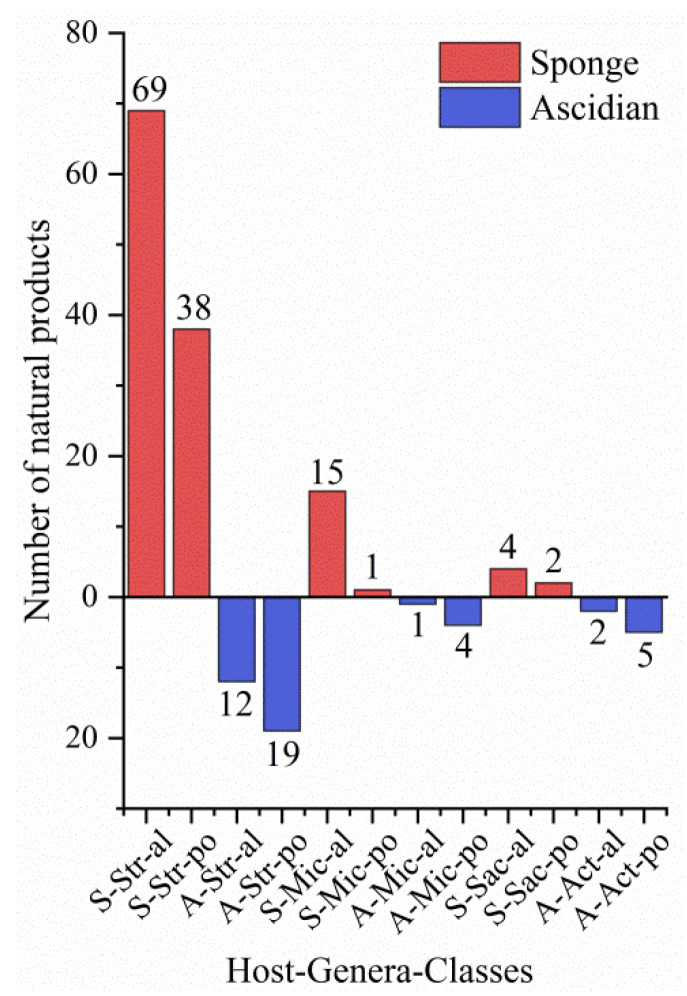
The structural distribution of metabolites from three dominant actinomycetes in the main host’s sponge and ascidian. The x-axis labels represent the host-genus-structure classes of actinomycetes: (A) Ascidian; (S) Sponge; (Str) Streptomyces; (Mic) *Micromonospora*; (Sac) Saccharopolyspora; (Act) Actinomadura; (al) alkaloid; (po) polyketide.

A total of 536 metabolites have been discovered from 155 actinomycetes associated with various marine hosts belonging to 22 genera. Among them, alkaloids (37%), polyketides (33%), and peptides (15%) comprise the largest proportion of secondary metabolites, while *Streptomyces* (68%), *Micromonospora* (6%), and *Nocardiopsis* (3%) are the dominant producers (Figure 21). Figure 22 showed the distribution of secondary metabolites currently identified in different host-related actinomycetes. The majority of the secondary metabolites were isolated from the actinomycetes associated with sponges (47%), ascidians (11%), and corals (9%), as well as brown algae (5%). Furthermore, the Sankey diagram and histogram were done to show the distribution of secondary metabolites produced by actinomycetes with various genera derived from different hosts, which is convenient for readers to have an overall understanding of the current secondary metabolites from marine organism-associated actinomycetes (Figure 23 and Figure 24).

**Figure 25:**
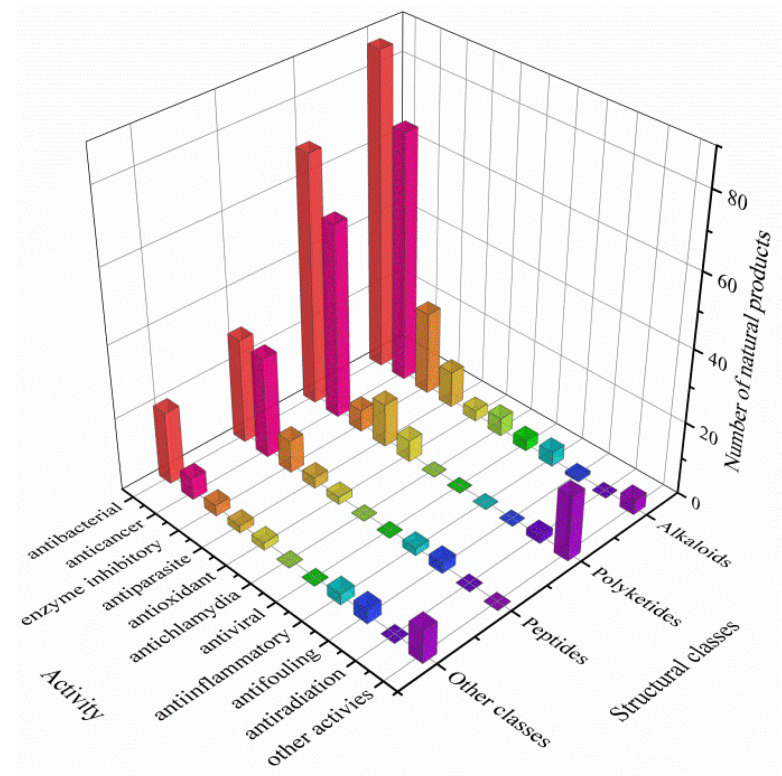
The diversity distribution of biological activity with different structures.

Approximately 64% of the SMs displayed various biological activities, especially antimicrobial activity and cytotoxicity (Figure 25). Interestingly, some of these active metabolites with multiple biological properties deserve more attention (Table S7); for example, metabolites with cytotoxicity usually have antibacterial or antiparasitic activities, and some metabolites showed antibacterial activity can also act as enzyme inhibitors. This study advances the knowledge of these actinomycetes in respect to the metabolic potential of medicinal lead compounds.

### 3.4. Clinical Information of the Secondary Metabolites

Molecules with excellent activities, which have been in clinical applications or have entered clinical trials, were listed in Table 1. For example, rifamycin SV (**9**) is the earliest rifamycin antibiotic used in clinical application. Tetrodotoxin (**105**) has been widely used as an analgesic, sedative, antispasmodic, and local anesthetic in clinics. And daunomycin (**307**) has a good effect on acute myeloid leukemia. In addition, for these drugs already in clinical use, more clinical trials are underway for new diseases or new usages. (More detailed information can be found on the website ClinicalTrials.gov accessed on 25 October 2021).

## 4. Discussion

Metabolites from actinomycetes associated with marine organisms have proven to be an abundant source for the isolation of multiple potent bioactive metabolites with diverse structures. In this review, we attempt to discuss the significance of the special ecological status and genetic factors of these actinomycetes with multiple hosts. The chemical ecology underlying hosts–actinomycetes interactions provide a great opportunity for the discovery of novel drugs. During the co-evolution, these actinomycetes and their specific hosts constructed a coordinated and relatively independent micro-ecological environment, in which SMs can be tolerated by the host and are the active inhibiting specific external invasion. Therefore, actinomycetes associated with various marine hosts play an important ecological role in producing novel medicinal active compounds. Currently, there are relatively few studies on these actinomycetes, but many secondary metabolites have been isolated with excellent bioactivities. Some of these metabolites have been used in clinical applications or have entered clinical trials where they are expected to become new drugs. There is no doubt that further exploration can be a useful strategy for discovering novel marine natural products. These actinomycetes, however, are difficult to be cultured under experimental conditions. Therefore, in-depth exploration of the ecology of these actinomycetes to continuously optimize culture conditions is crucial for further research. Meanwhile, the use of advanced bioinformatics technology for gene detection of uncultured actinomycetes and heterologous expression of the collected biosynthetic gene clusters will be another important pathway for research on SMs of marine organism-associated actinomycetes.

## Figures and Tables

**Table 1:** Clinical information of the secondary metabolites.

Compounds	Study Title	Conditions	Related Compounds for Interventions	Phase	NCT Number
Rifamycin SV (**9**)	Rifamycin SV-MMX^®^ 400 mg b.i.d. vs. Rifamycin SV-MMX^®^ 600 mg t.i.d. vs. Placebo in Acute Uncomplicated Diverticulitis	Uncomplicated Diverticulitis	Rifamycin SV-MMX^®^	Phase 2	NCT01847664
	Rifamycin SV-MMX^®^ 600 mg Tablets Administered Three or Two Times Daily to Patients With IBS-D	Diarrhea-predominant Irritable Bowel Syndrome	Rifamycin SV	Phase 2	NCT03099785
	Study to Evaluate Safety and Efficacy of Rifamycin SV Multi-Matrix System (MMX) for the Treatment of Traveler’s Diarrhea (TD)	Traveler’s Diarrhea	Rifamycin SV MMX	Phase 3	NCT01142089
	Rifamycin SV-MMX^®^ Tablets Versus Ciprofloxacin Capsules in Acute Traveller’s Diarrhoea	Traveler’s Diarrhea	Rifamycin SV-MMX^®^	Phase 3	NCT01208922
Diazepinomicin (**28**)	A Phase I Study of ECO-4601 in Patients With Advanced Cancer	Tumors Glioma Colorectal Cancer	ECO-4601	Phase 1	NCT00338026
	Efficacy Study of TLN-4601 in Patients With Recurring Glioblastoma Multiforme	Glioblastoma Multiforme	TLN-4601	Phase 2	NCT00730262
Staurosporine (**3**)	A Phase I Trial of Continuous Infusion UCN-01 in Patients With Refractory Neoplasms	Breast Cancer Lymphoma Neoplasm Prostatic Neoplasm	7-hydroxystaurosporine (UCN-01)	Phase 1	NCT00001444
	PK and Safety of Midostaurin in Subjects With Impaired Hepatic Function and Subjects With Normal Hepatic Function	Hepatic Impairment	Midostaurin	Phase 1	NCT01429337
	Phase I Combination of Midostaurin, Bortezomib, and Chemo in Relapsed/Refractory Acute Myeloid Leukemia	Acute Myeloid Leukemia AML With Multilineage Dysplasia Following Myelodysplastic Syndrome	Midostaurin	Phase 1	NCT01174888
	Azacitidine With or Without Nivolumab or Midostaurin, or Decitabine and Cytarabine Alone in Treating Older Patients With Newly Diagnosed Acute Myeloid Leukemia or High-Risk Myelodysplastic Syndrome	Acute Myeloid Leukemia Myelodysplastic Syndrome Myelodysplastic Syndrome With Excess Blasts-2	Midostaurin	Phase 2 Phase 3	NCT03092674
Tetrodotoxin (**105**)	Tetrodotoxin Open-label Efficacy and Safety Continuation Study	Pain Cancer	Tetrodotoxin	Phase 3	NCT00726011
	Safety & Efficacy Study of Subcutaneous Tetrodotoxin for Moderate to Severe Inadequately Controlled Cancer-related Pain	Pain Cancer	Tetrodotoxin	Phase 3	NCT00725114
Daunomycin (**307**)	Pilot Study Efficacy and Tolerance Fish Oil Emulsion Daunorubicin and Cytarabine Treatment of AML Younger Patients	Acute Myeloid Leukemia (AML)	Daunorubicin	Phase 2	NCT01999413
	A Randomized Study of Gemtuzumab Ozogamicin (GO) With Daunorubicine and Cytarabine in Untreated Acute Myeloid Leukemia (AML) Aged of 50–70 Years Old	Acute Myeloid Leukemia	Daunorubicin	Phase 3	NCT00927498
Linoleic acid (**340**)	Proof of Principle Trial to Determine if Nutritional Supplement Conjugated Linoleic Acid (CLA) Can Modulate the Lipogenic Pathway in Breast Cancer Tissue	Breast Cancer	Conjugated Linoleic Acid (CLA)	Early Phase 1	NCT00908791
	Conjugated Linoleic Acid / Leucine Versus Metformin on Visceral Fat in Metabolic Syndrome	Metabolic Syndrome	Conjugated linoleic acid/Leucine	Phase 2	NCT02629627
	Conjugated Linoleic Acid and Atherosclerosis	Atherosclerosis	Cis9, trans11 conjugated linoleic acid	Phase 3	NCT00706745
Actinomycin D (**251**)	Dactinomycin in Treating Patients With Persistent or Recurrent Gestational Trophoblastic Neoplasia	Gestational Trophoblastic Tumor	Dactinomycin	Phase 2	NCT00003688
	Addition of Ipilimumab (MDX-010) To Isolated Limb Infusion (ILI) With Standard Melphalan and Dactinomycin In The Treatment of Advanced Unresectable Melanoma of The Extremity	Melanoma	Dactinomycin	Phase 2	NCT01323517
	Methotrexate Compared With Dactinomycin in Treating Patients With Gestational Trophoblastic Neoplasia	Gestational Trophoblastic Neoplasia	Dactinomycin	Phase 3	NCT00003702

## Data Availability

Data is contained within the article or Supplementary Materials.

## Supplementary Materials

The following are available online at https://www.mdpi.com/article/10.3390/md19110629/s1, Figures S1–S19: structures of compounds **S1**–**S196**, Figure S20**:** original rectangular (Neighbor-joining) tree. Table S1: actinomycetes and their accession number of Figure 1. Tables S2–S6: the data of Figure 2, Figure 3, Figure 4, Figure 5 and Figure 6. Table S7: The summary of all secondary metabolites including information on separation sources, structural types, and biological activities. Table S8: Summarized repetitive compounds identified from multiple actinomycetes.
