# Supplementary material for: Natural Products from Actinomycetes Associated with Marine Organisms
Source: Mar Drugs. 2021 Nov 10;19(11):629. doi: 10.3390/md19110629 (PMC8621598; doi:10.3390/md19110629)
Supplement: Supplementary file 1 [file marinedrugs-19-00629-s001.zip › marinedrugs-1437248-SI.pdf]

Supporting information

Natural products from actinomycetes associated with marine animals and plants

Jianing Chen [1], Lin Xu [1], Yanrong Zhou [1] and Bingnan Han [1,*]

1 Department of Development Technology of Marine Resources, College of Life Sciences and Medicine, Zhejiang Sci-Tech University, Hangzhou 310018, China; 202020801006@mails.zstu.edu.cn. (J.C); linxu@zstu.edu.cn. (L.X.); zhouyanrong@zstu.edu.cn (Y.Z)

* Correspondence: hanbingnan@zstu.edu.cn; Tel.: +86-571-8684-3303

Content

1. Structures of inactive metabolites listed 4

Figure S1. Alkaloids derived from the sponge-associated actinomycetes. 5

Figure S2. Alkaloids derived from the coral-associated actinomycetes. 6

Figure S3. Alkaloids derived from the ascidian-associated actinomycetes. 6

Figure S4. Alkaloids derived from the actinomycetes associated with other marine animals 7

Figure S5. Polyketides derived from the sponge-associated actinomycetes. 8

Figure S6. Polyketides derived from the coral-associated actinomycetes. 9

Figure S7. Polyketides derived from the ascidian-associated actinomycetes. 9

Figure S8. Polyketides derived from the actinomycetes associated with other marine animals. 10

Figure S9. Peptides derived from the sponge-associated actinomycetes. 11

Figure S10. Peptides derived from the coral-associated actinomycetes. 12

Figure S11. Peptides derived from the actinomycetes associated with other marine animals. 13

Figure S12. Terpenoids derived from the actinomycetes associated with marine animals. 13

Figure S13. Steroids derived from the actinomycetes associated with marine animals. 13

Figure S14. Other classes metabolites derived from the actinomycetes associated with marine animals. 16

Figure S15. Alkaloids derived from the green algae-associated actinomycetes. 16

Figure S16. Alkaloids derived from the lichen-associated actinomycetes. 17

Figure S17. Polyketides derived from the brown algae-associated actinomycetes. 17

Figure S18. Polyketides derived from the red algae-associated actinomycetes.. 18

Figure S19. Other classes metabolites derived from the actinomycetes associated with marine plants, macroalgae and lichens. 18

Figure S20. Original Neighbor-Joining phylogenetic tree of natural product-producing actinomycetes associated to various marine hosts.. 20

2 Data analysis 18

2.1 Data analysis of **Figure 1.** 19

2.2 Data analysis of **Figure 21.** 23

2.3 Data analysis of **Figure 22.** 23

2.4 Data analysis of Figure **23.** 24

2.5 Data analysis of Figure **24.** 27

2.6 Data analysis of Figure **25.** 27

3. Table S1. The actinomycetes and their accession number of Figure 1.. 21

4. Table S2. The data of Figure 21.. 23

5. Table S3. The data of Figure 22.. 23

6. Table S4. The data of Figure 23. 24

7. Table S5. The data of Figure 24.. 27

8. Table S6. The data of Figure 25.. 27

9. Table S7. The summary of all secondary metabolites including information on separation sources, structural types, and biological activities. 28

10. Table S8. Summarized repetitive compounds identified from multiple actinomycetes. 48

1. Structures of inactive metabolites listed

A total of 536 metabolites have been reviewed in this article, in which the structures of 340 compounds with various biological activities are listed in the manuscript, while the remaining structures of inactive metabolites are listed in the supporting information. These chemical structures were drawn by ChemBioDraw Ultra 14.0.

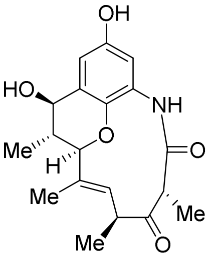

S1

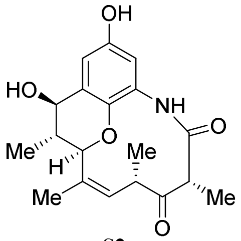

S2

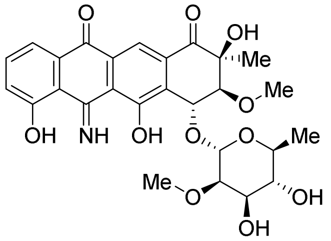

S3

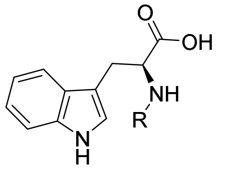

S4 R= 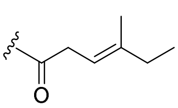

S5 R= 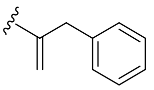

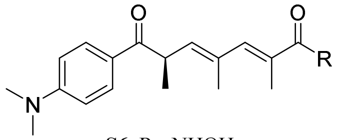

S6: R=-NHOH
S7: R=-OH

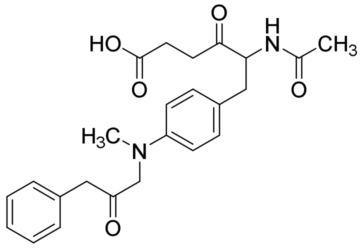

S8

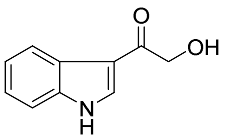

S9

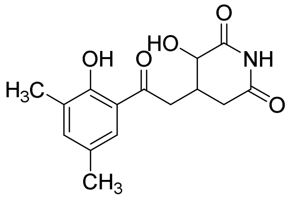

S10

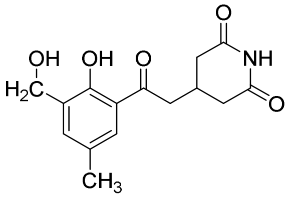

S11

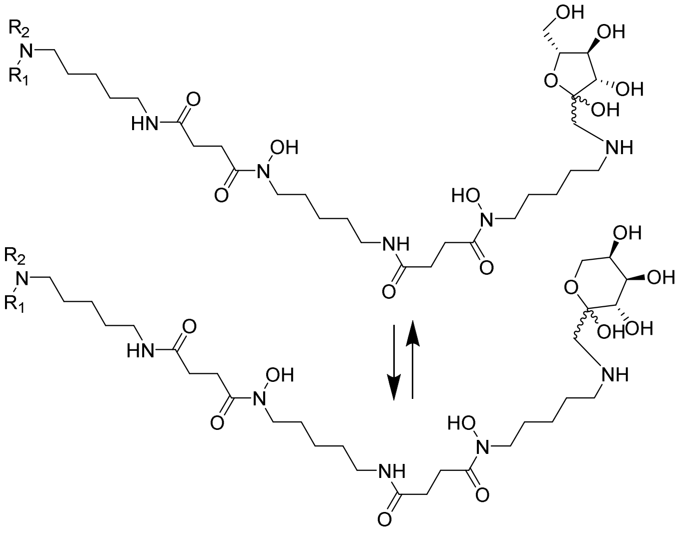

S12 R1=OH, R2= 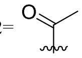

S13 R1=H, R2= 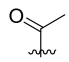

S14 R1=OH, R2= 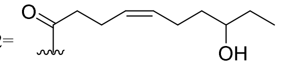

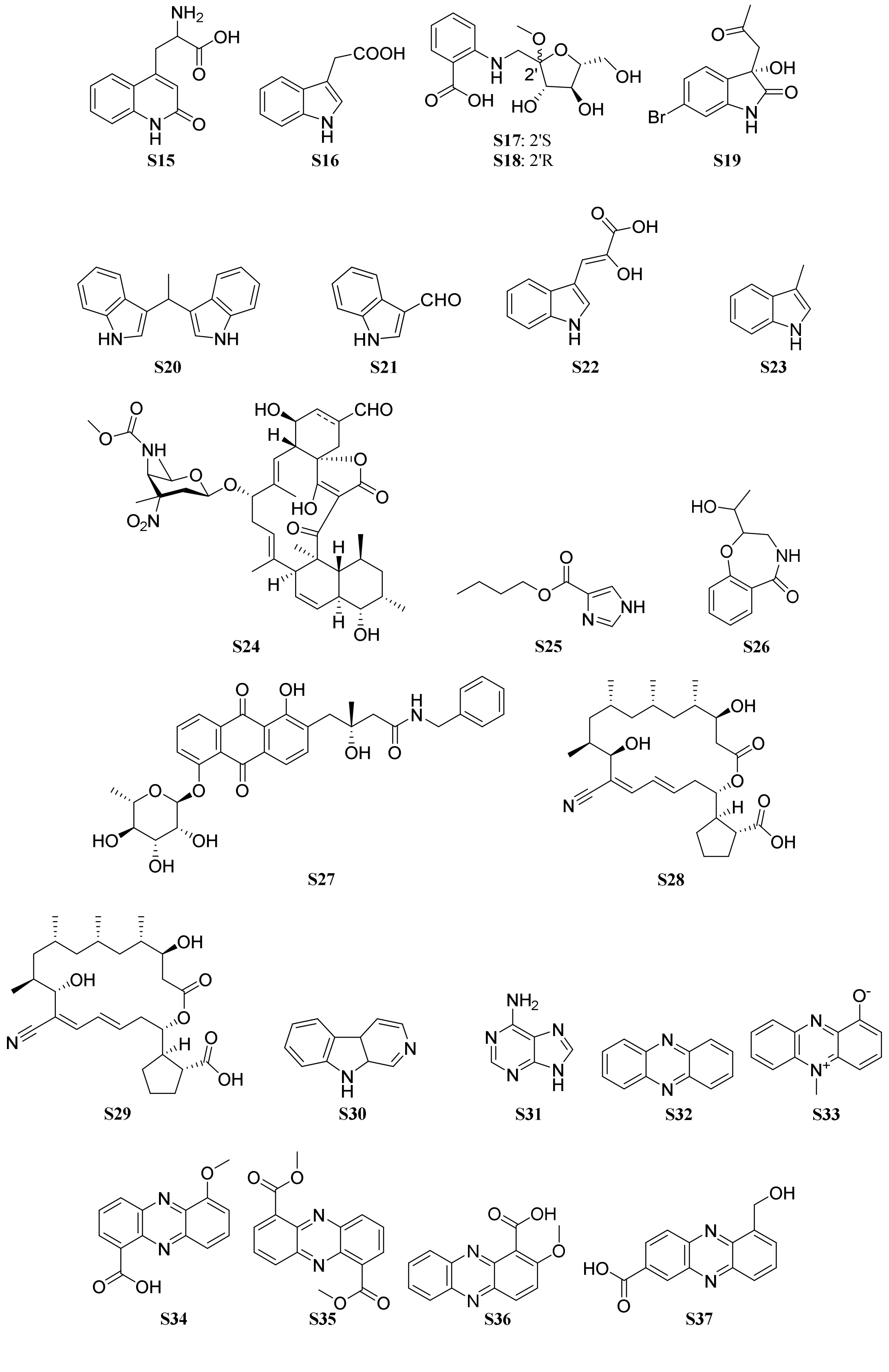

Figure S1. Alkaloids derived from the sponge-associated actinomycetes.

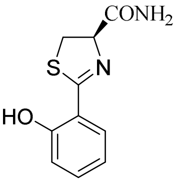

S38

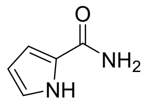

S39

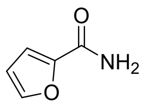

S40

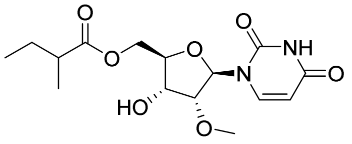

S42

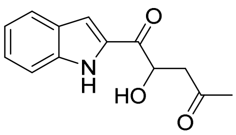

S43

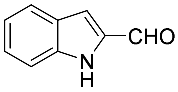

S44

Figure S2. Alkaloids derived from the coral-associated actinomycetes.

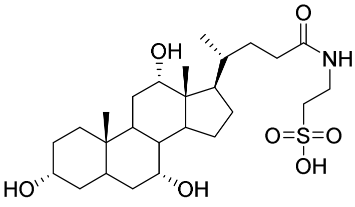

S45

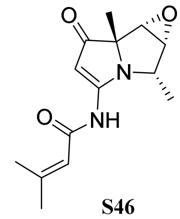

S46

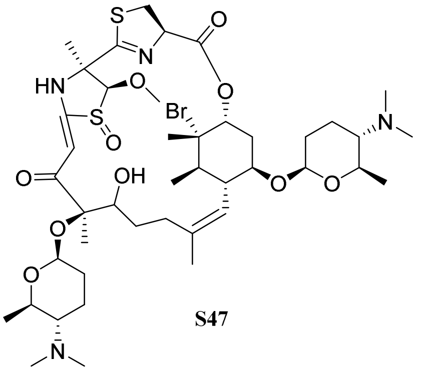

S47

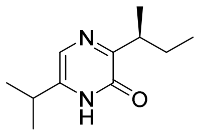

S48

Figure S3. Alkaloids derived from the ascidian-associated actinomycetes.

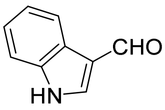

S21

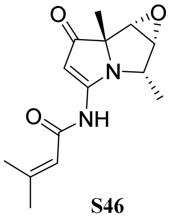

S46

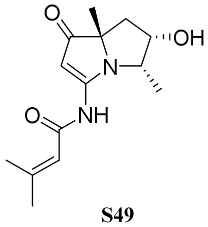

S49

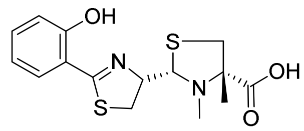

S50

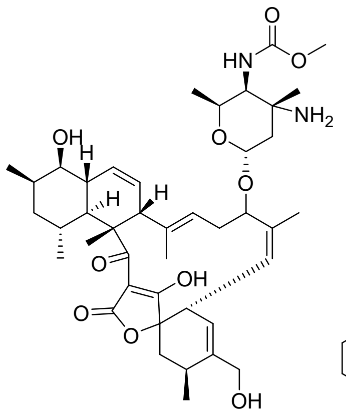

S51

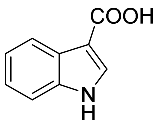

S52

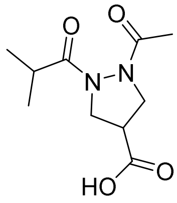

S53

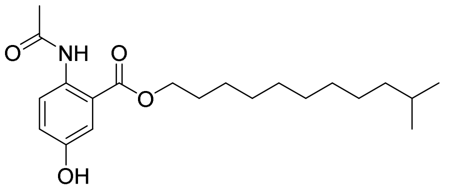

S54

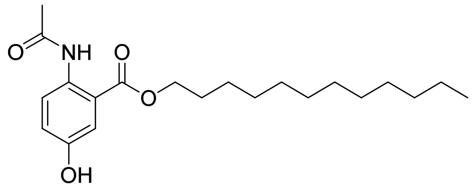

S55

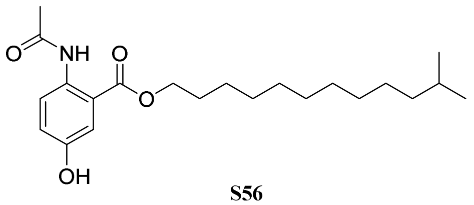

S56

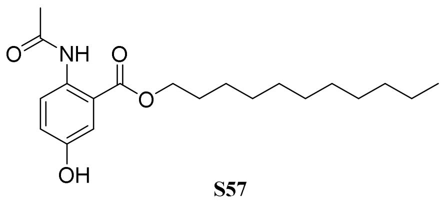

S57

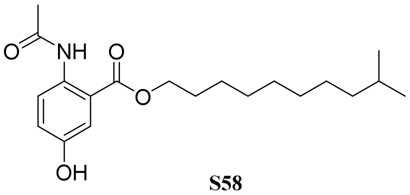

S58

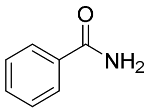

S59

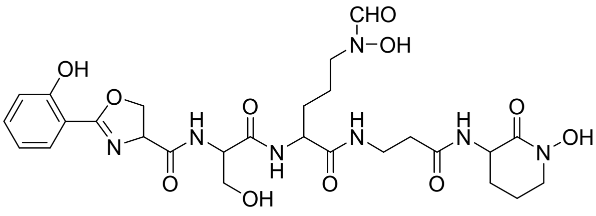

S60

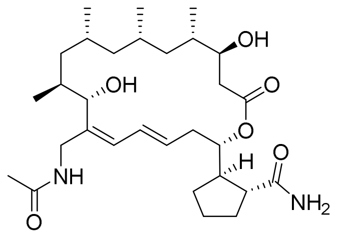

S61

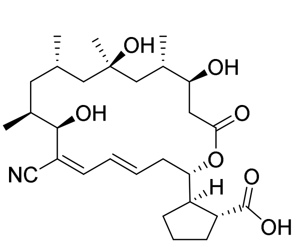

S62

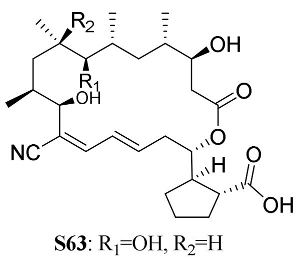

S63: R_1=OH, R_2=H
S64: R_1=H, R_2=OH

Figure S4. Alkaloids derived from the actinomycetes associated with other marine animals.

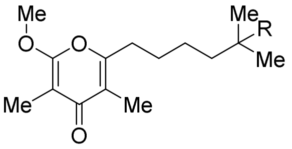

S65: R=OH
S66: R=H

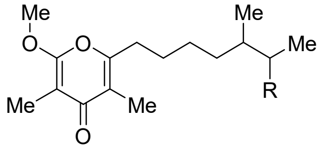

S67: R=OH
S68: R= =O

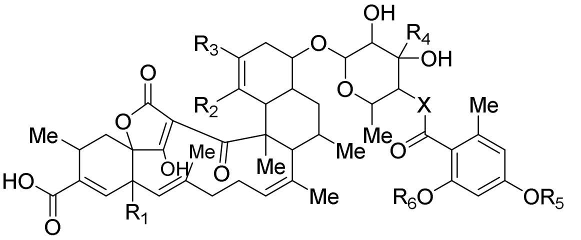

S69: R_1=R_3=R_4=R_5=Me, R_2=R_6=H, X=NH
S70: R_1=R_3=R_5=R_6=Me, R_2=R_4=H, X=O

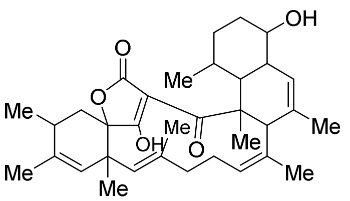

S71

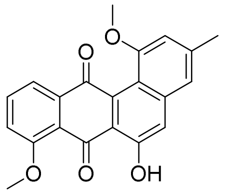

S72

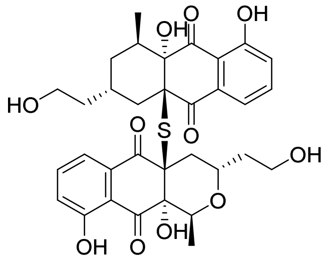

S73

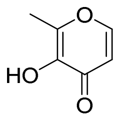

S74

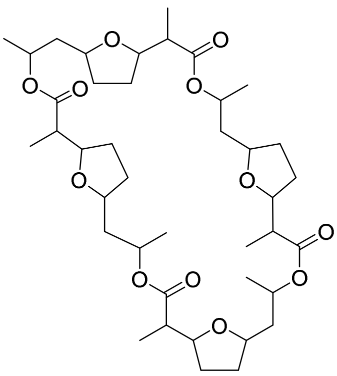

S75

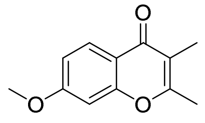

S76

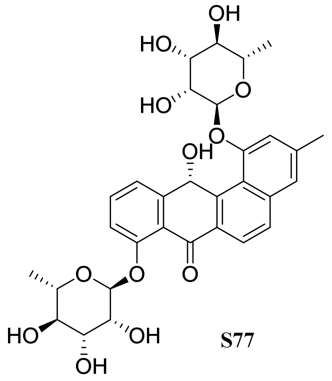

S77

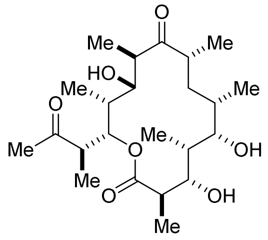

S78

Figure S5. Polyketides derived from the sponge-associated actinomycetes.

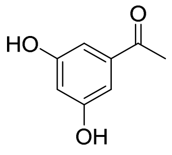

S41

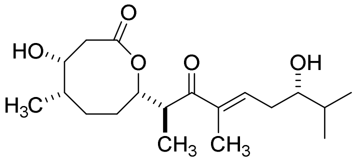

S79

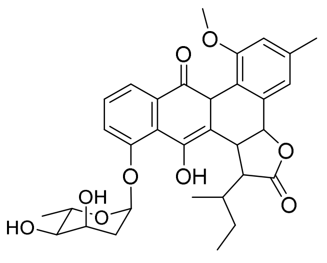

S80

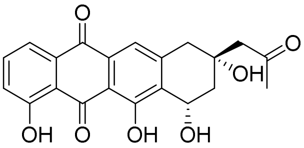

S81

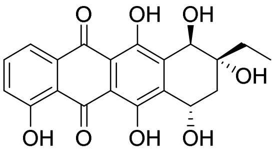

S82

Figure S6. Polyketides derived from the coral-associated actinomycetes.

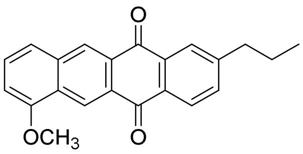

S83

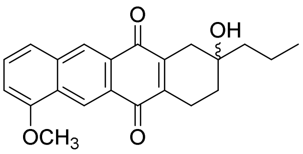

S84

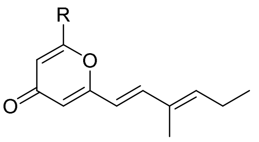

S85: R=CCl_3
S86: R=$CHCl_2$

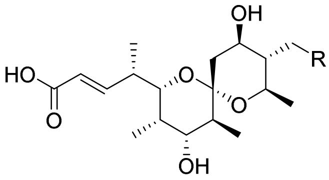

S87 R=Me
S88 R=H

Figure S7. Polyketides derived from the ascidian-associated actinomycetes.

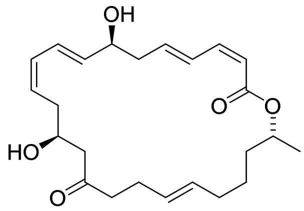

S89

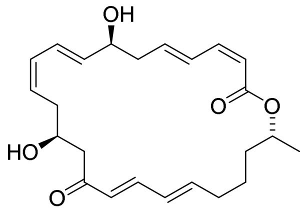

S90

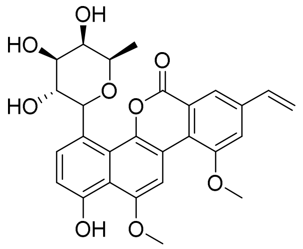

S91

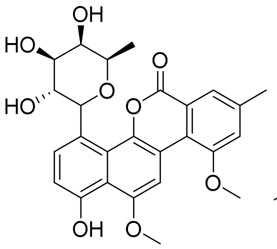
S92

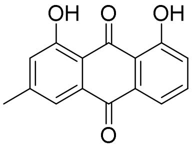
S93

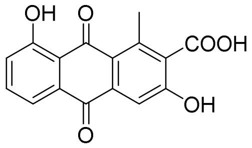
S94

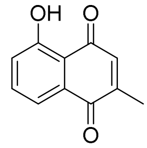
S95

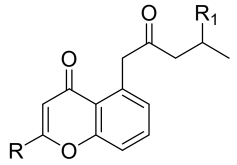
S96: R=CH_3, R_1=α -OH
S96a: R=CH_2OH, R_1=β -OH

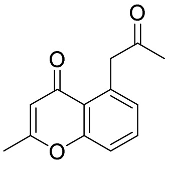
S97

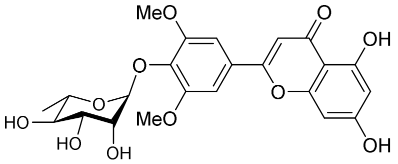
S98

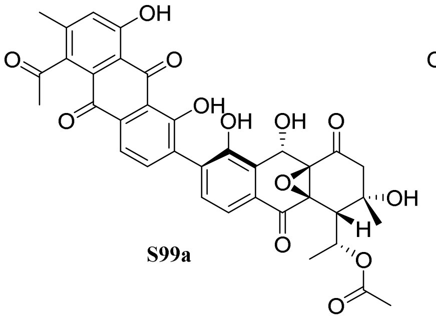
S99a

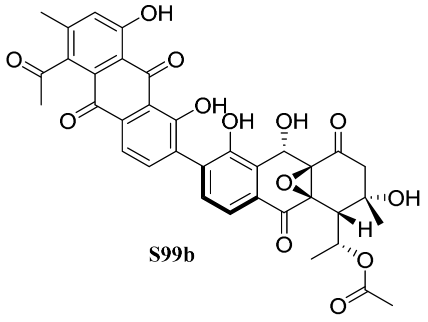
S99b

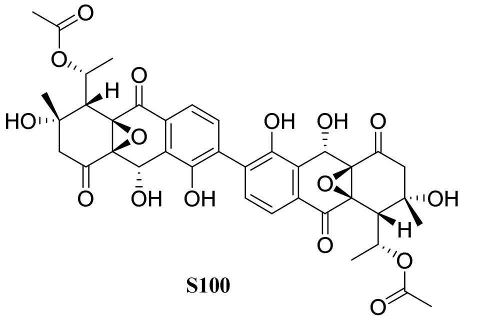
S100

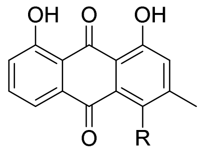
S101: R=H
S102: R=$COCH_3$
S103: R=OH

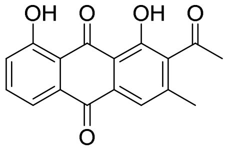
S104

Figure S8. Polyketides derived from the actinomycetes associated with other marine animals.

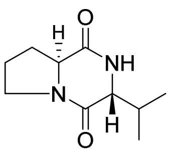
S105

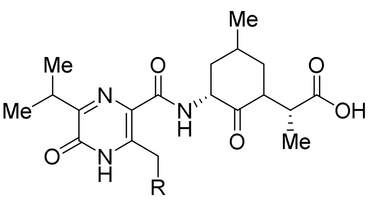
S106: R=Me; **S107**: R=H

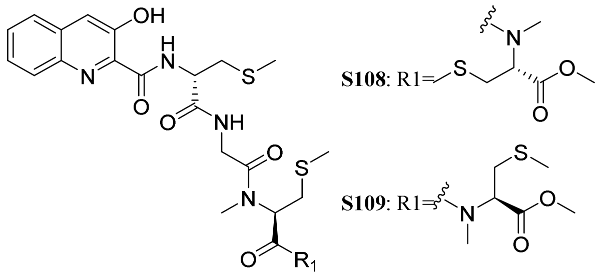

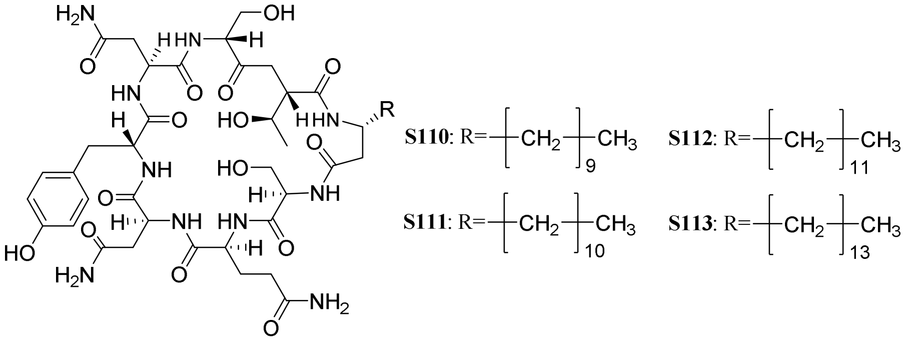

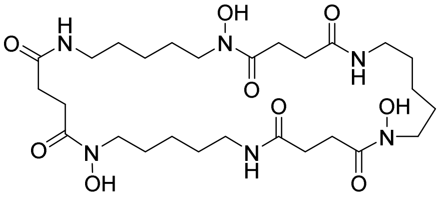

S114

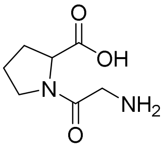

S115

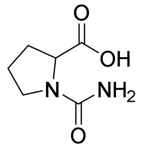

S116

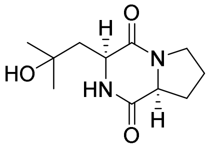

S117

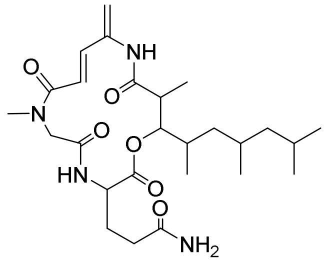

S118

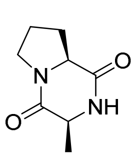

S119

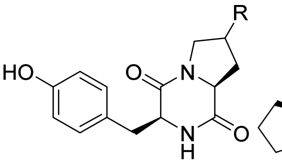

S120: R=H
S121: R=OH

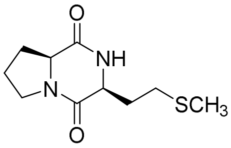

S122

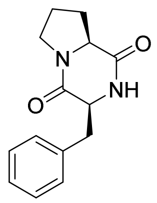

S123

Figure S9. Peptides derived from the sponge-associated actinomycetes.

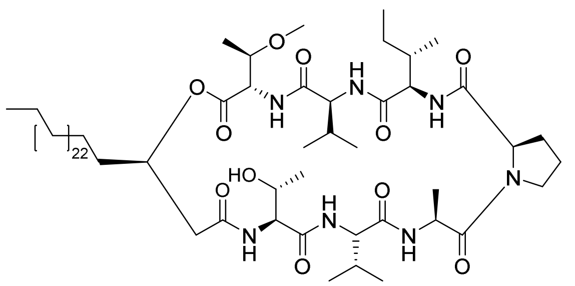

S124

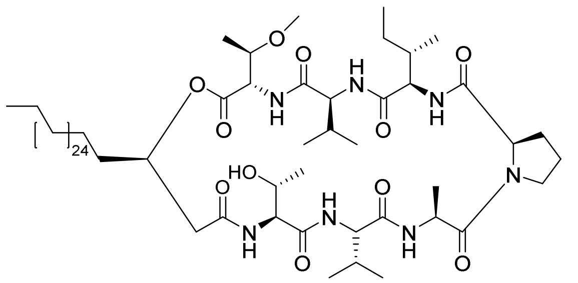

S125

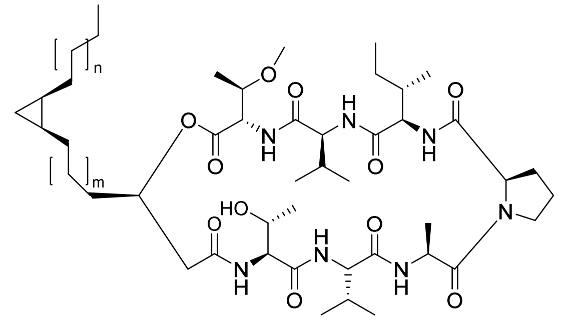

S126

Figure S10. Peptides derived from the coral-associated actinomycetes.

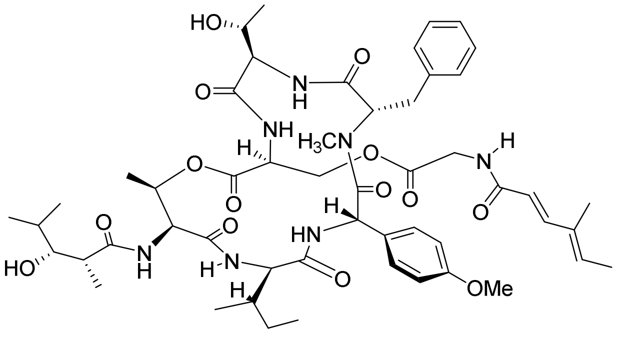

S127

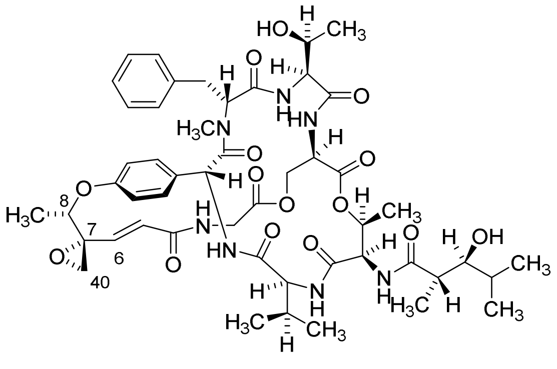

S128

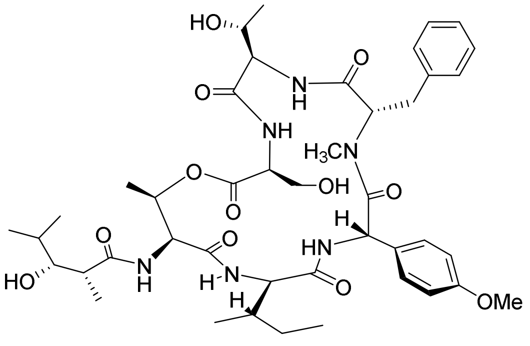

S129

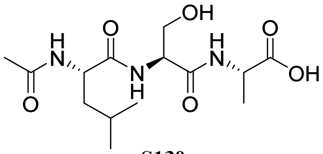

S130

Figure S11. Peptides derived from the actinomycetes associated with other marine animals.

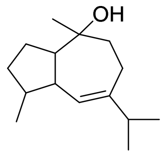

S131

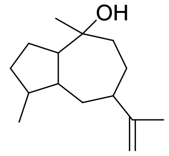

S132

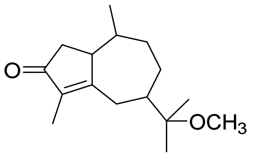

S133

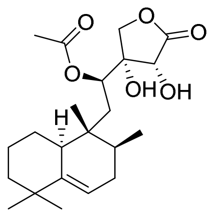

S134

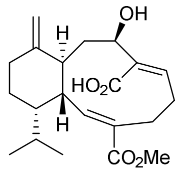

S135

Figure S12. Terpenoids derived from the actinomycetes associated with marine animals.

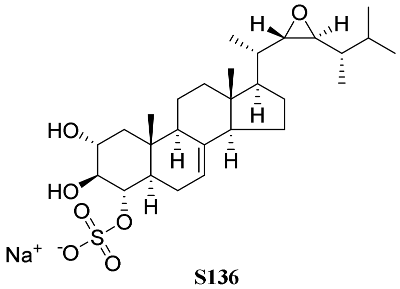

S136

Figure S13. Steroids derived from the actinomycetes associated with marine animals.

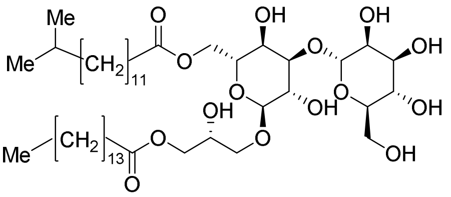

S137

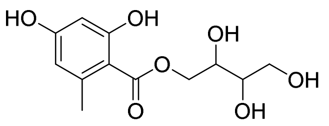

S138

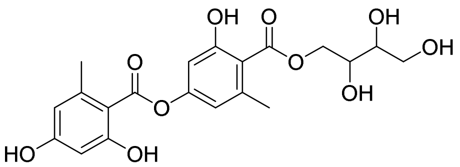

S139

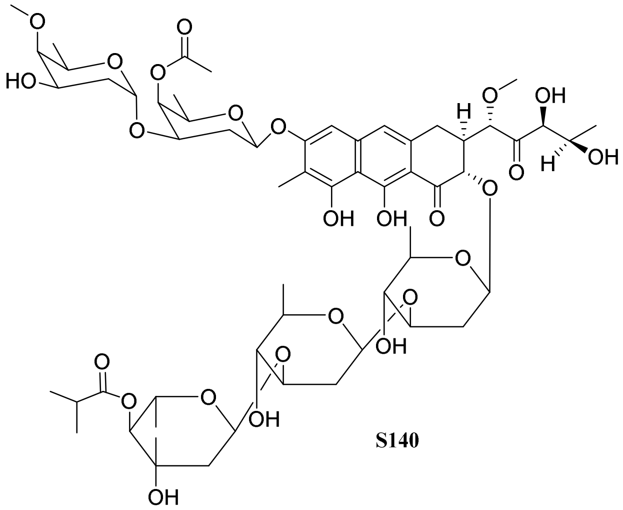

S140

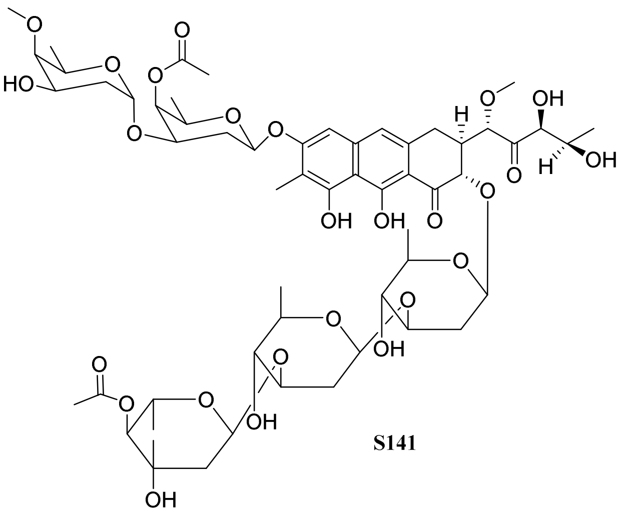

S141

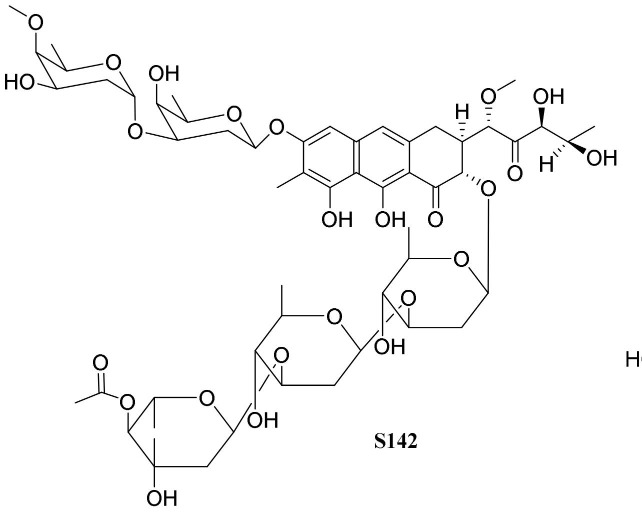

S142

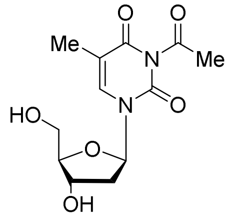

S143

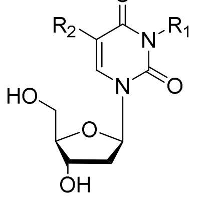

S144: R_1=Me, R_2=Me
S145: R_1=Me, R_2=H

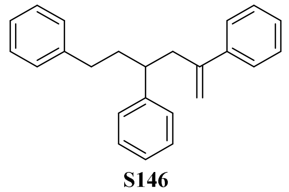

S146

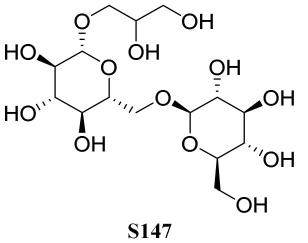

S147

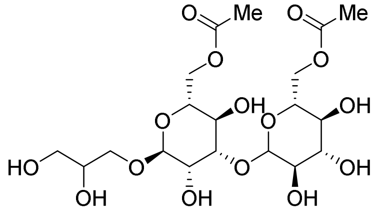

S148

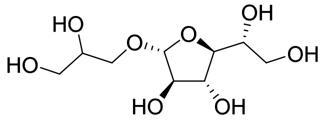

S149

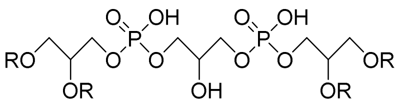

S150 R=anteiso - C15:0 and - C17:0; but also iso - C16:0

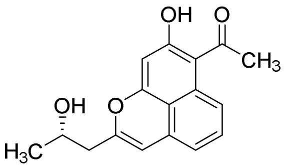

S151

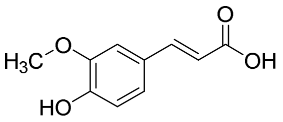

S152

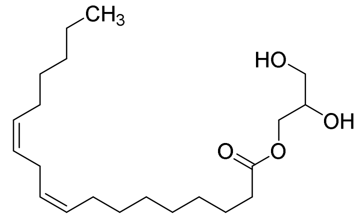

S153

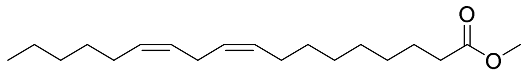

S154

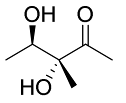

S155a

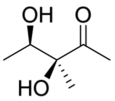

S155b

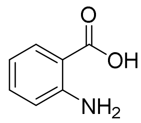

S156

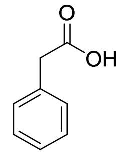

S157

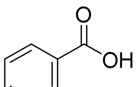

S158

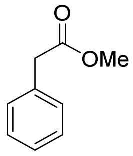

S159

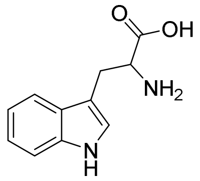

S160

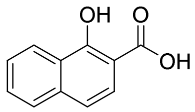

S161

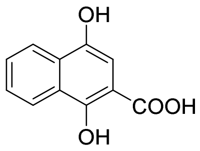

S162

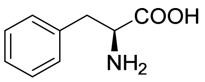

S163

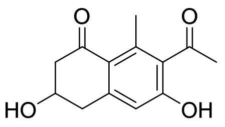

S164

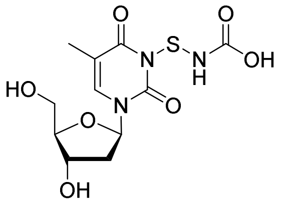

S165

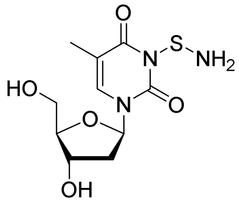

S166

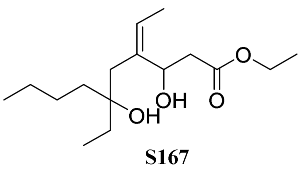

S167

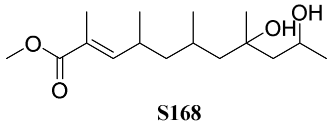

S168

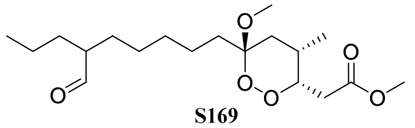

S169

Figure S14. Other classes metabolites derived from the actinomycetes associated with marine animals.

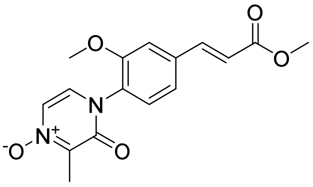

S170

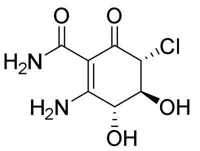

S171

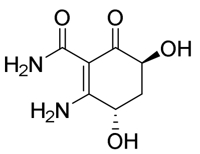

S172

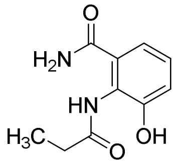

S173

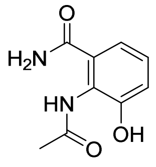

S174

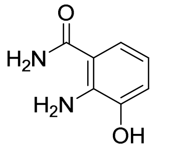

S175

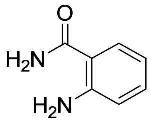

S176

Figure S15. Alkaloids derived from the green algae-associated actinomycetes.

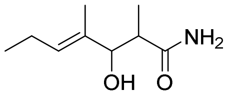

S177

Figure S16. Alkaloids derived from the lichen-associated actinomycetes.

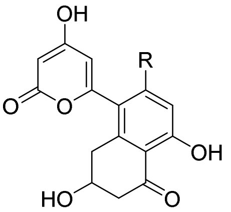

S178: R=n-propyl

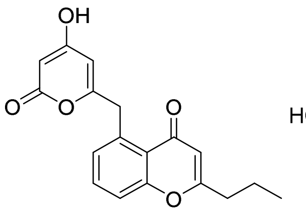

S179

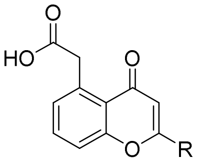

S180: R=n-propyl

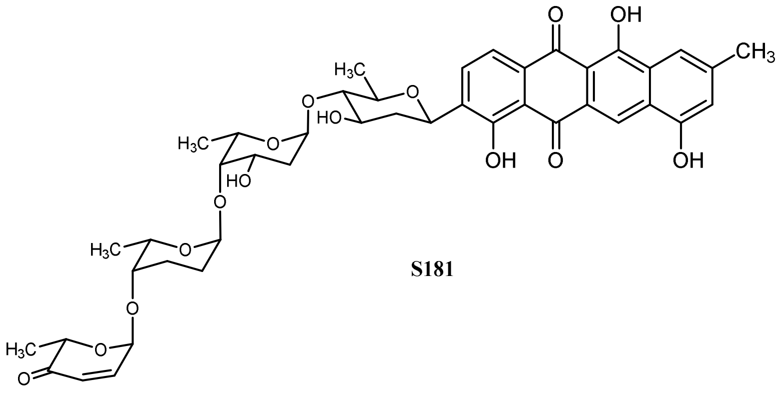

S181

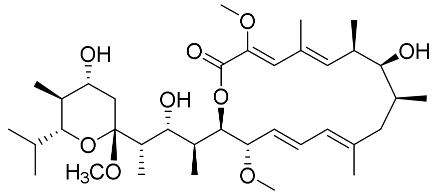

S182

Figure S17. Polyketides derived from the brown algae-associated actinomycetes.

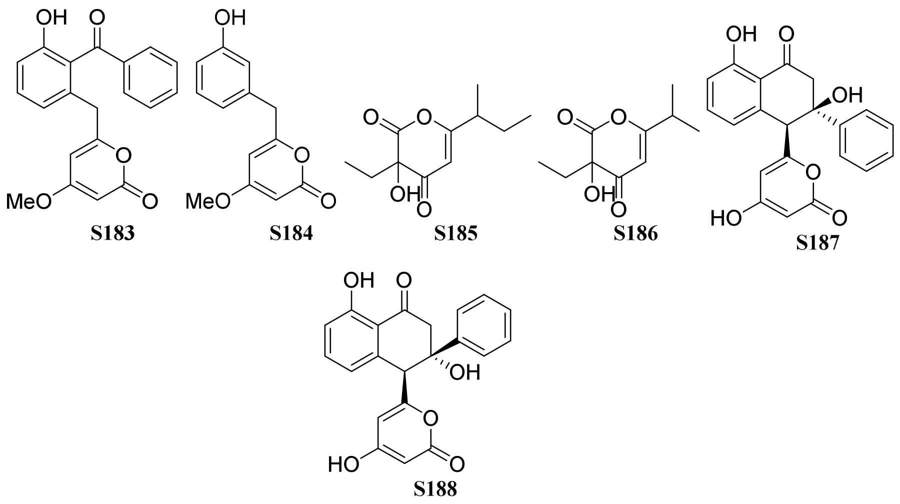

Figure S18. Polyketides derived from the red algae-associated actinomycetes.

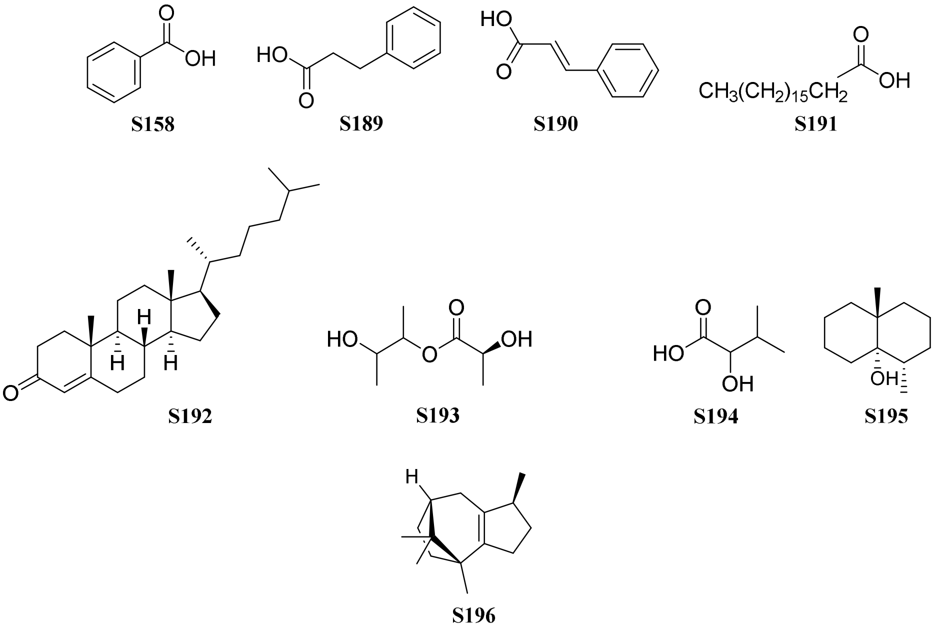

Figure S19. Other classes metabolites derived from the actinomycetes associated with marine plants, macroalgae and lichens.

2 Data analysis

The data were counted according to the corresponding information in the references

(**Tables S2-S6**). And these tables were done by using Microsoft Office Excel 2007 and Microsoft Office Word 2007.

2.1 Data analysis of **Figure 1.**

Eighty-four available product-producing actinomycetes 16S rRNA sequences were obtained from the NCBI GenBank database in September 2021 (**Table S1**). 16S rRNA sequences were aligned by MEGA 7 software with the command of "Align by ClustalW". The phylogenetic tree was visualized using the neighbor-joining method with the Bootstrap setting as 10,000 replicates. Bar, 0.020 substitutions per nucleotide position (**Figure S20**). The original tree was saved as Newick format and imported into Interactive Tree Of Life web server (https://itol.embl.de/) for beautification (**Figure 1**).

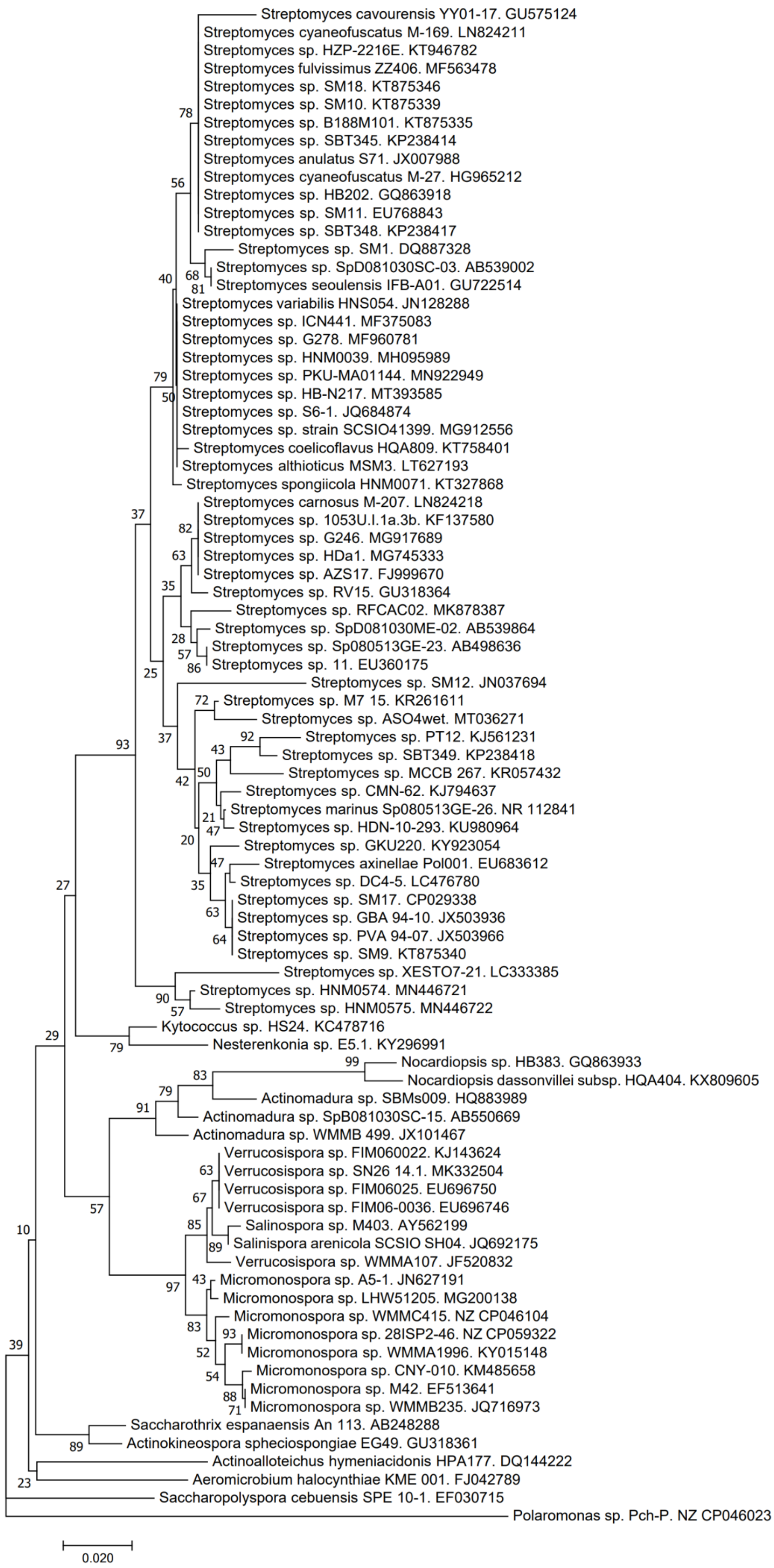

Figure S20. Original Neighbor-Joining phylogenetic tree of natural product-producing actinomycetes associated to various marine hosts.

Table S1. The actinomycetes and their accession number of **Figure 1**.

Actinomycetes	NCBI GenBank accession number
Streptomyces cavourensis YY01-17	GU575124
Streptomyces cyaneofuscatus M-169.	LN824211
Streptomyces sp. HZP-2216E	KT946782
Streptomyces fulvissimus ZZ406	MF563478
Streptomyces sp. SM18	KT875346
Streptomyces sp. SM10	KT875339
Streptomyces sp. B188M101	KT875335
Streptomyces sp. SBT345	KP238414
Streptomyces anulatus S71	JX007988
Streptomyces cyaneofuscatus M-27	HG965212
Streptomyces sp. HB202	GQ863918
Streptomyces sp. RFCAC02	MK878387
Streptomyces sp. SM11	EU768843
Streptomyces sp. HNM0574	MN446721
Streptomyces sp. SBT348	KP238417
Streptomyces sp. HNM0575	MN446722
Streptomyces sp. SpD081030SC-03	AB539002
Streptomyces sp. XESTO7-21	LC333385
Streptomyces seoulensis IFB-A01	GU722514
Kytococcus sp. HS24	KC478716
Streptomyces sp. 11	EU360175
Streptomyces sp. SM1	DQ887328
Nesterenkonia sp. E5.1	KY296991
Streptomyces coelicoflavus HQA809	KT758401
Micromonospora sp. M42	EF513641
Streptomyces althioticus MSM3	LT627193
Micromonospora sp. WMMB235	JQ716973
Streptomyces sp. strain SCSIO41399	MG912556
Micromonospora sp. CNY-010	KM485658
Streptomyces sp. S6-1	JQ684874
Micromonospora sp. 28ISP2-46	NZ CP059322
Streptomyces sp. HB-N217	MT393585
Micromonospora sp. WMMA1996	KY015148
Streptomyces sp. PKU-MA01144	MN922949
Micromonospora sp. WMMC415	NZ CP046104
Streptomyces sp. HNM0039	MH095989
Micromonospora sp. A5-1	JN627191
Streptomyces sp. G278	MF960781
Micromonospora sp. LHW51205	MG200138
Streptomyces sp. ICN441	MF375083
Verrucosispora sp. FIM060022	KJ143624
Streptomyces variabilis HNS054	JN128288

Verrucosispora sp. SN26 14.1	MK332504
Streptomyces spongiicola HNM0071	KT327868
Verrucosispora sp. FIM06025	EU696750
Verrucosispora sp. FIM06-0036	EU696746
Streptomyces sp. PVA 94-07	JX503966
Streptomyces sp. SM9	KT875340
Salinospora sp. M403	AY562199
Streptomyces sp. SpD081030ME-02	AB539864
Streptomyces sp. GBA 94-10	JX503936
Salinispora arenicola SCSIO SH04	JQ692175
Streptomyces sp. SM17	CP029338
Verrucosispora sp. WMMA107	JF520832
Streptomyces axinellae Pol001	EU683612
Nocardiopsis sp. HB383	GQ863933
Streptomyces sp. DC4-5	LC476780
Nocardiopsis dassonvillei subsp. HQA404	KX809605
Streptomyces sp. GKU220	KY923054
Actinomadura sp. SBMs009	HQ883989
Streptomyces marinus Sp080513GE-26	NR_112841
Actinomadura sp. SpB081030SC-15	AB550669
Streptomyces sp. HDN-10-293	KU980964
Actinomadura sp. WMMB 499	JX101467
Streptomyces sp. CMN-62	KJ794637
Saccharothrix espanaensis An 113	AB248288
Streptomyces sp. PT12	KJ561231
Actinokineospora spheciospongiae EG49	GU318361
Streptomyces sp. SBT349	KP238418
Actinoalloteichus hymeniacidonis HPA177	DQ144222
Streptomyces sp. MCCB 267	KR057432
Aeromicrobium halocynthiae KME 001	FJ042789
Streptomyces sp. M7 15	KR261611
Saccharopolyspora cebuensis SPE 10-1	EF030715
Streptomyces sp. ASO4wet	MT036271
Polaromonas sp. Pch-P	NZ CP046023
Streptomyces sp. SM12	JN037694
Streptomyces carnosus M-207	LN824218
Streptomyces sp. 1053U.I.1a.3b	KF137580
Streptomyces sp. G246	MG917689
Streptomyces sp. HDa1	MG745333
Streptomyces sp. AZS17	FJ999670
Streptomyces sp. RV15	GU318364
Streptomyces sp. Sp080513GE-23	AB498636

2.2 Data analysis of **Figure 21**.

The data of **Table S2** (actinomycetes with 8 genera correspond to 4 structure classes) provides the structural distribution of metabolites from actinomycetes divided by genera for **Figure 3**, which was drawn with analytical and graphic software OriginPro 2018C by the function of 3D Bars.

Table S2. The data of **Figure 21.**

Structure classes / Genera	Alkaloids	Polyketides	Peptides	Other classes
Streptomyces	144	130	60	53
Micromonospora	18	8	4	3
Nocardiopsis	4	9	1	2
Actinomadura	2	5	0	5
Saccharopolyspora	5	6	0	0
Salinispora	6	0	0	0
Micrococcus	0	1	1	5
Other genera	32	28	19	19

2.3 Data analysis of **Figure 22.**

The data of **Table S3** (actinomycetes associated with 12 hosts correspond to 4 structure classes of 536 SMs) provides the structural distribution of metabolites from actinomycetes associated with various hosts for **Figure 22**, which was performed by R version 4.0.5 (R Foundation for Statistical Computing) using the function of geom_point() and geom_jitter() of package *ggplot2*.

Table S3. The data of **Figure 22.**

Structure classes / Hosts	Alkaloids	Polyketides	Peptides	Other classes
Sponge	116	68	54	29
Ascidian	21	29	9	6
Coral	15	14	1	19
Sea cucumber	7	8	0	1
Sea anemone	1	7	2	1
Other invertebrates	33	27	14	13
Marine vertebrates	2	4	3	0
Brown algae	7	8	2	11
Cyanobacteria	0	3	0	0
Green algae	8	8	0	0
Red algae	0	10	0	3
Seagrass	1	1	0	4

2.4 Data analysis of **Figure 23.**

There are three types of nodes in **Table S4** -- Host, Genera, and Classes (4 structure classes of SMs from actinomycetes with 9 genera related to 9 hosts), with the Number of SMs as the flow. These data provide the distribution of secondary metabolites produced by actinomycetes with various genera derived from different hosts for **Figure 23** performed by using R version 4.0.5 using the function of gather_set_data() and geom_parallel_sets() of packages *ggforce* and *ggplot2*, respectively.

Table S4. The data of **Figure 23**.

Host	Genera	Classes	Number
Sponge	*Streptomyces*	Alkaloid	69
Sponge	*Streptomyces*	Polyketide	38
Sponge	*Streptomyces*	Peptide	41
Sponge	*Streptomyces*	Other classes	14
Sponge	*Micromonospora*	Alkaloid	15
Sponge	*Micromonospora*	Polyketide	1
Sponge	*Micromonospora*	Peptide	0
Sponge	*Micromonospora*	Other classes	0
Sponge	*Saccharopolyspora*	Alkaloid	4
Sponge	*Saccharopolyspora*	Polyketide	2
Sponge	*Saccharopolyspora*	Peptide	0
Sponge	*Saccharopolyspora*	Other classes	0
Sponge	*Nocardiopsis*	Alkaloid	0
Sponge	*Nocardiopsis*	Polyketide	9
Sponge	*Nocardiopsis*	Peptide	1
Sponge	*Nocardiopsis*	Other classes	0
Sponge	*Verrucosispora*	Alkaloid	4
Sponge	*Verrucosispora*	Polyketide	0
Sponge	*Verrucosispora*	Peptide	6
Sponge	*Verrucosispora*	Other classes	0
Sponge	*Actinokineospora*	Alkaloid	2
Sponge	*Actinokineospora*	Polyketide	13
Sponge	*Actinokineospora*	Peptide	1
Sponge	*Actinokineospora*	Other classes	0
Sponge	*Salinispora*	Alkaloid	3
Sponge	*Salinispora*	Polyketide	0
Sponge	*Salinispora*	Peptide	0
Sponge	*Salinispora*	Other classes	0
Sponge	*Actinomadura*	Alkaloid	0
Sponge	*Actinomadura*	Polyketide	0
Sponge	*Actinomadura*	Peptide	0
Sponge	*Actinomadura*	Other classes	4
Sponge	Other genera	Alkaloid	25

Sponge	Other genera	Polyketide	5
Sponge	Other genera	Peptide	4
Sponge	Other genera	Other classes	11
Ascidian	*Streptomyces*	Alkaloid	12
Ascidian	*Streptomyces*	Polyketide	19
Ascidian	*Streptomyces*	Peptide	4
Ascidian	*Streptomyces*	Other classes	0
Ascidian	*Micromonospora*	Alkaloid	1
Ascidian	*Micromonospora*	Polyketide	4
Ascidian	*Micromonospora*	Peptide	0
Ascidian	*Micromonospora*	Other classes	2
Ascidian	*Actinomadura*	Alkaloid	2
Ascidian	*Actinomadura*	Polyketide	5
Ascidian	*Actinomadura*	Peptide	0
Ascidian	*Actinomadura*	Other classes	1
Ascidian	*Salinispora*	Alkaloid	3
Ascidian	*Salinispora*	Polyketide	0
Ascidian	*Salinispora*	Peptide	0
Ascidian	*Salinispora*	Other classes	0
Ascidian	*Nocardiopsis*	Alkaloid	2
Ascidian	*Nocardiopsis*	Polyketide	0
Ascidian	*Nocardiopsis*	Peptide	0
Ascidian	*Nocardiopsis*	Other classes	0
Ascidian	Other genera	Alkaloid	1
Ascidian	Other genera	Polyketide	1
Ascidian	Other genera	Peptide	5
Ascidian	Other genera	Other classes	3
Coral	*Streptomyces*	Alkaloid	12
Coral	*Streptomyces*	Polyketide	13
Coral	*Streptomyces*	Peptide	0
Coral	*Streptomyces*	Other classes	16
Coral	*Micromonospora*	Alkaloid	0
Coral	*Micromonospora*	Polyketide	1
Coral	*Micromonospora*	Peptide	1
Coral	*Micromonospora*	Other classes	0
Coral	Other genera	Alkaloid	3
Coral	Other genera	Polyketide	0
Coral	Other genera	Peptide	0
Coral	Other genera	Other classes	3
Other invertebrates	*Streptomyces*	Alkaloid	37
Other invertebrates	*Streptomyces*	Polyketide	28
Other invertebrates	*Streptomyces*	Peptide	10
Other invertebrates	*Streptomyces*	Other classes	9
Other invertebrates	*Micromonospora*	Alkaloid	2

Other invertebrates	*Micromonospora*	Polyketide	0
Other invertebrates	*Micromonospora*	Peptide	3
Other invertebrates	*Micromonospora*	Other classes	1
Other invertebrates	*Saccharopolyspora*	Alkaloid	1
Other invertebrates	*Saccharopolyspora*	Polyketide	4
Other invertebrates	*Saccharopolyspora*	Peptide	0
Other invertebrates	*Saccharopolyspora*	Other classes	0
Other invertebrates	Other genera	Alkaloid	1
Other invertebrates	Other genera	Polyketide	10
Other invertebrates	Other genera	Peptide	3
Other invertebrates	Other genera	Other classes	6
Marine vertebrates	*Streptomyces*	Alkaloid	0
Marine vertebrates	*Streptomyces*	Polyketide	4
Marine vertebrates	*Streptomyces*	Peptide	3
Marine vertebrates	*Streptomyces*	Other classes	0
Marine vertebrates	*Nocardiopsis*	Alkaloid	1
Marine vertebrates	*Nocardiopsis*	Polyketide	0
Marine vertebrates	*Nocardiopsis*	Peptide	0
Marine vertebrates	*Nocardiopsis*	Other classes	0
Marine vertebrates	*Micromonospora*	Alkaloid	0
Marine vertebrates	*Micromonospora*	Polyketide	1
Marine vertebrates	*Micromonospora*	Peptide	0
Marine vertebrates	*Micromonospora*	Other classes	0
Brown algae	*Streptomyces*	Alkaloid	4
Brown algae	*Streptomyces*	Polyketide	7
Brown algae	*Streptomyces*	Peptide	2
Brown algae	*Streptomyces*	Other classes	8
Brown algae	*Nocardiopsis*	Alkaloid	1
Brown algae	*Nocardiopsis*	Polyketide	0
Brown algae	*Nocardiopsis*	Peptide	0
Brown algae	*Nocardiopsis*	Other classes	2
Brown algae	*Micromonospora*	Alkaloid	0
Brown algae	*Micromonospora*	Polyketide	1
Brown algae	*Micromonospora*	Peptide	0
Brown algae	*Micromonospora*	Other classes	0
Brown algae	Other genera	Alkaloid	2
Brown algae	Other genera	Polyketide	0
Brown algae	Other genera	Peptide	0
Brown algae	Other genera	Other classes	1
Green algae	*Streptomyces*	Alkaloid	8
Green algae	*Streptomyces*	Polyketide	8
Green algae	*Streptomyces*	Peptide	0
Green algae	*Streptomyces*	Other classes	0
Red algae	*Streptomyces*	Alkaloid	0

Red algae	*Streptomyces*	Polyketide	10
Red algae	*Streptomyces*	Peptide	0
Red algae	*Streptomyces*	Other classes	3
Other plants	*Streptomyces*	Alkaloid	1
Other plants	*Streptomyces*	Polyketide	4
Other plants	*Streptomyces*	Peptide	0
Other plants	*Streptomyces*	Other classes	4

2.5 Data analysis of **Figure 24.**

The data of **Table S5** provides the structural distribution of metabolites from three dominant genera in the main hosts -- sponge and ascidian for **Figure 24**, which was drawn with the Column function of OriginPro 2018C.

Table S5. The data of **Figure 24**.

Host-Genera-Classes	Number of natural products
Sponge - *Streptomyces* - alkaloid	69
Sponge - *Streptomyces* - polyketide	38
Ascidian - *Streptomyces* - alkaloid	12
Ascidian - *Streptomyces* - polyketide	19
Sponge - *Micromonospora* - alkaloid	15
Sponge - *Micromonospora* - polyketide	1
Ascidian - *Micromonospora* - alkaloid	1
Ascidian - *Micromonospora* - polyketide	4
Sponge - *Saccharopolyspora* - alkaloid	4
Sponge - *Saccharopolyspora* - polyketide	2
Ascidian - *Actinomadura* - alkaloid	2
Ascidian - *Actinomadura* - polyketide	5

2.6 Data analysis of **Figure 25.**

The data of **Table S6** (11 bioactivities correspond to 4 structure classes) provides the diverse distribution of biological activity with different structures for **Figure 25** drawn by the 3D Bars function of OriginPro 2018C software.

Table S6. The data of **Figure 25**.

Structure classes / Bioactivities	Alkaloids	Polyketides	Peptides	Other classes
antibacterial	87	69	29	20
anticancer	69	54	28	6
enzyme inhibitory	23	6	9	3
antiparasite	10	12	3	2
antioxidant	3	6	2	2
antichlamydia	5	0	0	0
antiviral	3	0	0	0

antiinflammatory	4	0	2	3
antifouling	1	0	3	4
antiradiation	0	2	0	0
other activies	4	18	0	9

3. Table S7. The summary of all secondary metabolites including information on separation sources, structural types, and biological activities.

Host	*Actinomycetes*		Metabolites	Classes	Activities	Ref
Sponge	*Micromonospora sp.*	L-31-CLCO-002	4′-N-methyl-5′-hydroxystaurosporine (**1**)	alkaloid	cytotoxic activities	9, 10
			5′ -hydroxystaurosporine (**2**)	alkaloid	cytotoxic activities	
			Staurosporine (**3**)	alkaloid	cytotoxic activities	
	Saccharopolyspora sp.		Metacycloprodigiosin (**4**)	alkaloid	cytotoxic activities	13
			Undecylprodigiosin (**5**)	alkaloid	cytotoxic activities	
	Micromonospora sp.	M42	Manzamine A (**6**)	alkaloid	antibacterial, antiviral activity	9
			8-hydroxy manzamine (**7**)	alkaloid	antibacterial, antiviral activity	
	Salinispora sp.	M403	Rifamycin B (**8**)	alkaloid	antibacterial activity	9, 14
			Rifamycin SV (**9**)	alkaloid	antibacterial activity	
	Streptomyces sp.	Ni-80	Urauchimycin A (**10**)	alkaloid	antifungal activity	9, 17
			Urauchimycin B (**11**)	alkaloid	antifungal activity	
	Streptomyces sp.	HB202	Streptophenazines A,C-H (**12**, **14-19**)	alkaloid	antibacterial activity	18, 19
			Streptophenazines B(**13**)	alkaloid	antibacterial activity and cytotoxicity	
	Saccharopolyspora cebuensis	SPE 10-1	Cebulactams A1 and A2 (**S1**, **S2**)	alkaloid	unknown	9, 20
	Streptomyces sp.	11	Staurosporine (**3**)	alkaloid	anti-parasitic activity and cytotoxicity	9, 21
	Streptomyces sp.	Sp080513GE-26	5-iminoaranciamycin (**S3**)	alkaloid	unknown	9, 22
			Tetracenoquinocin (**127**)	polyketide	cytotoxicity	
			Aranciamycin (**128**)	polyketide	cytotoxicity	
			SM 173B (**129**)	polyketide	antibiotic	
	Streptomyces sp.	SpC080624SC-11	JBIR 46–48 (**20-22**)	alkaloid	cytotoxic activity	9, 23, 24
	Brevibacterium sp.	KMD 003	6-hydroxymethyl-1-phenazine-carboxamide (**23**); 1,6-phenazinedimethanol (**24**)	alkaloid	antibacterial activities	9

Streptomyces sp.	SpD081030ME-02	JBIR-58 (**25**)	alkaloid	cytotoxic activity	9, 25
Streptomyces sp.	DA22	Streptomycindole (**S4**)	alkaloid	unknown	26
		N-phenylacetyl-L-tryptophan (**S5**)	alkaloid	unknown	
Streptomyces carnosus	AZS17	Lobophorins C and D (**26**, **27**)	alkaloid	cytotoxic activity	27
Micromonospora sp.	RV115	Diazepinomicin (**28**)	alkaloid	cytotoxic activity, antiparasitic activity, antioxidant activity and enzyme inhibitory activity	9, 28
Streptomyces sp.	CMS JV M18_3	WS-9659 A (**29**)	alkaloid	enzyme inhibitory activity	3, 29
		Chloro- Dihydroquinone 1-4 (**136-139**)	polyketide	antibacterial activity and cytotoxic activity	
		Naphthomevalin 1 (SF2415B1) (**140**)	polyketide	antimicrobial activities	
		SF2415B3 (**141**)	polyketide	anti-biofilm activity	
Nocardiopsis sp.	KMF-002	Nocatriones A and B (**142**, **143**)	polyketide	anti-radiation activity	99
Streptomyces tateyamensis	NBRC 105047	JBIR-107 (**S8**)	alkaloid	unknown	31
Streptomyces sp.	RM72	JBIR 109 -111 (**30-32**)	alkaloid	enzyme inhibitory activity	9, 30
		Trichostatin A (**S6**), Trichostatic acid (**S7**)	alkaloid	unknown	
Streptomyces anulatus	S71	2-hydroxy-1-(1H-indol-3-yl)ethan-1-one (**S9**); 3-hydroxy-4-(2-(2-hydroxy-3,5-dimethylphenyl)-2-oxoethyl)piperidine-2,6-dione (S**10**); 4-(2-(2-hydroxy-3-(hydroxymethyl)-5-methylphenyl)-2-oxoethyl)piperidine-2,6-dione (**S11**)	alkaloid	unknown	32
		4-(2-(2-hydroxy-5-(hydroxymethyl)-3-methylphenyl)-2-oxoethyl)piperidine-2,6-dione (**33**)	alkaloid	antibacterial activity	

Salinispora sp	FS-0034	Rifamycin W (**34**)	alkaloid	antibacterial activity	33
Streptomyces sp.	M7_15	Frigocyclinone (**35**)	alkaloid	cytotoxic activity	34
		Monacyclinones A–F (**36-41**)	alkaloid	cytotoxic activity and antibacterial activity	
		Dimethyldehydrorabelomycin (**S72**)	polyketide	unknown	
Streptomyces sp.	RV15	SF2446 A2 (**42**)	alkaloid	antibacterial, anti-mycoplasma, anti-chlamydia, and anti-parasite activities	35
		Cyclodysidins A–D (**S110-S113**)	peptides	unknown	
Streptomyces sp.	LS298	Tirandamycin A (**43**)	alkaloid	enzyme inhibitory activity and antibacterial activity	36
		Tirandamycin B (**44**)	alkaloid	enzyme inhibitory activity and antiparasite activity	
		Staurosporine (**3**)	alkaloid	antifungal activity, protein kinase C inhibitory activity, and cytotoxic activity	
		Quinomycin G (**242**)	peptides	antibacterial activities and anti-tumor activities	
		Cyclo-(L-Pro-4-OH-L-Leu) (**S117**)	peptides	unknown	
Streptomyces sp.	SBT345	Strepoxazine A (**45**)	alkaloid	cytotoxic activity	40, 41
		Ageloline A (**46**)	alkaloid	antioxidant activity and anti-chlamydia activity	
		Phencomycin (**47**), Tubermycin B (**48**)	alkaloid	antibacterial activity	
Streptomyces albus	PVA94-07	Deferoxamine analogues (**S12-S14**)	alkaloid	unknown	42
		Deferoxamine analogue (**49**)	alkaloid	antibacterial activity	

		Rhodozepinone (**50**)	alkaloid	antibacterial, antitrypanosomal and antiparasite activities	43
Rhodococcus sp.	UA13	2-amino-3-[2(1H)-quinolinon-4-yl]propionic acid (**S15**), indole-3-acetic acid (**S16**)	alkaloid	unknown	
		3-hydroxy-2-methyl-4H-pyran-4-one (maltol) (**S74**)	polyketide	unknown	
		Henyl acetic acid methyl ester (**S159**), Tryptophan (**S160**)	Others	unknown	
Rubrobacter radiotolerans		Dimeric indole derivatives (**51-53**)	alkaloid	antichlamydia activity	44
		2,3-dihydroxybenzamide (**54**)	alkaloid	cytotoxicity	45
Streptomyces sp.	SBT348	3-hydroxy-2-methyl-4H-pyran-4-one (maltol) (**S74**)	polyketide	unknown	
		Petrocidin A (**243**)	peptides	cytotoxicity	
Streptomyces sp.	CMN-62	Anthranosides A and B (**S17**, **S18**)	alkaloid	unknown	46
		Anthranoside C (**55**)	alkaloid	anti-influenza H1N1 activity	
		Saccharomonosporine A (**56**); (S)-6-bromo-3-hydroxy-3-(1H-indol-3-yl)indolin-2-one (**57**)	alkaloid	enzyme inhibitory activity and cytotoxic activity	47
Saccharomonospora sp.	UR22	convolutamydine F (**S19**); 3,3'-(ethane-1,1-diyl)bis(1H-indole) (**S20**); 1H-indole-3-carbaldehyde (**S21**); (Z)-2-hydroxy-3-(1H-indol-3-yl)acrylic acid (**S22**); 3-methyl-1H-indole (**S23**)	alkaloid	unknown	
		2-(1H-indol-3-yl)ethan-1-ol (**58**)	alkaloid	antimicrobial activities	
		Compound **S75**	polyketide	unknown	
		1-hydroxy-2-naphthoic acid (**S161**);	others	unknown	

		1,4-dihydroxy-2-naphthoic acid (**S162**)			
Micromonospora carbonacea	LS276	Tetrocarcin Q (**59**), AC6H (**61**), Tetrocarcin N (**62**), Tetrocarcin H (**63**)	alkaloid	antibacterial activity	48
		Tetrocarcin A (**60**), Arisostatin A (**64**)	alkaloid	antibacterial activity and antitumor activity	
		Tetrocarcin F1 (**S24**)	alkaloid	unknown	
Streptomyces tirandamycinicus sp. nov.	HNM0039T	Tirandamycins A and B (**43**, **44**)	alkaloid	antibacterial activity	49
Verrucosispora sp.	FIM06-0036	2-ethylhexyl-1H-imidazole-4-carboxylate (**65**)	alkaloid	antimicrobial activities	50
		1H-imidazole-4-carboxylate (**S25**)	alkaloid	unknown	
Verrucosispora sp.	FIM06025	(2-(hydroxymethyl)-3-methylaziridin-1-yl)(2-hydroxyphenyl)methanone (**66**)	alkaloid	antimicrobial activity	51
		2-(1-hydroxyethyl)-3,4-dihydrobenzo[f][1,4]oxazepin-5(2H)-one (**S26**)	alkaloid	unknown	
Actinokineospora spheciospongiae sp. nov.		Fridamycins I (**S27**)	alkaloid	unknown	52
		Fridamycin H (**154**)	polyketide	antiparasite activity	
		Actinosporins C, D (**144**, **145**)	polyketide	antioxidant activity	
		Actinosporin G (**155**)	polyketide	unknown	
Streptomyces. zhaozhouensis subsp. mycale. subsp. nov.	MCCB267	Ikarugamycin (**67**), Clifednamide A (**68**), 30-oxo-28- N-methylikarugamycin (**69**), 28-N-methylikarugamycin (**70**)	alkaloid	cytotoxic activity	53
Streptomyces rochei	MB037	Borrelindine J and K (**71**, **72**)	alkaloid	antibacterial activity	54
		Borrelidin (**S28**), Borrelidin F (**S29**)	alkaloid	unknown	

		7-methoxy-2,3-dimethylchromone-4-one (**S76**)	polyketide	unknown	
Streptomyces sp.	G248	9H-pyrido[3,4-b]indole (**S30**), 9H-purin-6-amine (**S31**)	alkaloid	unknown	55
		(2S,2''S)-6-lavandulyl-7,4'-dimethoxy-5,2'-dihydroxylflavanone (**158**); (2S,2''S)-6-lavandulyl-5,7,2',4'-tetrahydroxylflavanone (**159**); (2''S)-5′-lavandulyl-2′-methoxy-2,4,4′,6′-tetrahydroxylchalcone (**160**)	polyketide	antimicrobial activity	
		(2S,2''S)-6-lavandulyl-7-methoxy-5,2',4'-trihydroxylflavanone (**161**); 6-prenyl-4'-methoxy-5,7-dihydroxylflavanone (**162**)	polyketide	antibacterial activity	
		Cyclo (L-Pro-L-Leu) (**246**)	peptide	cytotoxic activity	
		Cyclo(L-Pro-L-Tyr) (**S120**); Cyclo(L-Pro-L-Phe) (**S123**)	peptide	unknown	
Streptomyces sp.	G246	9H-pyrido[3,4-b]indole (**S30**), indole-3-acetic acid (**S16**)	alkaloid	unknown	56
		(S)-2-(2,4-dihydroxyphenyl)-5-hydroxy-7-methoxy-6-((S)-5-methyl-2-(prop-1-en-2-yl)hex-4-en-1-yl)chroman-4-one (**156**), (S,E)-1-(2,6-dihydroxy-4-methoxy-3-(5-methyl-2-(prop-1-en-2-yl)hex-4-en-1-yl)phenyl)-3-(2,4-dihydroxyphenyl)prop-2-en-1-one (**157**)	polyketide	antimicrobial activity	
		(3S,8aS)-3-methylhexahydropyrrolo[1,2-a]pyrazine-1,4-dione (**S119**), Cyclo(L-Pro-L-Tyr)	peptide	unknown	

		(**S120**), (3S,8aS)-7-hydroxy-3-(4-hydroxybenzyl)hexahydropyrrolo[1,2-a]pyrazine-1,4-dione (**S121**), (3S,8aS)-3-(2-(methylthio)ethyl)hexahydropyrrolo[1,2-a]pyrazine-1,4-dione (**S122**)			
		L-tryptophan (**S160**), L-phenylalanine (**S163**)	others	unknown	
Micromonospora ferruginea sp.	28ISP2-46T	Quinocycline B (kosinostatin) (**73**)	alkaloid	antibiotic activity, enzyme inhibitory activity	57
		Isoquinocycline B (**74**)	alkaloid	antibiotic activity, enzyme inhibitory activity and cytotoxicity	
Micromonospora sp.	L-25-ES25-008	IB-96212 (**124**)	polyketide	cytotoxic activity	9, 90
Saccharopolyspora taberi	PEM-06-F23-019B	PM070747 (**125**), PD116740 (**126**)	polyketide	antitumor activity	91
Nocardiopsis	HB383	Nocapyrones A–D (**S65-S68**)	polyketide	unknown	9, 92
		(2E/5Z)-2-[(4-methoxyphenyl)methylene]-5-(2-methylpropylidene)-3,6-piperazinedione (**231**)	peptide	cytotoxic activity	
Streptomyces axinellae	Pol001T	Tetromycin 1 (**S69**)	alkaloid	unknown	9, 93
		Tetromycin 2 (**S70**), Tetromycin B (**S71**)	polyketide	unknown	
		Tetromycins 3 and 4 (**130**, **131**)	polyketide	enzyme inhibitory activity and antibacterial activity	
Streptomycetes sp.	BCC45596	Urdamycinone E (**132**), Urdamycinone G (**133**), Dehydroxyaquayamycin (**134**), Urdamycin E (**135**)	polyketide	antiparasite activity, antibacterial activity	94, 95
Actinokineospora sp.	EG49	Actinosporins A,B (**146**, **147**)	polyketide	anti-trypanosomal	59, 100, 101
		Actinosporins C,D (**144**, **145**)	polyketide	antioxidant activity	
		UK-2B (**75**)	alkaloid	antifungal activity	
		Actinosporins E (**163**), G (**155**), and H (**164**)	polyketide	antimalarial activity	
		Actinosporins F (**S77**)	polyketide	unknown	

		Tetrangulol (**165**)	polyketide	antimalarial activity	
Micrococcus sp.	EG45	Microluside A (**148**)	polyketide	antibacterial activity	102
Streptomyces sp.	HDN-10-293	Naquihexcin A (**149**), (−)-BE-52440A (**150**)	polyketide	cytotoxicity	103
		Naquihexcin B (**S73**)	polyketide	unknown	
Nocardiopsis sp.	HB-J378	Nocardiopsistins A-C (**151-153**)	polyketide	antibacterial activity	104
Streptomyces sp.	DA18	(3R,8aS)-3-benzylhexahydropyrrolo[1,2-a]pyrazine-1,4-dione (**227**)	peptide	antimicrobial activity	128
		(3R,8aR)-3-benzylhexahydropyrrolo[1,2-a]pyrazine-1,4-dione (**228**)	peptide	antimicrobial activity, antifouling activity	
		Cyclo (6-OH-D-Pro-L-Phe) (**229**)	peptide	cytotoxic activity	
		(3R,8aS)-3-isopropylhexahydropyrrolo[1,2-a]pyrazine-1,4-dione (**S105**)	peptide	unknown	
Streptomyces sp.	22	Valinomycin (**230**)	peptide	antiparasite activity and cytotoxic activity	9, 21
Streptomyces sp.	34	Valinomycin (**230**)	peptide	Antiparasite activity and cytotoxic activity	9, 21
Streptomyces sp.	NBRC 105896	JBIR-31 (**232**)	peptide	cytotoxic activity	9, 129
Streptomyces sp.	Sp080513GE-23	JBIR-34 (**233**) and JBIR-35 (**234**)	peptide	DPPH radical scavenging activity	130
Streptomyces sp.	SpD081030SC-03	JBIR-56 (**S106**) and JBIR-57 (**S107**)	peptide	unknown	9, 131
Verrucosispora sp.	WMMA107	Thiocoraline (**235**), 22′-Deoxythiocoraline (**236**), Thiochondrilline C (**237**), 12′-sulfoxythiocoraline (**238**)	peptide	cytotoxic activity	9, 132
		Thiochondrilline A (**S108**)and B (**S109**)	peptide	unknown	
Streptomyces	M1087	Nocardamine (**S114**)	peptide	unknown	134
		1,12-Dihydroxy-1,6,12,17,23,28-hexaazacyclotritriacontane-2,5,13,16,24,27-hexone (**239**), 1,11,22-Trihydroxy-1,6,11,16,22,27-hexaazacyclodotriacontane-2,5,12,15,23,26-hexone	peptide	enzyme inhibitory activity	

		(**240**)			
Kocuria palustris.	F-276,345	Kocurin (**241**)	peptide	antibacterial activity	136, 137
Streptomyces sp.	GKU 220	Rakicidin F (**244**)<br>Rakicidin C (**S118**)	peptide<br>peptide	antibacterial activity<br>unknown	138
Actinokineospora spheciospongiae	DSM45935	Actinokineosin (**245**)	peptide	antibacterial activity	139
Streptomyces sp.	LHW52447	Actinomycins D1-D4, D (**247-251**)	peptide	antibacterial activity and cytotoxic activity	140
Streptomyces sp	Call-36	Actinozine A (**252**), Cyclo(2-OH-D-Pro-L-Leu) (**253**)<br>Cyclo(D-Pro-L-Phe) (**254**)<br>Cyclo(L-Pro-L-Phe) (**S123**)<br>Thymidine-3-mercaptocarbamic acid (**S165**) and thymidine-3-thioamine (**S166**)	peptide<br>peptide<br>peptide<br>nucleoside	antibacterial activity<br>cytotoxicity<br>unknown<br>unknown	143
Actinomadura sp.	SpB081030SC-15	JBIR-65 (**275**)	terpenoid	protective activity of neuronal hybridoma N18-RE-105 cells from L-glutamate toxicity	9, 154
Actinomadura sp.	SBMs009	Bendigoles E,F (**282**, **283**)<br>Bendigole D (**281**)	steroid<br>steroid	anti-inammatory activity, anti-glucocorticoid receptor translocation activity<br>anti-inammatory activity, anti-glucocorticoid receptor translocation activity and cytotoxicity	9, 159
Micrococcus luteus	R-1588-10	Lutoside (**S137**)<br>2,4,4′-trichloro-2′-hydroxydiphenylether (**286**)	others<br>others	unknown<br>antimicrobial activity	9, 160
Streptomyces sp.	T03	Butenolide (**294**)	others	antiparasite activity	9, 21
Streptomyces microflavus	HVG29	3-acetyl-1-((2R,4S,5R)-4-hydroxy-5-(hydroxymethyl)tetrahydrofuran-2-yl)-5-methylpyr	others	unknown	161

		imidine-2,4(1H,3H)-dione (**S143**); 1-((2R,4S,5R)-4-hydroxy-5-(hydroxymethyl) tetrahydrofuran-2-yl)-3,5-dimethylpyrimidine-2,4(1H,3H)-dione (**S144**); 1-((2R,4S,5R)-4-hydroxy-5-(hydroxymethyl) tetrahydrofuran-2-yl)-3-methylpyrimidine-2,4(1H,3H)-dione (**S145**)			
Streptomyces sp	NIO 10068	Proline–glycine (**S115**); N-amido-α- proline (**S116**)	dipeptide	unknown	135
		Cinnamic acid (**296**)	aromatic acid	QS antagonist activity, bactericidal activity	
Microbacterium sp.	HP2	1,2-*O*-diacyl-3-[β-glucopyranosyl-(1–6)-β-glucopyranosyl)]-glycerol (**S147**); 1-O-acyl-3-[6-O-acetyl-α-glucopyranosyl-(1–3)-(6-O-acyl-α-mannopyranosyl)]glycerol (**S148**); 1,2-O-diacyl-3-[β-galactofuranosyl)]glycerol (**S149**); Diphosphatidylglycerol (**S150**)	others	unknown	9, 163
		1-O-acyl-3-[α-glucopyranosyl-(1–3)-(6-O-acyl-α-mannopyranosyl)]glycerol,diphosphatidylglycerol (**299**)	others	antitumor activity	
Streptomyces sp.	RM66	Phencomycin (**47**)	alkaloid	antibacterial activities	58
		Tubermycin B (**48**)	alkaloid	antimicrobial activity	
		Phenazine (**S32**), Pyocyanine (**S33**), Mycomethoxin B (**S34**), Phencomycin methyl ester (**S35**), 2-methoxy-1-phenazinecarboxylic acid (**S36**), 1-hydroxymethyl-6-carboxyphenazine (**S37**)	alkaloid	unknown	

			Manadoperoxide H (**285**)	Steroid	anti-trypanosomal activity	
			Sulfate F (**S136**)	Steroid	unknown	
			Ethyl plakortide Z (**305**)	others	antitumour and cytotoxicity	
			Seco-plakortide Z (**S167**), Actinopolysporin B (**S168**), and Acanthosterol G (**S169**)	others	unknown	
	Nesterenkonia sp.	MSA31	Nesfactin (**255**)	peptide	antibacterial activity	144
	Rhodococcus sp.	UR59	Kaimonolide B (**166**)	polyketide	plant growth inhibitor	59
			8,15-Dideoxylankanolide (**S78**)	polyketide	unknown	
			Rhodopeptins C1, C2, and B5 (**256-258**)	peptide	antifungal activity	
			Mitomycin K (**76**)	alkaloid	antitumor activity	
			Piericidin F (**77**), Migrastatin (**78**)	alkaloid	anticancer activity	
Coral	*Streptomyces sp.*	OUCMDZ-1703	Watasemycin A (**79**), Aerugine (**80**)	alkaloid	antibacterial activity	60
			Pulicatin G (**S38**), Pyrrole-2-carboxamide (**S39**), Furan-2-carboxamide (**S40**), 1-(3,5-dihydroxyphenyl)ethanone (**S41**)	alkaloid	unknown	
			Strepchloritides A (**168**) and B (**169**)	polyketide	cytotoxicity	
	Streptomyces sp.	M-207	Lobophorin K (**81**)	alkaloid	cytotoxic activity and antibacterial activity	61
	Streptomyces sp.	SCSIO 41399	Isotirandamycin B (**82**), Tirandamycins A (**43**) and B (**44**)	alkaloid	antibacterial activity	62
			Anthracycline derivatives (**83-84**)	alkaloid	cytotoxic activities	
			Aranciamycin K (**S81**), Anthracycline derivative (**S82**)	polyketide	unknown	
			Anthracycline derivative (**170**)	polyketide	cytotoxic activities	
	Pseudonocardia sp.	SCSIO 11457	11457A (**S42**), 11457B (**S43**), 1H-indole-2-carbal-dehyde (**S44**)	alkaloid	unknown	63
	Streptomyces sp.	PG-19	Octalactin A (**167**)	polyketide	cytotoxicity	105
			Octalactin B (**S79**)	polyketide	unknown	
	Micromonospora sp.	A5-1	7b, 13-dihydro-7-O-methyl jadomycin B	polyketide	unknown	106

		(**S80**)			
Streptomyces cyaneofuscatus	M-169	Anthracimycin B (**171**), Anthracimycin (**172**)	polyketide	antibacterial activity	107
Streptomyces variabilis		1-hydroxy-1-norresistomycin (HNM) (**173**)	polyketide	antibacterial activities and cytotoxic activity	108
Streptomyces sp.	DC4-5	Iseolides A–C (**174-176**)	polyketide	antifungal activity	109
Micromonospora sp.	L-13-ACM2-092	Thiocoraline (**235**)	peptide	cytotoxic activity, Enzyme inhibitory activity and antibacterial activity	145, 146
Streptomyces sp.	ZJG1	Sesquiterpenes **S131-S133**	terpenoid	unknown	155
		Sesquiterpenes **276**	terpenoid	free radical scavenging and acetylcholinesterase inhibitory activity	
		Sesquiterpenes **277**	terpenoid	redical scavenging activity、hemolytic activity and acetylcholinesterase inhibitory activity	
Micrococcus sp		(6E,8Z)- (**303**) and (6E,8E)-5-oxo-6,8-tetradecadienoic acids (**304**)	fatty acid	antibacterial activity, agonistic activity against PPARs	166
Streptomyces griseorubens sp.	ASMR4	Oxaphenalene derivative (**S151**), Ferulic acid (**S152**), Glycerol linoleate (**S153**), Linoleic acid methyl ester (**S154**), (3R,4R)-3,4-dihydroxy-3-methylpentan-2-one/ (3S,4R)-3,4-dihydroxy-3-methylpentan-2-one (**S155**), Anthranilic acid (**S156**), Phenylacetic acid (**S157**), and benzoic acid (**S158**)	others	unknown	164
Nesterenkonia	E5.1	Nesteretal A (**302**)	others	RXRα transcriptional	165

	halobia (Micrococcus halobius)				activation activity	
	Streptomyces sp.	RKBH-B7	Guanahanolide A (**279**)	terpenoid	cytotoxicity	157
	Streptomyces albogriseolus	SY67903	Microeunicellol A (**280**)	terpenoid	cytotoxicity	158
			Microeunicellol B (**S135**)	terpenoid	unknown	
Ascidian	*Salinispora pacifica*	LL-37I366	Lomaiviticin A (**85**)	alkaloid	DNA-damaging agent, antibacterial activity, cytotoxicity	64-66
			Lomaiviticin B (**86**)	alkaloid	DNA-damaging agent, antibacterial activity	
	Micromonospora sp.	DPJ12	Diazepinomicin (**28**)	alkaloid	antimicrobial activity	67
	Streptomyces sp.	YM14-060	Piericidins C7, C8, A1, A2 (**87-90**)	alkaloid	cytotoxicity	64, 68
	Streptomyces sp.	JP90	Organophosphate (S)- cinnamoyl-phosphoramide (**91**)	alkaloid	enzyme inhibitory activity	64
	Salinispora arenicola	CNR-647	Arenimycin (**92**)	alkaloid	antimicrobial activities and cytotoxicity	64, 69
	Streptomyces sp.		Bohemamine (**S46**)	alkaloid	unknown	64
	Actinomadura sp.	WMMB-499	Forazoline A (**93**)	alkaloid	antifungal activity	71
			Forazoline B (**S47**)	alkaloid	unknown	
	Streptomyces sp.	Did-27	(S)-6-(sec-butyl)-3-isopropylpyrazin-2(1H)-one (**94**), (S)-6-(sec-butyl)-3-isobutylpyrazin-2(1H)-one (**95**); (1H)-pyrazinones analogues deoxymutaaspergillic acid (**96**); 3,6-diisobutyl-2(1H)- pyrazinone (**97**) and 3,6-disec-butyl-2(1H)-pyrazinone (**98**)	alkaloid	cytotoxicity	64, 72
			(S)-3-(sec-butyl)-6-isopropylpyrazin-2(1H)-one (**S48**)	alkaloid	unknown	

		Cyclo (6-OH-D-Pro-L-Phe) (**229**); Bacillusamide B (**261**); Cyclo (L-Pro-L-Leu) (**246**); Cyclo (L-Pro-L-Ile) (**262**)	peptide	cytotoxic activities	
Nocardiopsis dassonvillei	HQA404	1,6-dihydroxyphenazine (**99**)	alkaloid	antimicrobial activity, cytotoxic activity, enzyme inhibiting activity	64
		2-(acetylamino)-phenol (**100**)	alkaloid	cytotoxic activity	
Nocardia sp.	KMM 3749	Ubiquinone Q9 (**177**)	polyketide	cytotoxicity	64, 110
Streptomyces sp.	JP95	Griseorhodin A (**178**)	polyketide	enzyme inhibitory activity	64, 111
Streptomyces sp.	#N1-78-1	Bisanthraquinones 1 (**179**) and 2 (**180**)	polyketide	antimicrobial activities and cytotoxic activity	64, 112
		Derivative 3 (**181**)	polyketide	cytotoxic activity	
Micromonospora sp.		4,6,11-trihydroxy-9-propyltetracene-5,12-dione (**182**) and 10β-carbomethoxy-7,8,9,10-tetrahydro-4,6,7α,9α,11-pentahydroxy-9-propyltetra-cene-5,12-dione (**183**)	polyketide	cytotoxic activity	113
		1-methoxy-9-propyltetra-cene-6,11-dione (**S83**) and 7,8,9,10-tetrahydro-9-hydroxy-1-methoxy-9-propyltetracene-6,11-dione (**S84**)	polyketide	unknown	
Actinomadura sp.	WMMB499	Halomadurones A-B (**S85**, **S86**)	polyketide	unknown	64, 114, 115
		Halomadurones C (**184**) and D (**185**)	polyketide	antioxidant activity	
		Ecteinamycin (**186**)	polyketide	antibacterial activity	
Streptomyces sp.	SCSGAA 0027	Nahuoic acid A (**191**)	polyketide	enzyme inhibitory activity and antibiofilm activity	116, 117
		Nahuoic acids B–E (**187-190**)	polyketide	antibiofilm activity	
		Pteridic acids C (**S87**) and D (**S88**)	polyketide	unknown	
		Pteridic acids E-G (**192-194**)	polyketide	antibacterial activity	
Streptomyces coelicoflavus	HQA809	Germicidin (**195**) and 6-isopropyl group-3-ethyl-4-hydroxy-2-pyrone (**196**)	polyketide	cytotoxic activity	64

	Streptomyces sp.	PTY087I2	Granaticin (**197**), Granatomycin D (**198**), Dihydrogranaticin B (**199**)	polyketide	antibacterial activity	64, 118
	Nocardia sp.		Peptidolipins B (**259**) and E (**260**)	peptide	antimicrobial activities	64, 72
			peptidolipins C,D,F (**S124-S126**)	peptide	unknown	
	Micromonospora sp.	WMMC-218	Micromonohalimane A (**S134**)	terpenoid	unknown	156
			Micromonohalimane B (**278**)	terpenoid	antibacterial activity	
	Aeromicrobium halocynthiae	KME 001	Taurocholic acid (**S45**)	alkaloid	unknown	64, 70
	Actinomadura sp		Ecteinamycin (**295**)	others	antimicrobial activity	64, 115
	Solwaraspora sp.	WMMB329	Solwaric acids A (**297**) and B (**298**)	aromatic acid	antibacterial activity	64, 162
			2,4,6-triphenyl-1-hexene (**S146**)	others	unknown	
Other invertebrates	*Streptomyces sp.*	BL-49-58-005	3,6-disubstituted indoles (**102-104**)	alkaloid	cytotoxic activity	74
	Streptomyces sp.	LA3L2	Bohemamine (**S46**), Bohemamine B (**S49**)	alkaloid	unknown	76
			S-methyl-2,4-dihydroxy-6-isopropyl-3,5-dimethylbenzothioate (**287**)	others	cytotoxic activity	
			Montagnetol (**S138**), Erythrin (**S139**)	others	unknown	
	Streptomyces sp.	LA3L1	Chromomycin A2 (**S140**), Chromomycin A3 (**S141**), Chromomycin 02-3D (**S142**)	others	unknown	76
	Streptomyces sp.	LA5L4	Thiazostatin B (**S50**)	alkaloid	unknown	76
	Saccharopolyspora sp.	SS081219 JE-28	JBIR-66 (**106**)	alkaloid	cytotoxic activity	64, 77
			Macrolactins E (**S89**) and F (**S90**), Gilvocarcins M (**S91**) and V (**S92**)	polyketide	unknown	
	Streptomyces sp.	1053U.I.1a.3b	Lobophorins I (**107**), F (**108**), B (**109**), C (**26**)	alkaloid	antibacterial activity, cytotoxic activity	78
			Lobophorin H (**S51**)	alkaloid	unknown	

Micromonospora sp.	29867	MBJ-0003 (**110**)	alkaloid	cytotoxicity	79
Actinoalloteichus sp.	PM0525875	Caerulomycin A (**111**)	alkaloid	antifungal activity	80
Micromonospora sp.		Keyicin (**112**)	alkaloid	antibacterial activity	81
Streptomyces sp.	G278	2,5-bis(5-(tert-butyl)benzo[d]oxazol-2-yl)thiophene (**113**), 2-methylpyridin-3-ol (**114**), N-phenylnaphthalen-2-amine (**115**), 2-(1H-indol-3-yl)ethan-1-ol (**58**)	alkaloid	antimicrobial activity	82
		(6R,8aS)-6-((1H-indol-3-yl)methyl)hexahydroindolizine-5,8-dione (**116**)	alkaloid	antibacterial and antifouling activities	
		1H-indole-3-carbaldehyde (**S21**), 1H-indole-3-carboxylic acid (**S52**)	alkaloid	unknown	
		Compound **205**	polyketide	antibacterial activity	
		7-hydroxy-6-methoxy-2H-chromen-2-one (**301**)	polyketide	antimicrobial activity	
		Benzyl 2-hydroxybenzoate (**300**)	others	antimicrobial activity	
Streptomyces sp.	ZZ406	1-acetyl-2-isobutyrylpyrazolidine-4-carboxylic acid (**S53**)	alkaloid	unknown	83
		5-hydroxy-4-(hydroxymethyl)-9,10-dioxo-9,10-dihydroanthracene-2-carboxylic acid (**216**), (R)-3-hydroxy-6-(2-methyl-4-oxo-4H-chromen-5-yl)-5-oxohexanoic acid (**217**)	polyketide	cytotoxic activity	
		Compounds **S93-S97**	polyketide	unknown	
		Acetyl-L-leucyl-L-seryl-L-alanine (**S130**)	peptide	unknown	
		Valinomycin (**230**)	peptide	antiparasite activity and	

				cytotoxic activity	
		GTRI-02 (**S164**)	others	unknown	
Streptomyces sp.	HDa1	Anthocidins A-D, Crassilin (**S54-S58**)	alkaloid	unknown	85, 86
		n-lauryl 5-hydroxyanthranilate (**118**), isolauryl 5-hydroxyanthranilate (**119**);	alkaloid	enzyme inhibitory activity	
		Benzamide (**S59**), Oxachelin (**S60**);	alkaloid	unknown	
		PD116740 (**126**);	polyketide	unknown	
		3-hydroxy-4-methoxycinnamamide (**120**);	alkaloid	antibacterial activity	
		Flavoside A (**S98**);	polyketide	unknown	
		(3S-cis)-hexahydro-3-[(3,4-dihydroxyphenyl)methyl]pyrrolo[1,2-a]pyrazine-1,4-dione (**274**)	peptides	antibacterial activity	
Streptomyces olivaceus	SCSIO LO13	Borrelidins M (**121**) and CR1 (**122**)	alkaloid	antibacterial activity, cytotoxic activity	87
		Borrelidins N,O,E,K (**S61-S64**)	alkaloid	unknown	
		Borrelidin A (**123**)	alkaloid	antibacterial, anti-parasite, cytotoxic activities	
Pseudonocardia sp.	HS7	Compounds **202**, **203** ,**204a** and **204c**	polyketide	anticancer activity	119
		Compounds **200**, **201**	polyketide	anticancer activity and antibacterial activity	
Saccharothrix espanaensis	An 113	X- 14881 E (**206**), ochromycinone (**207**), X-14881 C (**208**), saccharothrixmicines A (**209**);	polyketide	antibacterial activity	120, 121
		(3R,7R,8aS)-7-hydroxy-3-isobutylhexahydr opyrrolo[1,2-a]pyrazine-1,4-dione (**269**), (3S,7R,8aS)-7-hydroxy-3-isobutylhexahydro pyrrolo[1,2-a]pyrazine-1,4-dione (**270**), (3S,7R,8aR)-3-benzyl-7-hydroxyhexahydrop	peptide	antibiotic activities	

			yrrolo[1,2-a]pyrazine-1,4-dione (**271**);			
			X-14881 A (**288**), X-14881 B (**289**), Saccharothrixins A-C (**290-292**), Saccharothrixmicines B (**293**)	others	antibacterial activity	
	Streptomyces sp.	112CH148	Violapyrones H (**210**) and I (**211**)	polyketide	cytotoxic activity	122
			Violapyrones B (**212**) and C (**213**)	polyketide	cytotoxic activity and antibacterial activity	
	Streptomyces caniferus	GUA-06-05-006A	PM100117 (**214**) and PM100118 (**215**)	polyketide	cytotoxic activities and antifungal activity	123
	Streptomyces seoulensis	A01	Streptoseomycin (**117**)	alkaloid	antibacterial activities	84
	Streptomyces sampsonii	SCSIO 054	Julichromes Q11, Q12, Q10, Q6•6, Q6 (**218-222**)	polyketide	antibacterial activity	124
			Julichromes Q3•5 (**S99**), Q3•3 (**S100**), Chrysophanol (**S101**), 4-acetylchrysophanol (**S102**), Islandicin (**S103**), Huanglongmycin A (**S104**)	polyketide	unknown	
	Streptomyces sp.	CNB-091	Salinamides A (**266**) and B (**267**)	peptide	antibacterial activity and anti-inflammatory activity	149-151
			Salinamides C–E (**S127-S1229**)	peptide	unknown	
			Salinamide F (**268**)	peptide	antibacterial activity and enzyme inhibitory activity	
	Micromonospora sp.	ML1	Thiocoraline (**235**)	peptide	cytotoxic activity, enzyme inhibitory activity and antibacterial activity	152
	Streptomyces seoulensis	IFB-A01	Limazepines G (**272**) and H (**273**)	peptide	enzyme inhibitory activity	153
			Streptoseolactone (**284**)	steroid	enzyme inhibitory activity	
Fish	*Nocardiopsis dassonvillei*	RG-33B	Tetrodotoxin (**105**)	alkaloid	nonprotein neurotoxin	75
	Streptomyces	OUPS-N92	Halichomycin (**101**);	alkaloid	cytotoxicity	73

	hygroscopicus		Halichoblelide A-C (**223-225**)	polyketide	cytotoxicity	
	Streptomyces sp.	MNU FJ-36	2,5-diketopiperazines (2,5-DKPs) (**263-265**)	peptide	cytotoxicity	148
Marine mammals	*Micromonospora*		Phocoenamicin (**226**)	polyketide	antibacterial activity	127
Brown algae	*unidentified*	CNC-837	Lobophorins A (**306**) and B (**109**)	alkaloid	anti-inflammatory	167
	Streptomyces cyaneofuscatus	M-27	Daunomycin (**307**), Cosmomycin B (**308**)	alkaloid	antitumor antibiotics	168
			Maltophilin (**309**)	alkaloid	antifungal activity	
			Galtamycin B (**S181**)	polyketide	unknown	
	Streptomyces carnosus	M-40	Lobophorine B (**310**)	alkaloid	anti-inflammatory and antibacterial activity	168
			Germicidins A (**314**) and B (**315**)	polyketide	Spore germination and hypha elongation in *S. coelicolor*	
			Geosmin (**S195**), Beta-patchoulene (**S196**)	others	unknown	
	Nocardiopsis sp.	AS23C	4-amino-6-methylsalicylic acid (**311**)	alkaloid	antibacterial activity	169
			5-methylresorcinol (**339**), Linoleic acid (**340**)	others	antibacterial activities	
	Streptomyces sundarbansensis	WR1L1S8	Phaechromycins B, C and E (**S178-S180**)	polyketide	unknown	174
			2-hydroxy-5-((6-hydroxy-4-oxo-4H-pyran-2 -yl) methyl)-2-propylchroman -4-one (**313**)	polyketide	antibacterial activities	
	Micromonospora sp.	CNY-010	Neaumycin B (**316**)	polyketide	cytotoxicity	175
	Streptomyces praecox	291-11	BmDKP (**330**), ImDKP (**331**)	peptide	antifouling activity	179
	Streptomyces coelescens	PK206-15	Glycoglycerolipids **332-335**	others	antifouling activity	180

	Streptomyces atrovirens	PK288-21	2-hydroxy-5-(3-methylbut-2-enyl) benzaldehyde (**336**), 2-hepta-1,5-dienyl-3,6-dihydroxy-5-(3-methylbut-2-enyl) benzaldehyde (**337**)	others	antimicrobial activity	181
	Kocuria marina	CMG S2	4-[(Z)-2 phenyl ethenyl] benzoic acid (**338**)	others	antimicrobial activity	177
Green algae	*Streptomyces sp.*	HZP-2216E	Streptopertusacin A (**312**)	alkaloid	antibacterial activity	170, 171
			Streptoarylpyrazinone A (**S170**)	alkaloid	unknown	
			21,22-en-bafilomycin D (**318**), 21,22-en-9-hydroxybafilomycin D (**319**)	polyketide	cytotoxic activity and antibacterial activity	
			23-O-butyrylbafilomycin D (**317**)	polyketide	cytotoxicity	
			Bafilomycins D (**320**) and A1 (**322**)	polyketide	enzyme inhibitory activity, antibacterial, antifungal, insecticidal, herbicidal, and cytotoxic activities	
			9-hydroxybafilomycin D (**321**)	polyketide	antibacterial, antifungal, insecticidal, herbicidal, cytotoxic activities	
			Bafilomycin A2 (**S182**)	polyketide	unknown	
	Streptomyces sp.	ZZ502	Compounds **S171-S173**, 2-acetamido-3-hydroxybenzamide (**S174**), 2-amino-3-hydroxybenzamide (**S175**),and 2-aminobenzamide (**S176**)	alkaloid	unknown	172
	Streptomyces althioticus	MSM3	Desertomycin G (**323**)	polyketide	antibiotic activities and anti-tumor activity	176
Red alga	*Streptomyces ambofaciens*	BI0048	Vulgamycin (**324**)	polyketide	herbicidal activity and antibacterial activity	177
			5-deoxy-enterocin (**325**)	polyketide	antibacterial activity	
			Germicidin A (**314**)	polyketide	autoregulative inhibitor of	

					spore germination, antibacterial activity	
			Germicidin B (**315**)	polyketide	unknown	
			Zoumbericins A, B, Germicidins K, L, Wailupemycins D, E (**S183-S188**)	polyketide	unknown	
			Benzoic acid (**S158**), Hydrocinnamic acid (**S193**), (*E*)-cinnamic acid (**S194**)	others	unknown	
Cyanobacteria	*Streptomyces sp.*	N1-78-1	Bisanthraquinones **326**, **327**	polyketide	antibacterial activity and cytotoxicity	112
			Bisanthraquinone **328**	polyketide	cytotoxicity	
Lichens	*Streptomyces cavourensis*	YY01-17	(E)-3-hydroxy-2,4-dimethylhept-4-enamide (**S177**)	alkaloid	unknown	173
			3-hydroxybutan-2-yl (2S)-2-hydroxypropanoate (**S191**)	others	unknown	
			2-hydroxy-3-methylbutanoic acid (**S192**)	others	unknown	
Marine plants	*Streptomyces sp.*	FX-58	Anthraquinone **329**	polyketide	cytotoxic activities	178
			Octadecanoic acid (**S189**)	others	unknown	
			Cholest-4-en-3-one (**S190**)	others	unknown	

4. Table S8. Summarized repetitive compounds identified from multiple actinomycetes.

**Compounds** / Structural types	**Actinomycetes**	**Host**	**References**
Staurosporine(**3**) alkaloid	*Streptomyces* sp. 11	Sponge	9, 21
	Micromonospora sp. L-31-CLCO-002	Sponge	9, 10
	Streptomyces sp. LS298	Sponge	36
Diazepinomicin(**28**) alkaloid	*Micromonospora* sp. RV115	Sponge	9, 28
	Micromonospora sp. DPJ12	Ascidian	67

lobophorin C(**26**)	*Streptomyces carnosus* AZS17	Sponge	27
alkaloid	*Streptomyces* sp. 1053U.I.1a.3b	*Lienardia Totopotens*	78
tirandamycin A(**43**)	*Streptomyces* sp. LS298	Sponge	36
tirandamycin B(**44**)	*Streptomyces tirandamycinicus* HNM0039T	Sponge	49
alkaloid	*Streptomyces* sp. SCSIO 41399	Coral	62
indole-3-acetic acid(**S16**) alkaloid	*Streptomyces* sp. G246	Sponge	56
Tryptophan(**S160**) amino acid	*Rhodococcus* sp. UA13	Sponge	43
1H-indole-3-carbaldehyde(**S21**)	*Saccharomonospora* sp. UR22	Sponge	47
alkaloid	*Streptomyces* sp. G278	Cucumber	82
9H-pyrido[3,4-b]indole(**S30**) alkaloid	*Streptomyces* sp. G246	Sponge	56
cyclo(L-Pro-L-Tyr)(**S120**) peptide	*Streptomyces* sp. G248	Sponge	55
cyclo (L-Pro-L-Leu)(**246**)	*Streptomyces* sp. G248	Sponge	55
peptide	*Streptomyces* sp. Did-27	Ascidian	64, 72
cyclo(L-Pro-L-Phe)(**S123**)	*Streptomyces* sp. G248	Sponge	55
peptide	*Streptomyces* sp. Call-36	Sponge	143
PD116740(**126**)	*Saccharopolyspora taberi* PEM-06-F23-019B	Sponge	91
polyketide	*Streptomyces* sp. HDa1	Urchin	85, 86
cyclo (6-OH-D-Pro-L-Phe)(**229**)	*Streptomyces* sp. DA18	Sponge	128
peptide	*Streptomyces* sp. Did-27	Ascidian	64, 72
Valinomycin(**230**)	*Streptomyces* sp. 22	Sponge	9, 21
peptide	*Streptomyces* sp. 34	Sponge	9, 21
	Streptomyces sp. ZZ406	Sea anemones	83
Thiocoraline(**235**)	*Verrucosispora* sp. WMMA107	Sponge	9, 132
peptide	*Micromonospora* sp. L-13-ACM2-092	Coral	145, 146
	Micromonospora sp. ML1	Jellyfish	152
Bohemamine(**S46**)	*Streptomyces* sp.	Ascidian	64
alkaloid	*Streptomyces* sp. LA3L2	Marine invertebrates	76

lobophorin B(**109**) alkaloid	*Streptomyces* sp. 1053U.I.1a.3b<br>unidentified CNC-837	Lienardia totopotens<br>Brown algae	78<br>167
2-(1H-indol-3-yl)ethan-1-ol(**58**) alkaloid	*Saccharomonospora* sp. UR22<br>*Streptomyces* sp. G278	Sponge<br>Cucumber	47<br>82
3-hydroxy-2-methyl-4H-pyran-4-one (maltol)(**S74**) polyketide	*Streptomyces* sp. SBT348<br>*Rhodococcus* sp. UA13	Sponge<br>Sponge	45<br>43
Germicidins A and B (**314**, **315**) polyketide	*Streptomyces carnosus* M-40<br>*Streptomyces ambofaciens* BI0048	Brown algae<br>Red algae	168<br>177
Phencomycin, tubermycin B (**47**, **48**) alkaloid	*Streptomyces* sp. SBT345<br>*Streptomyces* sp. RM66	Sponge<br>Sponge	40, 41<br>58
Actinosporins C, D, G (**144**,**145**,**155**) polyketide	*Actinokineospora spheciospongiae sp.* nov.<br>*Actinokineospora spheciospongiae* EG49	Sponge<br>Sponge	52<br>59, 100, 101
Benzoic acid (**S158**) acid	*Streptomyces griseorubens sp.* ASMR4<br>*Streptomyces ambofaciens* BI0048	Coral<br>Red algae	164<br>177
